# Enantioselective Access to Trifluoromethylated Spirocyclopropyl Heterocycles

**DOI:** 10.1002/advs.77226

**Published:** 2026-08-18

**Authors:** Yuanhao Zhu, Youzhi Xu, Weitao Zhang, Yongyue Ning, Yaojia Jiang, Yongquan Ning, Genping Huang, Jiawei Cheng, Paramasivam Sivaguru, Xihe Bi

**Affiliations:** ^1^ State Key Laboratory of Green Pesticide Guizhou University Guiyang China; ^2^ Department of Chemistry School of Science Tianjin University Tianjin China; ^3^ Department of Chemistry Northeast Normal University Changchun China; ^4^ State Key Laboratory of Elemento‐Organic Chemistry Nankai University Tianjin China

**Keywords:** [2+1] cycloaddition, asymmetric catalysis, carbenes, spirocyclopropyl heterocycles, triftosylhydrazones

## Abstract

Chiral spirocyclopropyl heterocycles have emerged as privileged three‐dimensional structural motifs in medicinal chemistry. Nevertheless, efficient catalytic enantioselective assembly of such compounds still faces considerable synthetic difficulties. Herein, we describe a rhodium‐mediated asymmetric [2+1] cyclopropanation reaction using methylene azacycles and fluoroalkyl triftosylhydrazones as reaction partners, which offers a concise synthetic route toward enantioenriched trifluoromethylated spirocyclopropyl heterocyclic derivatives. This synthetic transformation tolerates various substrates and functional groups, and achieves remarkable stereochemical control. Multiple spirocyclic skeletons can be efficiently constructed with favorable yields and prominent diastereo‐ and enantioselectivity, with the highest e.e. value recorded at 99%. The acquired spirocyclopropyl oxazolidinone derivatives can be further derivatized via internal reactive moieties, which allows their integration into bioactive molecular frameworks. By combining fluoroalkyl carbenes with asymmetric spirocyclopropanation, the present work develops a general catalytic system to synthesize elusive fluoroalkyl‐bearing chiral spirocyclopropyl heterocycles.

## Graphical abstract

We report a rhodium‐catalyzed asymmetric [2+1] spirocyclopropanation of methylene azacycles with trifluoromethyl carbenes in situ derived from fluoroalkyl triftosylhydrazones.

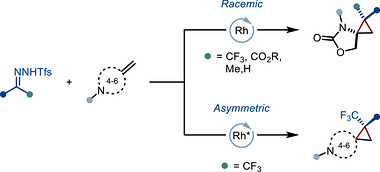

## 1 Introduction

**FIGURE 1:**
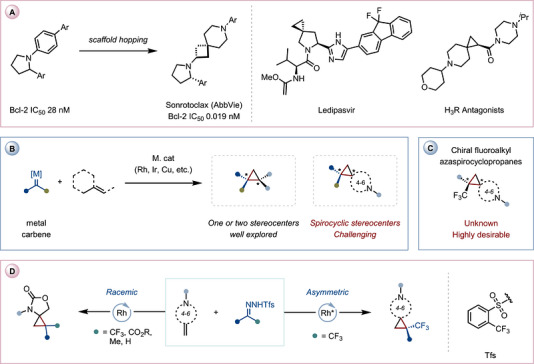
Background and reaction design. (A). Spirocyclic heterocycles as rigid, three‐dimensional alternatives to aromatic linkers, and representative bioactive molecules featuring spirocyclopropyl heterocycle motifs. (B). Transition metal‐catalyzed asymmetric cyclopropanation reactions. (C). Chiral fluoroalkyl azaspirocyclopropanes as highly desirable yet unexplored scaffolds. (D). Enantioselective access to trifluoromethylated spirocyclopropyl heterocycles (*This work*).

Spirocyclic heterocycles, particularly nitrogen‐containing analogs, serve as privileged structural cores in drug discovery. Their outstanding pharmacokinetic and physicochemical properties endow these skeletons with high bioactivity, desirable lipophilicity, precise target selectivity, and robust metabolic stability (Figure 1A, left) [1, 2, 3, 4, 5, 6, 7]. Featuring rigid and conformationally restricted three‐dimensional skeletons, these scaffolds offer abundant spatial vectors for molecular recognition, thus achieving stronger binding interactions with biological targets than conventional planar analogues [8, 9, 10, 11]. Among these, spirocyclopropyl heterocycles have gained remarkable research interest [12, 13]. Such structural motifs occupy a vital position in modern drug discovery, possessing abundant and promising biological activities (Figure 1A, right) [14, 15]. Notably, the number of such substructures indexed in the Reaxys database increased dramatically from just over 10,000 in 2023 to nearly 90,000 in 2025 [16]. Despite such rapid advances, most reported structures contain simple, unsubstituted cyclopropane skeletons, as synthetic routes toward chiral spirocyclopropyl heterocycles remain poorly investigated [17]. Conventional access to enantioenriched spirocyclopropyl heterocycles has relied on chiral chromatographic separation, which suffers from a costly, time‐consuming, and resource‐intensive process [18]. Therefore, the development of efficient, direct, and catalytic asymmetric strategies for constructing structurally diverse chiral spirocyclopropyl heterocycles from readily available starting materials is highly desirable.

The [2+1] cycloaddition of alkenes with carbenes or metal–carbenoid intermediates represents a cornerstone strategy for the stereoselective construction of chiral cyclopropane scaffolds (Figure 1B) [19, 20, 21, 22, 23]. Moreover, organocatalysis has emerged as a robust and complementary platform for the asymmetric construction of cyclopropyl spiroheterocyclic frameworks [24, 25, 26]. Owing to its high chemo‐, regio‐, and enantioselectivity across diverse alkene substrates, this transformation provides rapid access to strained three‐membered rings and offers versatile opportunities for downstream functionalization and scaffold diversification [27, 28]. Nevertheless, the asymmetric catalytic construction of spirocyclic quaternary stereocenters through such [2+1] cycloaddition manifolds remains underdeveloped, mainly attributed to the intrinsic steric hindrance and rigid conformation of spiro frameworks [29, 30, 31]. Given the persistent challenges associated with chiral spirocyclopropyl heterocycles [17] and continuing interest in the carbene chemistry of triftosylhydrazone [32, 33, 34], we envisioned that fluoroalkyl carbene species could engage methylene azacycles in a selective [2+1] cyclopropanation, thereby providing direct access to trifluoromethylated spirocyclopropyl heterocycles [35, 36]. The trifluoromethyl‐substituted cyclopropane frameworks are particularly attractive in medicinal chemistry, as this motif can substantially modulate physicochemical properties, metabolic stability, pharmacokinetic profiles, and overall drug‐like character [37, 38, 39, 40]. Despite this recognized value, catalytic enantioselective approaches to structurally diverse trifluoromethylated spirocyclopropyl heterocycles remain unknown [41], limiting their broader exploration in medicinal chemistry and drug discovery (Figure 1C). During the course of this ongoing study, Arnold and Hartwig independently reported biocatalytic stereoselective cyclopropanation of unsaturated exocyclic heterocycles using engineered hemoprotein or iridium‐containing cytochrome, affording enantioenriched azaspiro[2.y]alkanes [42, 43]. Complementary to these advances in biocatalysis, the Davies group created an elegant asymmetric cyclopropanation strategy, wherein azacyclomethylidenes, diazolactones and diazolactams act as reactive partners [44, 45]. After extensive reaction screening and optimization, we established the first cyclopropanation of methylene 2‐oxazolidones with metal carbenes, delivering previously inaccessible cyclopropane‐fused spirooxazolidinones in high yields and excellent diastereoselectivity (Figure 1D, left). We further achieved a catalytic asymmetric variant enabled by chiral dirhodium catalysts [46, 47]. This method exhibits excellent enantiocontrol in the cyclopropanation of diverse methylene azacycles, affording a variety of enantioenriched trifluoromethylated spirocyclopropyl heterocycles, including spirooxazolidinones, spiroazetidines, spiropyrrolidines, and spiropiperidines, with enantioselectivities of up to 99% e.e. (Figure 1D, right).

## 2 Results and Discussion

**FIGURE 2:**
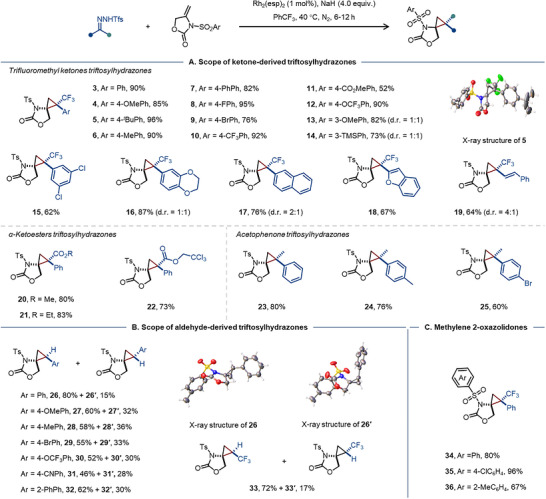
Substrate scope investigation. Reaction conditions: Triftosylhydrazones (0.2 mmol, 1.0 equiv.), methylene 2‐oxazolidones (0.3 mmol, 1.5 equiv.), Rh_2_(esp)_2_ (1 mol%), NaH (0.8 mmol, 4.0 equiv.), PhCF_3_ (5.0 mL), 40°C under N_2_ for 6–12 h. Unless otherwise specified, all products were obtained with d.r. > 20:1 as determined by **^1^H NMR** spectroscopy.

We began our investigation using trifluoroacetophenone‐derived triftosylhydrazone **1a** and methylene 2‐oxazolidone **2** as the reaction partners to optimize the diastereoselective cyclopropanation reaction. Through systematic screening of reaction parameters, we identified Rh_2_(esp)_2_ (1 mol%) as the optimal catalyst and NaH (4.0 equiv.) as the base in dichloromethane under a nitrogen atmosphere at 40°C for 8 h, affording the desired spirocyclopropyl oxazolidinone **3** in 90% yield (see Table S1 for full details). With optimized conditions established, we next explored the substrate generality of this transformation (Figure 2A). Keeping methylene oxazolidone **2** as the benchmark substrate, we first examined the scope of various triftosylhydrazones. As shown in Figure 2A, a range of trifluoroacetophenone‐derived triftosylhydrazones bearing either electron‐donating or electron‐withdrawing substituents at the *para*‐position of the aryl ring underwent smooth cyclopropanation to furnish spirocyclopropane products **4–12** in 52–96% yields. Analogous reactivity was observed for *meta*‐substituted and 3,5‐disubstituted aryl triftosylhydrazones, affording products **13–15** with comparable yields. A range of functional groups on the arene rings, including fluoro, bromo, trifluoromethyl, ester, and trimethylsilyl, proved compatible with this protocol, providing opportunities for downstream diversifications. Even sterically demanding α‐trifluoromethyl triftosylhydrazones featuring fused (hetero)aromatic rings, such as 1,4‐benzodioxane (**16**), naphthalene (**17**), and benzofuran (**18**), underwent cyclopropanation in excellent yields. A substrate with a vinyl‐containing ancillary group was also converted to product **19** in 67% yield with a diastereomeric ratio of 4:1. The scope of carbene precursors was further extended to include α‐ketoester‐derived triftosylhydrazones, which underwent the smooth transformation to afford products **20–22** in 73–83% yields. Notably, acetophenone‐derived triftosylhydrazones, serving as donor–donor carbenes, also proved highly effective, affording products **23–25** in high yields, further underscoring the broad substrate tolerance, functional group compatibility, and robustness of the catalytic system. The stereochemical assignments across the series of products were based on single‐crystal X‐ray diffraction analysis of product **5**.

Subsequently, we examined the reactivity of aldehyde‐derived triftosylhydrazones in this transformation with methylene 2‐oxazolidone (Figure 2B). A series of aryl triftosylhydrazones functionalized with methoxy, methyl, bromo, trifluoromethoxy, cyano, and phenyl substituents on the aromatic ring all proved compatible as donor‐only carbenes, delivering a mixture of diastereomeric products **26–32** and **26'–32'** in 74–95% combined yields with up to 5.3:1 dr. Similarly, an acceptor‐only carbene, trifluoroacetaldehyde triftosylhydrazone (TFHZ‐Tfs), underwent cyclopropanation efficiently to generate products **33** and **33'** in 89% yield. The relative stereochemistry of these diastereomers was unambiguously assigned by single‐crystal X‐ray diffraction analysis of **26** and **26'**. Finally, we sought to test the scope of methylene 2‐oxazolidones using trifluoroacetophenone‐derived triftosylhydrazone as the carbene precursor (Figure 2C). For example, varying the sulfonyl protecting group on the methylene 2‐oxazolidones, either phenylsulfonyl or 4‐chlorophenylsulfonyl groups, had little impact on reaction efficiency, furnishing products **34** and **35** in 80 and 96% yields, respectively. In contrast, an *ortho*‐tosyl‐protected methylene 2‐oxazolidone led to a modest decrease in yield (67% for **36**).

**FIGURE 3:**
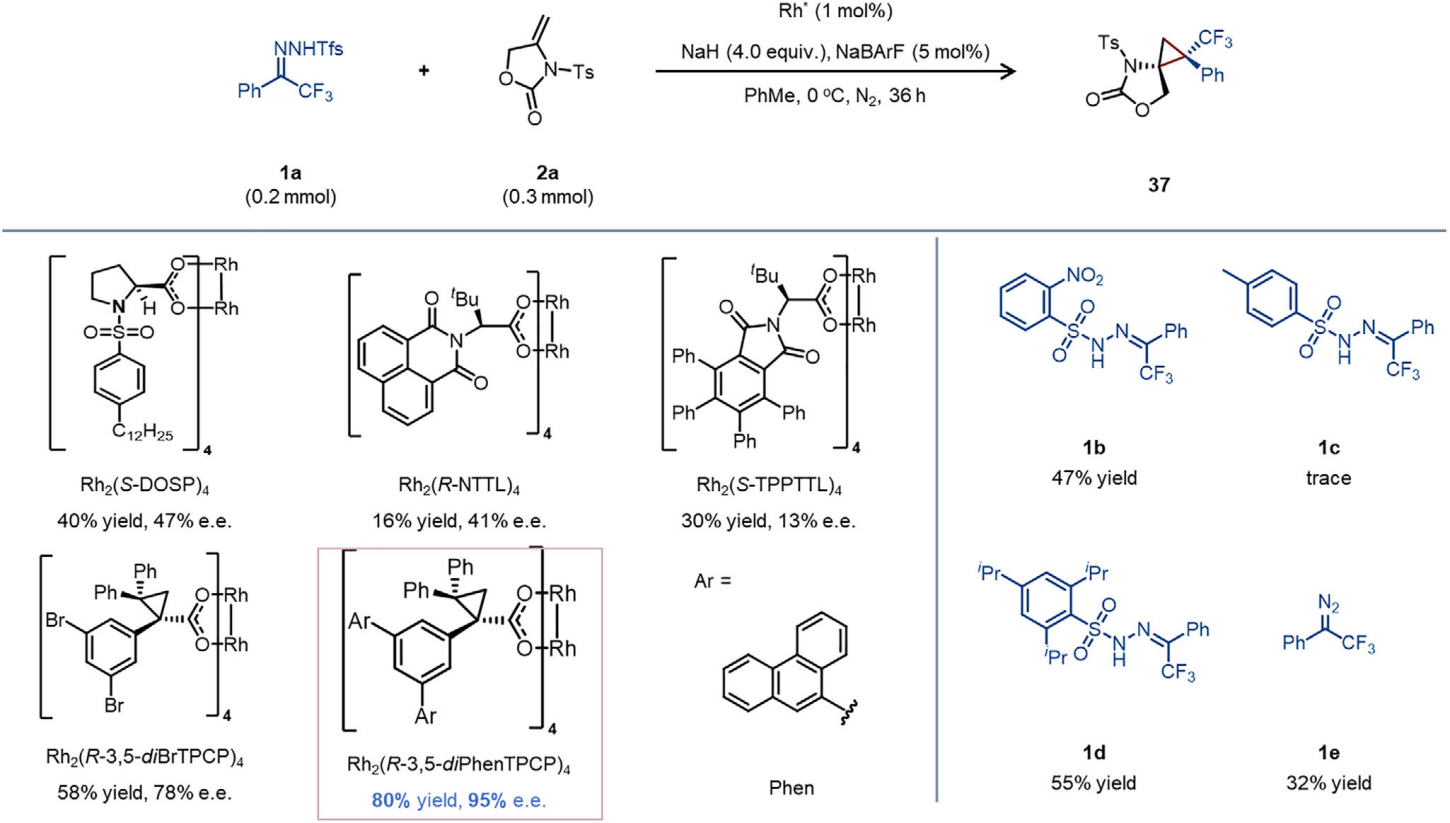
Optimization of reaction conditions for the asymmetric version. Reaction conditions: **1** (0.2 mmol, 1.0 equiv.), methylene oxazolidone (**2**, 0.3 mmol, 1.5 equiv.), Rh^*^(1 mol%), NaH (0.8 mmol, 4.0 equiv.), NaBArF (5.0 mol%), and toluene (5.0 mL) at 0°C under N_2_ for 36 h. The e.e. values were determined by chiral HPLC analysis. Isolated yields are shown.

Motivated by the preceding results, we then pursued an enantioselective variant of this transformation to access enantioenriched spirocyclopropyl oxazolidinones. Our optimization began with screening various chiral dirhodium catalysts in the model reaction between methylene 2‐oxazolidone **2** and triftosylhydrazone **1a**, using NaH (4.0 equiv.) as base in toluene at 0°C under N_2_ atmosphere for 36 h (Figure 3). Initial experiments with common chiral dirhodium catalysts, including Rh_2_(*S*‐DOSP)_4_, Rh_2_(*R*‐NTTL)_4_, and Rh_2_(*S*‐TPPTTL)_4_, afforded the target enantioenriched spirocyclopropyl oxazolidinone **37** in low yields and poor enantioselectivity. Notably, the chiral dirhodium catalyst bearing triarylcyclopropane carboxylate (TPCP) ligands showed marked improvement: Rh_2_(*R*‐3,5‐*di*BrTPCP)_4_ raised the enantioselectivity to 78%, albeit with only moderate yield (58%). Gratifyingly, the newly designed Rh_2_(*R*‐3,5‐*di*PhenTPCP)_4_, featuring bulky 9‐phenanthrenyl substituents on the TPCP ligand, emerged as an optimal catalyst, furnishing **37** in 80% isolated yield with 92% enantiomeric excess. We further assessed alternative carbene precursors under identical conditions. While *N*‐nosylhydrazone (**1b**), *N*‐trisylhydrazone (**1d**), and α‐trifluoromethyl phenyldiazomethane (**1e**) afforded **37** in low yields (32%–55%), *N*‐tosylhydrazone (**1c**) proved completely ineffective.

**FIGURE 4:**
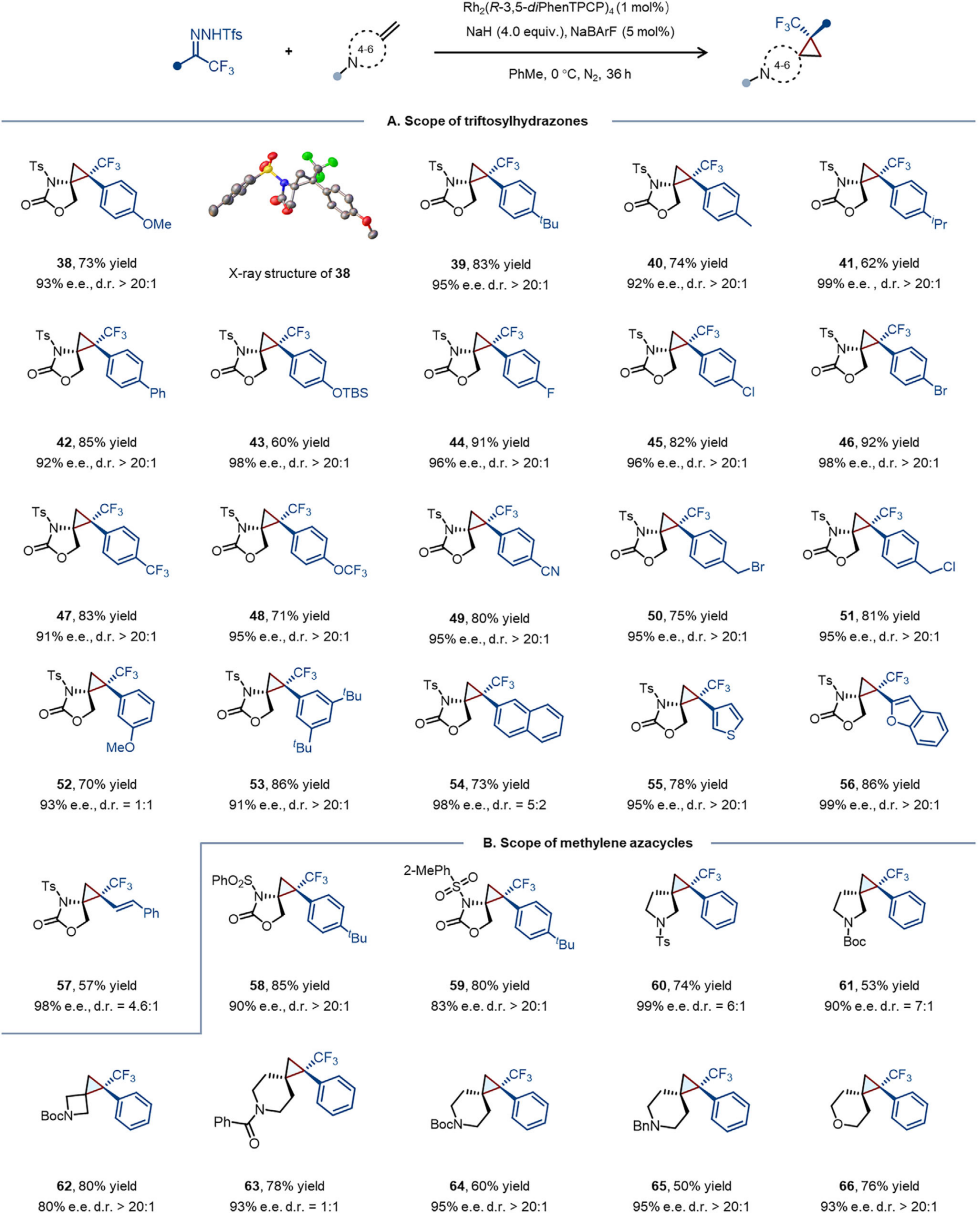
Substrate scope investigation for the asymmetric version. Reaction conditions: Triftosylhydrazones (0.2 mmol, 1.0 equiv.), methylene azacycles (0.3 mmol, 1.5 equiv.), Rh_2_(*R*‐3,5‐*di*PhenTPCP)_4_ (1 mol%), NaH (0.8 mmol, 4.0 equiv.), NaBArF (5.0 mol%), toluene (5.0 mL), 0°C under N_2_ for 36 h. The e.e. values were determined by chiral HPLC analysis. Isolated yields are shown.

We then examined the scope of this enantioselective cyclopropanation, initially focusing on triftosylhydrazones using methylene oxazolidone **2** as the representative substrate (Figure 4). Our catalytic system exhibited broad compatibility toward various electronically differentiated *para*‐substituted aryl trifluoromethyl triftosylhydrazones (Figure 4A), with methoxy, *tert*‐butyl, methyl, isopropyl, silyl ether, and alkyl halides, halogens, trifluoromethyl, trifluoromethoxy, and cyano functionalities, affording corresponding chiral spirocyclopropyl oxazolidinones (**38–51**) in high yields with excellent enantioselectivities (87–96% e.e.). The absolute configuration of representative product **38** was unambiguously determined by single‐crystal X‐ray diffraction analysis. Moreover, meta‐ and 3,5‐disubstituted aryl triftosylhydrazones also reacted efficiently, delivering chiral spirocyclic products with high enantiocontrol (**52** and **53**). Substrates bearing extended π‐systems, including naphthalene (**54**, 5:2 d.r.), thiophene (**55**), and benzofuran (**56**) derivatives, underwent smooth cyclopropanation to give the desired spirocyclopropyl oxazolidinones with excellent enantioselectivities. Notably, an α‐trifluoromethyl vinyl triftosylhydrazone also proved viable, furnishing spirovinylcyclopropane **57** in 57% yield with excellent enantioselectivity (98% e.e.) and moderate diastereoselectivity (4.6:1 d.r.).

Subsequently, the substrate scope of methylene oxazolidones was investigated using *para*‐*tert*‐butylphenyl trifluoromethyl triftosylhydrazone as the carbene precursor (Figure 4B). Methylene 2‐oxazolidones bearing phenylsulfonyl or *ortho*‐tosyl protecting groups were efficiently converted to products **58** and **59** in 85% and 80% yields, with 90% e.e. and 83% e.e., respectively. The transformation was then extended beyond oxazolidones to a range of methylene‐containing nitrogen heterocycles using phenyl trifluoromethyl triftosylhydrazone. For example, *N*‐Ts‐ and *N*‐Boc‐protected pyrrolidines furnished **60** and **61** in 74% and 53% yields with excellent enantioselectivities and moderate to good diastereoselectivities, while an azetidine substrate afforded **62** in 80% yield and 80% e.e. Piperidine derivatives bearing benzoyl, Boc, or benzyl substituents delivered **63–65** in 50–78% yields with uniformly high enantioselectivities (93–95% e.e.). A morpholine‐derived substrate was also well tolerated, giving **66** in 76% yield with 93% e.e. and >20:1 d.r. Collectively, these results establish this catalytic system as a general platform for accessing structurally diverse and previously underexplored trifluoromethylated chiral spirocyclopropyl heterocycles.

**FIGURE 5:**
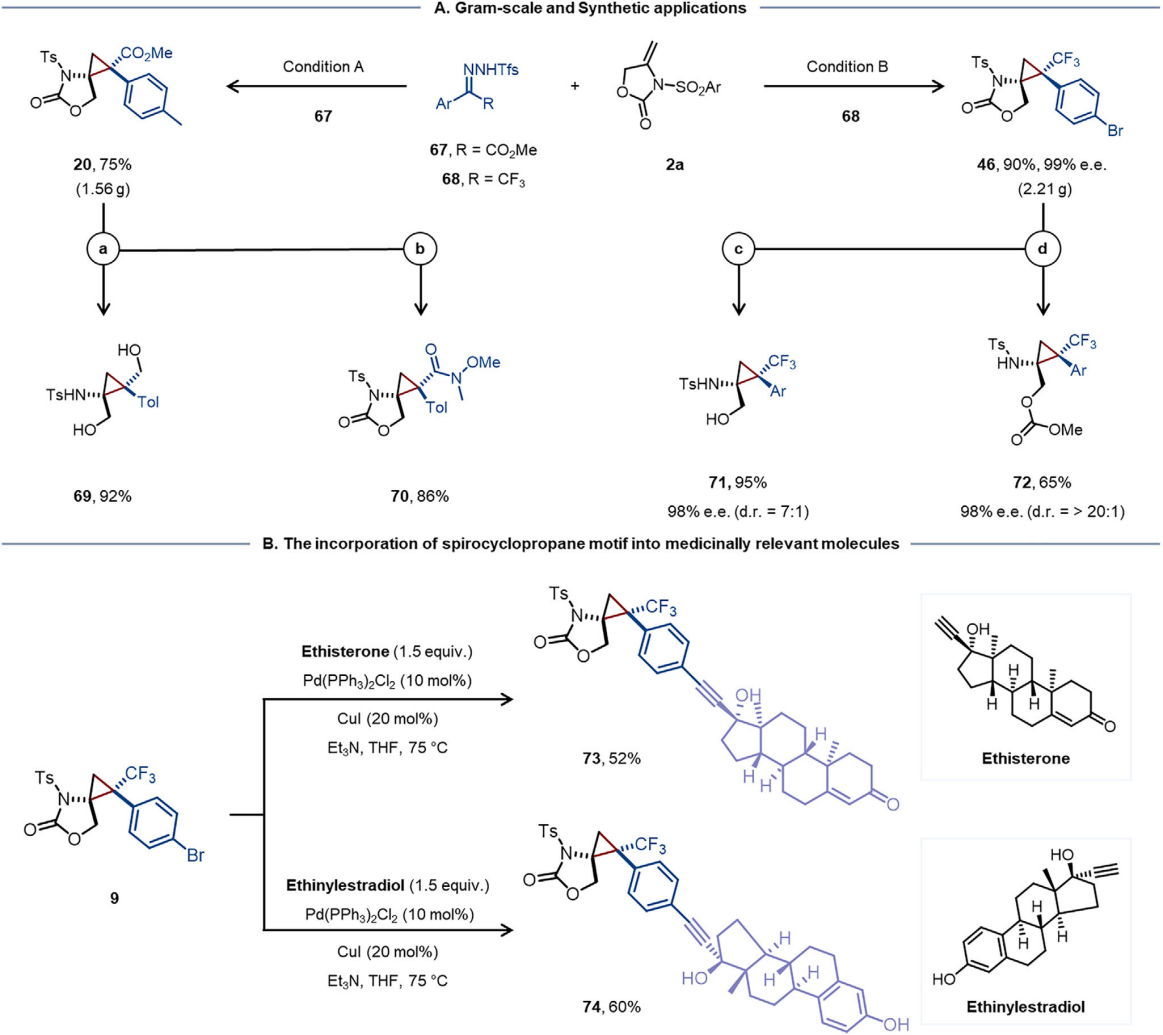
Synthetic applications and incorporation of the spirocyclopropane motif into medicinally relevant molecules. Conditions A: **2a** (5.0 mmol, 1.0 equiv.), **67** (7.5 mmol, 1.5 equiv.), Rh_2_(esp)_2_ (1 mol%), NaH (30.0 mmol, 6.0 equiv.), PhCF_3_ (125 mL), 40°C under N_2_ for 12 h. Conditions B: **2a** (5.0 mmol, 1.0 equiv.), **68** (7.5 mmol, 1.5 equiv.), Rh_2_(*R*‐3,5‐*di*PhenTPCP)_4_ (1 mol%), NaH (30.0 mmol, 6.0 equiv.), PhCF_3_ (125 mL), 40°C under N_2_ for 12 h. Product diversifications: a) LiAlH_4_ (2.0 equiv.), THF, 0°C. b) NHMe(OMe)·HCl (1.2 equiv.), *^i^* PrMgCl (2.4 equiv.), THF, N_2_, 0°C. c) LiAlH_4_ (4.0 equiv.), THF, 0°C. d) Mg (50.0 equiv.), MeOH, 0°C—r.t.

To further showcase the synthetic value of this protocol, we successfully scaled up the cyclopropanation of methylene 2‐oxazolidone **2** with either methyl 2‐oxo‐2‐(*p*‐tolyl)acetate‐derived triftosylhydrazone (**67**) or 4‐bromo‐trifluoroacetophenone‐derived triftosylhydrazone (**68**) to the gram scale level. The corresponding spirocyclopropyl oxazolidinones **20** and **46** were isolated in yields comparable to those obtained in standard small‐scale reactions, demonstrating the potential of this methodology for large‐scale synthesis (Figure 5A). Next, we explored synthetic transformations of the newly formed cycloadducts. Reduction of spirocyclopropyl oxazolidinones **20** and **46** with lithium aluminum hydride (LiAlH_4_) cleanly afforded the respective cyclopropyl methanol derivatives **69** and **71** in 92% and 95% yields, respectively [48]. Treatment of compound **20** in the presence of *N*, *O*‐dimethylhydroxylamine hydrochloride generated the spirocyclopropyl oxazolidinone‐derived Weinreb amide **70** in 86% yield [49]. The methanolysis of compound **46** led to the formation of cyclopropylamine methyl carbonate **72** in 65% yield. Moreover, the aryl bromide in spirocyclopropane **9** undergoes Pd‐catalyzed Sonogashira cross‐coupling with ethisterone or ethinylestradiol to deliver corresponding steroid‐conjugated products **73** and **74** in 52% and 60% yields, respectively (Figure 5B). The successful transformations into diverse functional groups and the incorporation of bioactive compounds further underscore the broad applicability of spirocyclopropyl oxazolidinone motifs.

**FIGURE 6:**
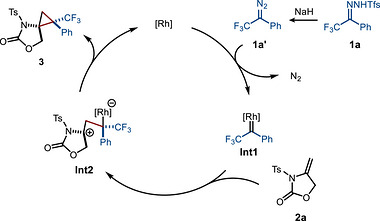
A plausible mechanism.

Based on prior literature [32], we propose a plausible mechanistic pathway for this transformation (Figure 6). Initially, triftosylhydrazone **1a** decomposes in the presence of NaH at ambient temperature to generate diazo species **1a'**, which then reacts smoothly with the rhodium catalyst to form the electrophilic rhodium carbene intermediate **Int1**. Nucleophilic attack by the electron‐rich exocyclic C═C bond of **2a** on the carbene carbon of **Int1** affords a rhodium‐coordinated zwitterionic intermediate **Int2**. Subsequent intramolecular C─C bond formation within **Int2** proceeds with concomitant release of the rhodium catalyst, delivering the densely functionalized chiral cyclopropane product **3**.

## 3 Conclusion

We have developed a versatile and modular rhodium‐catalyzed cyclopropanation strategy, which constitutes the first synthetic protocol for the construction of trifluoromethylated spirocyclopropyl heterocycles using readily accessible methylene azacycles and triftosylhydrazone precursors. This method allows the efficient fabrication of a wide array of three‐dimensional spirocyclic scaffolds covering spirooxazolidinone, spiroazetidine, spiropyrrolidine, spiropiperidine, and morpholine‐derived structural motifs. The synthetic value and medicinal applicability of this reaction are evidenced by its broad substrate scope, exceptional functional‐group tolerance, feasible gram‐scale preparation, flexible downstream derivatization, and successful structural modification of bioactive molecules. Beyond the synthetic innovation, this work opens a practical avenue for the synthesis of fluoroalkylated chiral spirocyclopropane skeletons with promising prospects in medicinal chemistry and drug discovery.

## Author Contributions

**Yongyue Ning**: methodology. **Genping Huang**: resources, methodology. **Xihe Bi**: writing – **Yuanhao Zhu**: methodology. **Youzhi Xu**: methodology. **Weitao Zhang**: methodology. **Yongyue Ning**: methodology. **Yaojia Jiang**: writing – review and editing, writing – original draft. **Genping Huang**: writing – review and editing, formal analysis. **Yongquan Ning**: methodology, writing – review and editing, writing – original draft, formal analysis. Jiawei Cheng: methodology. **Paramasivam Sivaguru**: methodology, writing – review and editing. **Xihe Bi**: writing – review and editing, writing – original draft, funding acquisition, supervision, investigation.

## Conflicts of Interest

The authors declare no conflicts of interest.

## Supporting information




**Supporting File**: advs77226‐sup‐0001‐SuppMat.docx.

## Data Availability

The data that support the findings of this study are available in the Supporting Information of this article.

## References

[1] K. Hiesinger , D. Dar'in , E. Proschak , and M. Krasavin , “Spirocyclic Scaffolds in Medicinal Chemistry,” Journal of Medicinal Chemistry 64 (2021): 150–183, 10.1021/acs.jmedchem.0c01473. 33381970

[2] A. A. Kirichok , H. Tkachuk , K. Levchenko , et al., ““Angular” Spirocyclic Azetidines: Synthesis, Characterization, and Evaluation in Drug Discovery,” Angewandte Chemie International Edition 64 (2025): e202418850, 10.1002/anie.202418850. 39621438

[3] M. Kamlar , M. Urban , and J. Veselý , “Enantioselective Synthesis of Spiro Heterocyclic Compounds Using a Combination of Organocatalysis and Transition‐Metal Catalysis,” The Chemical Record 23 (2023): e202200284, 10.1002/tcr.202200284. 36703545

[4] S. L. Degorce , M. S. Bodnarchuk , and J. S. Scott , “Lowering Lipophilicity by Adding Carbon: AzaSpiroHeptanes, a logD Lowering Twist,” ACS Medicinal Chemistry Letters 10 (2019): 1198–1204, 10.1021/acsmedchemlett.9b00248. 31417667 PMC6693472

[5] V. Kubyshkin and P. K. Mykhailiuk , “Smallest Bicycles in Medicinal Chemistry: Where Are We Now?,” Chemical Reviews 126 (2026): 3829–3882, 10.1021/acs.chemrev.5c00779. 41818297

[6] R. Crawshaw , R. Smithson , J. Hofer , et al., “Efficient and Selective Energy Transfer Photoenzymes Powered by Visible Light,” Nature Chemistry 17 (2025): 1083–1090, 10.1038/s41557-025-01820-0. PMC12226345 40329013

[7] J. A. Burkhard , B. Wagner , H. Fischer , F. Schuler , K. Müller , and E. M. Carreira , “Synthesis of Azaspirocycles and Their Evaluation in Drug Discovery,” Angewandte Chemie International Edition 49 (2010): 3524–3527, 10.1002/anie.200907108. 20544904

[8] F. Lovering , J. Bikker , and C. Humblet , “Escape From Flatland: Increasing Saturation as an Approach to Improving Clinical Success,” Journal of Medicinal Chemistry 52 (2009): 6752–6756, 10.1021/jm901241e. 19827778

[9] P. K. Mykhailiuk , “Saturated Bioisosteres of Benzene: Where to Go Next?,” Organic & Biomolecular Chemistry 17 (2019): 2839–2849, 10.1039/C8OB02812E. 30672560

[10] A. Malashchuk , A. V. Chernykh , M. Y. Perebyinis , I. V. Komarov , and O. O. Grygorenko , “Monoprotected Diamines Derived From 1,5‐Disubstituted(aza) Spiro[2.3]Hexane Scaffolds,” European Journal of Organic Chemistry 2021 (2021): 6570–6579, 10.1002/ejoc.202001614.

[11] J. Tsien , C. Hu , R. R. Merchant , and T. Qin , “Three‐Dimensional Saturated C(sp3)‐Rich Bioisosteres for Benzene,” Nature Reviews Chemistry 8 (2024): 605–627, 10.1038/s41570-024-00623-0. 38982260 PMC11823177

[12] H. M. Patel , C. D. Patel , F. Damiri , M. P. Parmar , D. P. Vala , and M. Berrada , “Synthesis of Spirocyclopropane Scaffolds: A Review,” Synthesis 57 (2025): 2131–2154, 10.1055/s-0043-1775471.

[13] R. Moorthy , W. Bio‐sawe , S. S. Thorat , and M. P. Sibi , “Unveiling the Beauty of Cyclopropane Formation: A Comprehensive Survey of Enantioselective Michael Initiated Ring Closure (MIRC) Reactions,” Organic Chemistry Frontiers 11 (2024): 4560–4601, 10.1039/D4QO00535J.

[14] J. O. Link , J. G. Taylor , L. Xu , et al., “Discovery of Ledipasvir (GS‐5885): A Potent, Once‐Daily Oral NS5A Inhibitor for the Treatment of Hepatitis C Virus Infection,” Journal of Medicinal Chemistry 57 (2014): 2033–2046, 10.1021/jm401499g. 24320933

[15] D. G. Brown , P. R. Bernstein , A. Griffin , et al., “Discovery of Spirofused Piperazine and Diazepane Amides as Selective Histamine‐3 Antagonists With in Vivo Efficacy in a Mouse Model of Cognition,” Journal of Medicinal Chemistry 57 (2014): 733–758, 10.1021/jm4014828. 24410637

[16] Literature search was performed using the Reaxys database on November 30, 2025.

[17] H. Wang , Y. Li , L. Huang , et al., “Synthesis of Spirocyclopropane Skeletons Enabled by Rh(III)‐Catalyzed Enantioselective C−H Activation/[4+2] Annulation,” Chem Catalysis 3 (2023): 100822–100822, 10.1016/j.checat.2023.100822.

[18] W. Yu , J. Kozlowski , D. J. Clausen , et al., “Inhibitors of Histone Deacetylase Useful for the Treatment or Prevention of HIV Infection,” WO/2020/096916 A2 (2020).

[19] C. Ebner and E. M. Carreira , “Cyclopropanation Strategies in Recent Total Syntheses,” Chemical Reviews 117 (2017): 11651–11679, 10.1021/acs.chemrev.6b00798. 28467054

[20] L. Zhang , B. M. De Muynck , A. N. Paneque , J. E. Rutherford , and D. A. Nagib , “Carbene Reactivity From Alkyl and Aryl Aldehydes,” Science 377 (2022): 649–654, 10.1126/science.abo6443. 35926031 PMC9439075

[21] Q. Sun , J.‐N. Belting , J. Hauda , et al., “Spiro‐C(sp^3^ )‐Atom Transfer: Creating Rigid Three‐Dimensional Structures with Ph_2_SCN_2_ ,” Science 387 (2025): 885–892, 10.1126/science.ads5974. 39977512

[22] Z. Wang , A. G. Herraiz , A. M. del Hoyo , and M. G. Suero , “Generating Carbyne Equivalents ith Photoredox Catalysis,” Nature 554 (2018): 86–91, 10.1038/nature25185. 29388953

[23] K. Hou , W. Huang , M. Qi , et al., “De Novo Design of Porphyrin‐Containing Proteins as Efficient and Stereoselective Catalysts,” Science 388 (2025): 665–670, 10.1126/science.adt7268. 40339022 PMC12316402

[24] J. Pan , J.‐H. Wu , H. Zhang , et al., “Highly Enantioselective Synthesis of Fused Tri‐ and Tetrasubstituted Aziridines: aza‐Darzens Reaction of Cyclic Imines With α‐Halogenated Ketones Catalyzed by Bifunctional Phosphonium Salt,” Angewandte Chemie International Edition 58 (2019): 7425–7430, 10.1002/anie.201900613. 30958634

[25] D. Lu , X. Liu , J.‐H. Wu , et al., “Asymmetric Construction of Bispiro‐Cyclopropane‐Pyrazolones via a [2+1] Cyclization Reaction by Dipeptide‐Based Phosphonium Salt Catalysis,” Advanced Synthesis & Catalysis 362 (2020): 1966–1971, 10.1002/adsc.202000073.

[26] X. Yu , F. Wang , J. Du , J.‐P. Tan , J. Pan , L. Zhu , and T. Wang , “Asymmetric Synthesis of Bis‐Spiro Cyclopropane Skeletons Via Bifunctional Phosphonium Salt‐Catalyzed [2 + 1] Annulation,” Organic Chemistry Frontiers 11 (2024): 6666–6671, 10.1039/D4QO01424C.

[27] M. Mendel , T. M. Karl , J. Hamm , et al., “Dynamic Stereomutation of Vinylcyclopropanes With Metalloradicals,” Nature 631 (2024): 80–86, 10.1038/s41586-024-07555-1. 38898284 PMC11222138

[28] R. K. Raut , S. Matsutani , F. Shi , et al., “Catalytic Asymmetric Fragmentation of Cyclopropanes,” Science 386 (2024): 225–230, 10.1126/science.adp9061. 39388547

[29] Y. Peng , X. Huo , Y. Luo , L. Wu , and W. Zhang , “Enantio‐ and Diastereodivergent Synthesis of Spirocycles Through Dual‐Metal‐Catalyzed [3+2] Annulation of 2‐Vinyloxiranes With Nucleophilic Dipoles,” Angewandte Chemie International Edition 60 (2021): 24941–24949, 10.1002/anie.202111842. 34532948

[30] O. Viudes , C. Besnard , A. F. Siegle , et al., “All‐Heteroatom‐Substituted Carbon Spiro Stereocenters: Synthesis, Resolution, Enantiomeric Stability, and Absolute Configuration,” Journal of the American Chemical Society 147 (2025): 21121–21130, 10.1021/jacs.5c06394. 40464057 PMC12200235

[31] L. Zhang , C. Peng , Y. Shi , et al., “Asymmetric Synthesis of NOSPIN Derivatives,” Angewandte Chemie International Edition 65 (2026): e5527700, 10.1002/anie.5527700. 41981974

[32] Z. Liu , P. Sivaguru , G. Zanoni , and X. Bi , “ *N* ‐Triftosylhydrazones: A New Chapter for Diazo‐Based Carbene Chemistry,” Accounts of Chemical Research 55 (2022): 1763–1781, 10.1021/acs.accounts.2c00186. 35675648

[33] P. Sivaguru , Y. Pan , N. Wang , and X. Bi , “Who is Who in the Carbene Chemistry of *N* ‐Sulfonyl Hydrazones,” Chinese Journal of Chemistry 42 (2024): 2071–2108, 10.1002/cjoc.202300716.

[34] X. Zhang , P. Sivaguru , Y. Pan , N. Wang , W. Zhang , and X. Bi , “The Carbene Chemistry of *N* ‐Sulfonyl Hydrazones: The Past, Present, and Future,” Chemical Reviews 125 (2025): 1049–1190, 10.1021/acs.chemrev.4c00742. 39792453

[35] J. R. Denton , D. Sukumaran , and H. M. L. Davies , “Enantioselective Synthesis of Trifluoromethyl‐Substituted Cyclopropanes,” Organic Letters 9 (2007): 2625–2628, 10.1021/ol070714f. 17552531

[36] X. Zhang , C. Tian , Z. Wang , P. Sivaguru , S. P. Nolan , and X. Bi , “Fluoroalkyl N‐Triftosylhydrazones as Easily Decomposable Diazo Surrogates for Asymmetric [2+1] Cycloaddition: Synthesis of Chiral Fluoroalkyl Cyclopropenes and Cyclopropanes,” ACS Catalysis 11 (2021): 8527–8537, 10.1021/acscatal.1c01483.

[37] K. Müller , C. Faeh , and F. Diederich , “Fluorine in Pharmaceuticals: Looking Beyond Intuition,” Science 317 (2007): 1881–1886, 10.1126/science.1131943. 17901324

[38] Y.‐J. Yu , F.‐L. Zhang , T.‐Y. Peng , et al., “Sequential C–F Bond Functionalizations of Trifluoroacetamides and Acetates Via Spin‐Center Shifts,” Science 371 (2021): 1232–1240, 10.1126/science.abg0781. 33674411

[39] Y. Luo , Y. Li , J. Wu , X.‐S. Xue , J. F. Hartwig , and Q. Shen , “Oxidative Addition of an Alkyl Halide To Form a Stable Cu(III) Product,” Science 381 (2023): 1072–1079, 10.1126/science.adg9232. 37676952 PMC10658983

[40] S. Purser , P. R. Moore , S. Swallow , and V. Gouverneur , “Fluorine in Medicinal Chemistry,” Chemical Society Reviews 37 (2008): 320–330, 10.1039/B610213C. 18197348

[41] A. Pons , L. Delion , T. Poisson , A. B. Charette , and P. Jubault , “Asymmetric Synthesis of Fluoro, Fluoromethyl, Difluoromethyl, and Trifluoromethylcyclopropanes,” Accounts of Chemical Research 54 (2021): 2969–2990, 10.1021/acs.accounts.1c00261. 34232626

[42] J. Xu , B. J. Bloomer , J. N. Brunn , et al., “Enantioselective Synthesis of Spirocyclic Nitrogen‐Containing Heterocycles Catalyzed by an Iridium‐Containing Cytochrome,” Journal of the American Chemical Society 147 (2025): 28875–28881, 10.1021/jacs.5c06239. 40749167 PMC12356651

[43] J. L. Kennemur , Y. Long , C. J. Ko , A. Das , and F. H. Arnold , “Enzymatic Stereodivergent Synthesis of Azaspiro[2.y]alkanes,” Journal of the American Chemical Society 147 (2025): 27165–27171, 10.1021/jacs.5c07015. 40694486 PMC12951711

[44] J. K. Sailer , D. Ly , A. Wang , D. G. Musaev , and H. M. L. Davies , “Enantioselective and Diastereoselective Synthesis of Azaspiro[n.2]Alkanes by Rhodium‐Catalyzed Cyclopropanations,” ACS Catalysis 15 (2025): 15253–15260, 10.1021/acscatal.5c04199. 40933345 PMC12418304

[45] T. Saito , A. Navarro , and H. M. L. Davies , “Enantioselective Synthesis of Spirolactones and Spirolactams Under Low Catalyst Loadings,” Journal of the American Chemical Society 148 (2026): 11352–11358, 10.1021/jacs.6c01407. 41785448 PMC13003446

[46] K. Liao , S. Negretti , D. G. Musaev , J. Bacsa , and H. M. L. Davies , “Site‐Selective and Stereoselective Functionalization of Unactivated C–H Bonds,” Nature 533 (2016): 230–234, 10.1038/nature17651. 27172046

[47] K. Liao , T. C. Pickel , V. Boyarskikh , J. Bacsa , D. G. Musaev , and H. M. L. Davies , “Site‐Selective and Stereoselective Functionalization of Non‐Activated Tertiary C–H Bonds,” Nature 551 (2017): 609–613, 10.1038/nature24641. 29156454

[48] S. Dutta , C. G. Daniliuc , C. Mück‐Lichtenfeld , and A. Studer , “Enantioselective Synthesis of Tetrahydro‐1H‐1,3‐Methanocarbazoles by Formal (3 + 3)‐Cycloaddition Using Bicyclo[1.1.0]Butanes,” Journal of the American Chemical Society 147 (2025): 4249–4257, 10.1021/jacs.4c14276. 39871803

[49] J.‐J. Wang , L. Tang , Y. Xiao , W.‐B. Wu , G. Wang , and J.‐J. Feng , “Switching Between the [2π+2σ] and Hetero‐[4π+2σ] Cycloaddition Reactivity of Bicyclobutanes With Lewis Acid Catalysts Enables the Synthesis of Spirocycles and Bridged Heterocycles,” Angewandte Chemie International Edition 63 (2024): e202405222, 10.1002/anie.202405222. 38729920

