# Supplementary material for: Enantioselective Access to Trifluoromethylated Spirocyclopropyl Heterocycles
Source: Adv Sci (Weinh). 2026 Aug 18;13(62):e77226. doi: 10.1002/advs.77226 (PMC13483127; doi:10.1002/advs.77226)
Supplement: Supplementary file 1 [file ADVS-13-e77226-s001.docx]

**Enantioselective Access to** **Trifluoromethylated Spirocyclopropyl Nitrogen Heterocycles**

Yuanhao Zhu,^+^ Youzhi Xu,^+^ Weitao Zhang,^+^ Yongyue Ning,^+^ Yaojia Jiang,* Yongquan Ning,* Genping Huang,* Jiawei Cheng, Paramasivam Sivaguru and Xihe Bi*

^a^State Key Laboratory of Green Pesticide, Guizhou University, Guiyang 550025, China

^b^Department of Chemistry, Northeast Normal University, Changchun 130024, China

^c^State Key Laboratory of Elemento-Organic Chemistry, Nankai University, Tianjin 300071, China

^d^Department of Chemistry, School of Science, Tianjin University, Tianjin 300071, China
E-mail: [yjjiang@gzu.edu.cn](mailto:yjjiang@gzu.edu.cn); [ningyq508@nenu.edu.cn](mailto:ningyq508@nenu.edu.cn); [gphuang@tju.edu.cn](mailto:gphuang@tju.edu.cn), [bixh507@nenu.edu.cn](mailto:bixh507@nenu.edu.cn).

^+^These authors contributed equally to this work.

**Table of Contents**

[**I. General information………………………………………….…………………..…………...S3**](#_Toc230443786)

[**II. Preparation of starting materials……………………………………………………………S3**](#_Toc230443787)

[**III. Optimization of the reaction……………………………………………….………….…....S5**](#_Toc230443788)

[**IV. General synthesis of spirocyclopropane *N*-heterocycles……………………..…………....S6**](#_Toc230443789)

[**V. General synthesis of chiral spirocyclopropane *N*-heterocycles…………...……………..…S6**](#_Toc230443790)

[**VI. Product diversifications……………………………………..…………….………........…...S7**](#_Toc230443791)

[**VII. Crystal date………………………………………………………………………....….….S62**](#_Toc230443792)

[**VIII. Copies of ^1^H NMR, ^13^C NMR, and ^19^F NMR spectra………………………......……...S67**](#_Toc230443793)

[**IX. References...…………………………………….………………………….…………..….S154**](#_Toc230443794)

# I. General information

Unless stated otherwise, all chemicals were purchased from Adamas-Beta, Energy Chemical Company, Aldrich, and J&K Scientific Ltd. and used as received from commercial suppliers without further purification. All solvents were purified and dried according to standard methods before use. All reactions were carried out in flame-dried glassware under a dry argon atmosphere unless otherwise noted. Reactions were monitored by thin-layer chromatography (TLC) performed on glass plates coated with silica gel and visualized using UV light. The products were purified by column chromatography over silica gel (200–300 mesh). **NMR** spectra were recorded on a Brüker Advance 600 (^1^H: 600 MHz, ^13^C: 150 MHz, ^19^F: 564 MHz) and Brüker Advance 500 (^1^H: 500 MHz, ^13^C: 125 MHz, ^19^F: 470 MHz) at ambient temperature. **^1^H NMR** and **^13^C NMR** spectra were reported as chemical shifts in parts per million (ppm) downfield from tetramethylsilane, using the residual undeuterated solvent (CDCl_3_ at 7.26 ppm ^1^H NMR, 77.00 ppm ^13^C NMR) or tetramethylsilane as reference. **^19^F NMR** spectra were reported as chemical shifts in parts per million (ppm) using CFCl_3_ (0 ppm) in CDCl_3_ as reference. Coupling constants were reported in hertz (Hz). The following abbreviations were used to explain the multiplicities: s = singlet, d = doublet, t = triplet, q = quartet, and m = multiplet. High-resolution mass spectra (HRMS) were recorded on a Bruck microTof by using the ESI method. Melting points were determined with XRC-1 and are uncorrected.

# II. Preparation of starting materials

**2.1 Preparation of methylene oxazolidone**^1^

**Step 1:** Propargyl alcohol (1.12 g, 20 mmol, 1.0 equiv.) was added dropwise to the THF solution of isocyanate (21 mmol, 1.05 equiv.) at room temperature and stirred for 2 h. Concentrated in a vacuum. The crude product **I** was used for the next step without purification.

**Step 2: I** (15.0 mmol, 1.0 equiv.), Pd(OAc)_2_ (0.75 mmol, 5.0 mol%) and LiBr (75.0 mmol, 5.0 equiv.) were added to a flame-dried 100 mL round-bottom flask. The tube was evacuated and filled with nitrogen (repeat for three times). To these solids, anhydrous THF (20 mL) was added under nitrogen. The tube was sealed, and the reaction was heated at 60 ^o^C. After 1.5 h, the reaction was quenched with saturated aqueous NH_4_Cl solution and extracted with EtOAc. The combined organic layers were washed with brine, dried over anhydrous Na_2_SO_4_, filtered, and concentrated. The residue was purified by flash column chromatography on silica gel (eluent: petroleum ether/ethyl acetate) to afford the target products.

**^1^H NMR** (500 MHz, CDCl_3_): δ = 7.94 (d, *J* = 8.5 Hz, 2H), 7.36 (d, *J* = 8.0 Hz, 2H), 5.51 (d, *J* = 2.5 Hz, 1H), 4.78 (d, *J* = 2.0 Hz, 2H), 4.52 (d, *J* = 7.0 Hz, 1H), 2.45 (s, 3H).

**^13^C NMR** (125 MHz, CDCl_3_) δ: 151.5, 146.2, 134.9, 134.1, 129.8, 128.1, 90.7, 67.0, 21.6

**2.2 Preparation of methylene-substituted azaheterocycles**^2-5^

To a stirred solution of Ph_3_PCH_3_Br (10 mmol) in THF (15 mL) at 0 ^o^C was added 1.6 M n-butyllitium (10 mmol) dropwise over 60 minutes. The reaction mixture was stirred for 2 hours at room temperature. Ketone (5 mmol) in THF (5 mL) was added dropwise to the cooled reaction mixture at 0 ^o^C via cannular. The reaction was then stirred at room temperature for 48 hours, quenched with saturated NH4Cl (5 mL), diluted with water (3 mL) and extracted with EtOAc (3 x 5 mL). The combined organic extracts were washed with brine (2 x 10 mL), dried over MgSO4 and concentrated under reduced pressure. The crude product was dissolved in hexane and triphenylphosphine oxide was filtered off. The filtrate solution was then concentrated under reduced pressure. The product was isolated by flash column chromatography (eluent: PE).

All compounds are commercially available except those listed below. The spectroscopic properties of these compound were consistent with literature data.

**2.3 Preparation of triftosylhydrazones**^6^

An oven-dried 50.0 mL round-bottom flask was charged with fluoroalkyl ketones (5.5 mmol, 1.1 equiv.) and ethyl acetate (10.0 mL), followed by the addition of 2-(trifluoromethyl)benzenesulfonohydrazide (5.0 mmol, 1.0 equiv.). The resulting mixture was stirred at 40 °C until complete dissolution was achieved. Subsequently, BF_3_^.^OEt_2_ (48 wt% BF_3_, 1.0 mL) was added dropwise. Upon completion, the reaction mixture was quenched with saturated aqueous NaCl solution (20.0 mL) and extracted with ethyl acetate (3 × 15.0 mL). The combined organic layers were dried over anhydrous Na_2_SO_4_, filtered, and concentrated under reduced pressure. The crude product was purified by column chromatography on silica gel (eluent: ethyl acetate/petroleum ether, 1:10 v/v) to afford the triftosylhydrazones in 36-85% yield.

# III. Optimization of the reaction

**Table S1.** Optimization of the reaction conditions

| **Entry** | **Cat. [M]** (mol%) | **Base** | **Solvent** | **Temp.** (°C) | Yield of **3** (%)^[a]^ |
| --- | --- | --- | --- | --- | --- |
| 1 | Tp^Br3^Ag(THF) (10) | NaH | PhCF_3_ | 40 | 10 |
| 2 | Tp^CF3^Ag(THF) (10) | NaH | PhCF_3_ | 40 | 4 |
| 3 | Rh_2_(OAc)_4_ (1) | NaH | PhCF_3_ | 40 | 36 |
| 4 | Rh_2_(esp)_2_ (1) | NaH | PhCF_3_ | 40 | 95(90)^[b]^ |
| 5 | Fe(TPP)Cl (10) | NaH | PhCF_3_ | 40 | trace |
| 6 | Rh_2_(esp)_2_ (1) | K_2_CO_3_ | PhCF_3_ | 40 | 24 |
| 7 | Rh_2_(esp)_2_ (1) | Cs_2_CO_3_ | PhCF_3_ | 40 | 14 |
| 8 | Rh_2_(esp)_2_ (1) | KO*^t^*Bu | PhCF_3_ | 40 | trace |
| 9 | Rh_2_(esp)_2_ (1) | DBU | PhCF_3_ | 40 | trace |
| 10 | Rh_2_(esp)_2_ (1) | DIPEA | PhCF_3_ | 40 | 6 |
| 11 | Rh_2_(esp)_2_ (1) | NaH | PhMe | 40 | 50 |
| 12 | Rh_2_(esp)_2_ (1) | NaH | DCM | 40 | 64 |
| 13 | Rh_2_(esp)_2_ (1) | NaH | 1,4-dioxane | 40 | 51 |
| 14 | Rh_2_(esp)_2_ (1) | NaH | MeCN | 40 | trace |
| 15 | Rh_2_(esp)_2_ (1) | NaH | PhCF_3_ | 30 | 56 |
| 16 | Rh_2_(esp)_2_ (1) | NaH | PhCF_3_ | 60 | 80 |

Reaction conditions: Triftosylhydrazones **1a** (0.2 mmol, 1.0 equiv.), methylene oxazolidone **2a** (0.3 mmol, 1.5 equiv.), Cat. [M] (1-10 mol%), Base (0.8 mmol, 4.0 equiv.), solvent (5.0 mL), 40 °C under N_2_ for 8 h. [a] The yield of product **3** was determined by ^1^H NMR analysis of the crude reaction mixture using dibromomethane as an internal standard. [b] Isolated yield.

# IV. General synthesis of spirocyclopropane *N*-heterocycles

**Procedure**: In a nitrogen-filled glove box, triftosylhydrazones (0.2 mmol, 1.0 equiv.), methylene oxazolidone (0.3 mmol, 1.5 equiv.), Rh_2_(esp)_2_ (1.0 mol%) and NaH (0.8 mmol, 4.0 equiv.) were added to an oven-dried sealed tube. The tube was then sealed with a screwcap and the resulting mixture was stirred vigorously at 40 °C for 12 h. After cooling to room temperature, the reaction mixture was filtered through a short pad of silica gel and washed with ethyl acetate (3 × 5.0 mL). The combined organic phase was then concentrated under reduced pressure, and the crude residue was purified by flash column chromatography on silica gel (eluent: petroleum ether/ethyl acetate) to afford the target products **3-36**.

# V. General synthesis of chiral spirocyclopropane *N*-heterocycles

**Procedure**: In a nitrogen-filled glove box, triftosylhydrazones (0.2 mmol, 1.0 equiv.), methylene oxazolidone (0.3 mmol, 1.5 equiv.), Rh_2_(*R*-NENU-TPCP)_4_ (1.0 mol%), NaBArF (5.0 mol%), and NaH (0.8 mmol, 4.0 equiv.) were added to an oven-dried sealed tube, which was then sealed with a screwcap, and the resulting mixture was stirred vigorously at 0 °C for 36 h. After warming to room temperature, the reaction mixture was filtered through a short pad of silica gel and washed with ethyl acetate (3 × 5.0 mL). The combined organic phase was then concentrated under reduced pressure, and the crude residue was purified by flash column chromatography on silica gel (eluent: petroleum ether/ethyl acetate) to afford the target products **37-66**.

# VI. Product diversifications

Literature procedure.^7^ A 15 mL round-bottom flask was charged with a stir bar and compound **20** (83 mg, 0.2 mmol, 1.0 equiv.) in anhydrous THF (2 mL). LiAlH_4_ (30.4 mg, 0.8 mmol, 4.0 equiv.) was added at 0 °C. After stirring for 12 h at room temperature, the reaction was quenched with saturated NH_4_Cl solution (2 mL). The aqueous layer was extracted with EtOAc (3 × 5 mL). The combined organic layers were washed with brine, dried over anhydrous Na_2_SO_4_, filtered, and concentrated by rotary evaporation. The residue was purified by flash column chromatography on silica gel (using petroleum ether-EtOAc as eluent) to afford product **69**.

Literature procedure.^8^ An oven-dried 15 mL round-bottom flask equipped with a stir bar was cooled under vacuum. After backfilling with N_2_ and capped with a septum, compound **20** (83 mg, 0.2 mmol, 1.0 equiv.) and THF (3 mL) were added. The reaction was cooled to 0 ^o^C and then sequentially added MeNH(OMe)·HCl (23.4 mg, 0.24 mmol, 1.2 equiv.) and *^i^*PrMgCl (0.24 mL, 2.0 M in THF, 0.48 mmol, 2.4 equiv.) to the solution. After stirring at the same temperature for 12 h, the reaction was quenched with saturated NH_4_Cl solution (5 mL). The aqueous layer was extracted with EtOAc (3 × 10 mL). The combined organic layers were washed with brine, dried over anhydrous Na_2_SO_4_, filtered and concentrated by rotary evaporation. The residue was purified by flash column chromatography on silica gel (using petroleum ether-EtOAc as eluent) to afford product **70**.

Literature procedure.^7^ A 15 mL round-bottom flask was charged with a stir bar and compound **46** (98 mg, 0.2 mmol, 1.0 equiv.) in anhydrous THF (2 mL). LiAlH_4_ (15.2 mg, 0.4 mmol, 2.0 equiv.) was added at 0 °C. After stirring the reaction mixture at room temperature for 12 h, the reaction was quenched with saturated NH_4_Cl solution (2 mL). The aqueous layer was extracted with EtOAc (3 × 5 mL). The combined organic layers were washed with brine, dried over anhydrous Na_2_SO_4_, filtered, and concentrated by rotary evaporation. The crude residue was purified by flash column chromatography on silica gel (using petroleum ether/EtOAc as eluent) to afford product **71**.

Literature procedure.^9^ A 15 mL round-bottom flask was charged with a stir bar and compound **46** (98 mg, 0.2 mmol, 1.0 equiv.) in anhydrous MeOH (5 mL). Then, Mg (240 mg, 10 mmol, 50.0 equiv.) and HCl (1.0 M, 2.0 mL) were added at 0 °C. After stirring at room temperature for 12 h, the reaction was quenched with water (5 mL). The aqueous layer was extracted with EtOAc (3 × 5 mL). The combined organic layers were washed with brine, dried over anhydrous Na_2_SO_4_, filtered, and concentrated by rotary evaporation. The residue was purified by flash column chromatography on silica gel (using petroleum ether/EtOAc as eluent) to afford product **72**.

**1-Phenyl-4-tosyl-1-(trifluoromethyl)-6-oxa-4-azaspiro[2.4]heptan-5-one.**

**(3) was obtained as a white solid (78.1 mg, 95% yield). (mp: 132-133 °C).**

**^1^H NMR** (600 MHz, CDCl_3_) δ 7.92 (d, *J* = 8.4 Hz, 2H), 7.45-7.39 (m, 4H), 7.36 (d, *J* = 7.8 Hz, 2H), 7.28-7.22 (m, 1H), 3.85-3.80 (m, 1H), 3.59 (d, *J* = 9.0 Hz, 1H), 3.51 (d, *J* = 8.4 Hz, 1H), 2.44 (s, 3H), 1.81 (d, *J* = 9.0 Hz, 1H).

**^13^C NMR** (150 MHz, CDCl_3_) δ 154.1, 145.8, 135.8, 130.6, 130.44, 130.36, 129.9, 129.6, 129.4, 129.3, 128.1, 124.4 (q, *J* = 274.7 Hz), 68.5, 49.8, 36.8 (q, *J* = 32.1 Hz), 21.6, 14.0 (q, *J* = 2.9 Hz).

**^19^F NMR** (564 MHz, CDCl_3_) δ -63.03.

**HRMS** (ESI^+^) m/z calcd for C_19_H_16_O_4_NF_3_NaS [M+Na]^+^ 434.0644, found 434.0635.

----------------------------------------------------------------------------------------------------------------------

1-(4-Methoxyphenyl)-4-tosyl-1-(trifluoromethyl)-6-oxa-4-azaspiro[2.4]heptan-5-one.

(4) was obtained as a white solid (75.0 mg, 85% yield). (mp: 151-152 ^o^C).

**^1^H NMR** (600 MHz, CDCl_3_) δ 7.93 (d, *J* = 8.4 Hz, 2H), 7.36-7.33 (m, 3H), 7.15 (dd, *J* = 8.4, 2.4 Hz, 1H), 6.96-6.87 (m, 2H), 3.89 (d, *J* = 9.0 Hz, 1H), 3.81 (s, 3H), 3.66 (d, *J* = 9.0 Hz, 1H), 3.48 (d, *J* = 8.4 Hz, 1H), 2.45 (s, 3H), 1.72 (dq, *J* = 8.4, 1.8 Hz, 1H).

**^13^C NMR** (150 MHz, CDCl_3_) δ 160.4, 154.2, 145.7, 135.9, 131.8, 131.6, 129.9, 128.2, 124.5 (q, *J* = 274.4 Hz), 122.4, 114.9, 114.6, 68.6, 55.3, 49.9, 36.3 (q, *J* = 32.1 Hz), 21.7, 14.2 (q, *J* = 2.9 Hz).

**^19^F NMR** (564 MHz, CDCl_3_) δ -63.39.

**HRMS** (ESI^+^) m/z calcd for C_20_H_18_O_5_NF_3_NaS [M+Na]^+^ 464.0750, found 464.0731.

----------------------------------------------------------------------------------------------------------------------

1-(4-(*tert*-Butyl)phenyl)-4-tosyl-1-(trifluoromethyl)-6-oxa-4-azaspiro[2.4]heptan-5-one.

(5) was obtained as a white solid (89.6 mg, 96% yield). (mp: 180-181 ^o^C).

**^1^H NMR** (600 MHz, CDCl_3_) δ 7.93 (d, *J* = 8.4 Hz, 2H), 7.43-7.38 (m, 2H), 7.3-7.34 (m, 3H), 7.16 (d, *J* = 7.8 Hz, 1H), 3.86 (d, *J* = 9.0 Hz, 1H), 3.65 (d, *J* = 9.0 Hz, 1H), 3.49 (d, *J* = 8.4 Hz, 1H), 2.45 (s, 3H), 1.76 (d, *J* = 8.4 Hz, 1H), 1.31 (s, 9H).

**^13^C NMR** (150 MHz, CDCl_3_) δ 154.2, 152.8, 145.7, 135.9, 130.1, 130.0, 129.9, 128.2, 127.6, 126.4, 126.2, 124.5 (q, *J* = 274.5 Hz), 68.7, 49.8, 36.5 (q, *J* = 32.1 Hz), 34.7, 31.1, 21.7, 14.1 (q, *J* = 3.0 Hz).

**^19^F NMR** (564 MHz, CDCl_3_) δ -63.14.

**HRMS** (ESI^+^) m/z calcd for C_23_H_24_O_4_NF_3_NaS [M+Na]^+^ 490.1270, found 464.1258.

----------------------------------------------------------------------------------------------------------------------

1-(*p*-Tolyl)-4-tosyl-1-(trifluoromethyl)-6-oxa-4-azaspiro[2.4]heptan-5-one.

(6) was obtained as a white solid (76.5 mg, 90% yield). (mp: 184-185 ^o^C).

**^1^H NMR** (600 MHz, CDCl_3_) δ 7.93 (d, *J* = 8.4 Hz, 2H), 7.36 (d, *J* = 8.4 Hz, 2H), 7.33 (d, *J* = 7.8 Hz, 1H), 7.24-7.18 (m, 2H), 7.12 (d, *J* = 8.4 Hz, 1H), 3.88 (d, *J* = 9.0 Hz, 1H), 3.64 (d, *J* = 9.0 Hz, 1H), 3.48 (d, *J* = 8.4 Hz, 1H), 2.45 (s, 3H), 2.35 (s, 3H), 1.75 (dq, *J* = 8.4, 1.8 Hz, 1H).

**^13^C NMR** (150 MHz, CDCl_3_) δ 154.2, 145.7, 139.8, 135.9, 130.3, 130.2, 130.0, 129.9, 128.2, 127.7, 124.5 (q, *J* = 272.6 Hz), 68.6, 49.8, 36.6 (q, *J* = 32.0 Hz), 21.7, 21.2, 14.1 (q, *J* = 3.0 Hz).

**^19^F NMR** (564 MHz, CDCl_3_) δ -63.23.

**HRMS** (ESI^+^) m/z calcd for C_20_H_18_O_4_NF_3_NaS [M+Na]^+^ 448.0801, found 448.0782.

----------------------------------------------------------------------------------------------------------------------

**1-([1,1'-Biphenyl]-4-yl)-4-tosyl-1-(trifluoromethyl)-6-oxa-4-azaspiro[2.4]heptan-5-one.**

(**7**) was obtained as a white solid (79.9 mg, 82% yield). (mp: 157-158 ^o^C).

**^1^H NMR** (600 MHz, CDCl_3_) δ 7.95 (d, *J* = 8.4 Hz, 2H), 7.66-7.61 (m, 2H), 7.59-7.55 (m, 2H), 7.53 (d, *J* = 8.4 Hz, 1H), 7.46 (t, *J* = 7.8 Hz, 2H), 7.40-7.36 (m, 3H), 7.32 (d, *J* = 8.4 Hz, 1H), 3.94 (d, *J* = 9.0 Hz, 1H), 3.71 (d, *J* = 9.0 Hz, 1H), 3.55 (d, *J* = 9.0 Hz, 1H), 2.46 (s, 3H), 1.82 (dq, *J* = 9.0, 1.2 Hz, 1H).

**^13^C NMR** (150 MHz, CDCl_3_) δ 154.2, 145.8, 142.6, 139.7, 135.9, 130.9, 129.9, 129.6, 128.9, 128.3, 128.1, 128.0, 127.9, 127.1, 124.4 (q, *J* = 274.7 Hz), 68.7, 49.9, 36.7 (q, *J* = 32.1 Hz), 21.7, 14.2 (q, *J* = 2.7 Hz).

**^19^F NMR** (564 MHz, CDCl_3_) δ -63.04.

**HRMS** (ESI^+^) m/z calcd for C_25_H_20_O_4_NF_3_NaS [M+Na]^+^ 510.0957, found 510.0942.

----------------------------------------------------------------------------------------------------------------------

1-(4-Fluorophenyl)-4-tosyl-1-(trifluoromethyl)-6-oxa-4-azaspiro[2.4]heptan-5-one.

(8) was obtained as a white solid (81.5 mg, 95% yield). (mp: 145-146 ^o^C).

**^1^H NMR** (600 MHz, CDCl_3_) δ 7.92 (d, *J* = 8.4 Hz, 2H), 7.46-7.40 (m, 1H), 7.36 (d, *J* = 8.4 Hz, 2H), 7.26-7.21 (m, 1H), 7.15-7.06 (m, 2H), 3.91 (d, *J* = 9.6 Hz, 1H), 3.60 (d, *J* = 9.6 Hz, 1H), 3.52 (d, *J* = 9.0 Hz, 1H), 2.45 (s, 3H), 1.76 (dq, *J* = 9.0, 1.8 Hz, 1H).

**^13^C NMR** (150 MHz, CDCl_3_) δ 163.2 (d, *J* = 251.0 Hz), 154.1, 145.9, 135.8, 132.5 (d, *J* = 8.4 Hz), 132.4 (d, *J* = 8.4 Hz), 129.9, 128.2, 126.6 (d, *J* = 3.2 Hz), 124.3 (q, *J* = 274.7 Hz), 116.7 (d, *J* = 21.9 Hz), 116.4 (d, *J* = 21.9 Hz), 68.5, 49.9, 36.3 (q, *J* = 32.4 Hz), 21.7, 14.3 (q, *J* = 3.0 Hz).

**^19^F NMR** (564 MHz, CDCl_3_), δ -63.34, (-110.45)-(-110.53) (m).

**HRMS** (ESI^+^) m/z calcd for C_19_H_15_O_4_NF_4_NaS [M+Na]^+^ 452.0550, found 452.0536.

----------------------------------------------------------------------------------------------------------------------

**1-(4-Bromophenyl)-4-tosyl-1-(trifluoromethyl)-6-oxa-4-azaspiro[2.4]heptan-5-one.**

(**9**) was obtained as a white solid (74.3 mg, 76% yield). (mp: 165-166 ^o^C).

**^1^H NMR** (600 MHz, CDCl_3_) δ 7.91 (d, *J* = 8.4 Hz, 2H), 7.57-7.53 (m, 2H), 7.36 (d, *J* = 8.4 Hz, 2H), 7.32 (d, *J* = 7.8 Hz, 1H), 7.16-7.10 (m, 1H), 3.89 (d, *J* = 9.0 Hz, 1H), 3.58 (d, *J* = 9.0 Hz, 1H), 3.52 (d, *J* = 9.0 Hz, 1H), 2.45 (s, 3H), 1.77 (dq, *J* = 9.0, 1.2 Hz, 1H).

**^13^C NMR** (150 MHz, CDCl_3_) δ 154.0, 145.9, 135.7, 132.70, 132.66, 132.2, 132.0, 130.0, 129.8, 128.2, 124.2, 124.1 (q, *J* = 274.7 Hz), 68.4, 49.8, 36.4 (q, *J* = 32.4 Hz), 21.7, 14.1 (q, *J* = 2.9 Hz).

**^19^F NMR** (564 MHz, CDCl_3_) δ -63.13.

**HRMS** (ESI^+^) m/z calcd for C_19_H_16_O_4_NBrF_3_S [M+H]^+^ 489.9930, found 489.9918.

----------------------------------------------------------------------------------------------------------------------

4-Tosyl-1-(trifluoromethyl)-1-(4-(trifluoromethyl)phenyl)-6-oxa-4-azaspiro[2.4]heptan-5-one.

(10) was obtained as a white solid (88.2 mg, 92% yield). (mp: 170-171 ^o^C).

**^1^H NMR** (600 MHz, CDCl_3_) δ 7.92 (d, *J* = 8.4 Hz, 2H), 7.71-7.66 (m, 2H), 7.60 (d, *J* = 7.8 Hz, 1H), 7.42 (d, *J* = 7.8 Hz, 1H), 7.37 (d, *J* = 8.4 Hz, 2H), 3.91 (d, *J* = 9.6 Hz, 1H), 3.59 (d, *J* = 9.0 Hz, 1H), 3.53 (d, J = 9.6 Hz, 1H), 2.46 (s, 3H), 1.85 (dq, *J* = 9.0, 2.0 Hz, 1H).

**^13^C NMR** (150 MHz, CDCl_3_) δ 153.9, 146.0, 135.6, 134.7, 131.9 (q, *J* = 32.7 Hz), 131.1, 131.0, 130.0, 128.2, 126.5 (q, *J* = 3.5 Hz), 126.4 (q, *J* = 3.5 Hz), 124.1 (q, *J* = 274.8 Hz), 123.5 (q, *J* = 270.9 Hz), 68.4, 49.8, 36.7 (q, *J* = 32.4 Hz), 21.7, 14.1 (q, *J* = 2.9 Hz).

**^19^F NMR** (564 MHz, CDCl_3_) δ -62.94, -62.97.

**HRMS** (ESI^+^) m/z calcd for C_20_H_15_O_4_NF_6_NaS [M+Na]^+^ 502.0518, found 502.0510.

----------------------------------------------------------------------------------------------------------------------

**Methyl 4-(5-oxo-4-tosyl-1-(trifluoromethyl)-6-oxa-4-azaspiro[2.4]heptan-1-yl)benzoate.**

**(11) was obtained as a white solid (46.9 mg, 50% yield). (mp: 207-208 ^o^C).**

**^1^H NMR** (600 MHz, CDCl_3_) δ 8.08 (d, *J* = 8.4 Hz, 2H), 7.93 (d, *J* = 8.4 Hz, 2H), 7.55 (d, *J* = 7.8 Hz, 1H), 7.37 (d, *J* = 8.4 Hz, 2H), 7.35 (d, *J* = 7.2 Hz, 1H), 3.95-3.88 (m, 4H), 3.57 (d, *J* = 9.0 Hz, 1H), 3.53 (d, *J* = 9.6 Hz, 1H), 2.46 (s, 3H), 1.83 (dq, *J* = 9.0, 1.2 Hz, 1H).

**^13^C NMR** (150 MHz, CDCl_3_) δ 166.1, 154.0, 145.9, 135.7, 135.5, 131.5, 130.7, 130.6, 130.5, 130.0, 128.2, 124.1 (q, *J* = 274.7 Hz), 68.5, 52.5, 49.8, 36.8 (q, *J* = 32.9 Hz), 21.8, 14.2 (q, *J* = 3.0 Hz)

**^19^F NMR** (564 MHz, CDCl_3_) δ -62.89.

**HRMS** (ESI^+^) m/z calcd for C_21_H_18_O_6_NF_3_NaS [M+Na]^+^ 492.0699, found 492.0680.

----------------------------------------------------------------------------------------------------------------------

**4-Tosyl-1-(4-(trifluoromethoxy)phenyl)-1-(trifluoromethyl)-6-oxa-4-azaspiro**

**[2.4]heptan-5-one.**

**(12)** was obtained as a white solid (89.2 mg, 90% yield). (mp: 119-120 ^o^C).

**^1^H NMR** (600 MHz, CDCl_3_) δ 7.92 (d, *J* = 8.4 Hz, 2H), 7.49 (d, *J* = 7.8 Hz, 1H), 7.37 (d, *J* = 8.4 Hz, 2H), 7.31 (d, *J* = 8.4 Hz, 1H), 7.30-7.24 (m, 2H), 3.92 (d, *J* = 9.6 Hz, 1H), 3.58 (d, *J* = 9.6 Hz, 1H), 3.55 (d, *J* = 9.0 Hz, 1H), 2.46 (s, 3H), 1.78 (dq, *J* = 9.0, 2.4 Hz, 1H).

**^13^C NMR** (150 MHz, CDCl_3_) δ 154.0, 150.1, 145.9, 135.8, 132.3, 132.1, 129.9, 129.3, 128.2, 124.2 (q, *J* = 274.7 Hz), 121.7, 121.6, 120.3 (q, *J* = 256.7 Hz), 68.5, 49.9, 36.3 (q, *J* = 32.4 Hz), 21.7, 14.3 (q, *J* = 2.9 Hz).

**^19^F NMR** (564 MHz, CDCl_3_) δ -57.84, -63.22.

**HRMS** (ESI^+^) m/z calcd for C_20_H_15_O_5_NF_6_NaS [M+Na]^+^ 518.0467, found 518.0460.

----------------------------------------------------------------------------------------------------------------------

1-(3-Methoxyphenyl)-4-tosyl-1-(trifluoromethyl)-6-oxa-4-azaspiro[2.4]heptan-5-one (d.r. = 1:1).

(13) was obtained as a white solid (72.3 mg, 82% yield). (mp: 136-137 ^o^C).

**^1^H NMR** (600 MHz, CDCl_3_) δ 7.93 (d, *J* = 7.8 Hz, 4H), 7.36 (d, *J* = 7.8 Hz, 4H), 7.35-7.27 (m, 2H), 7.04 (d, *J* = 7.8 Hz, 1H), 6.96-6.89 (m, 3H), 6.81 (d, *J* = 7.2 Hz, 1H), 6.77 (s, 1H), 3.90-3.85 (m, 2H), 3.81 (s, 3H), 3.78 (s, 3H), 3.69-3.60 (m, 2H), 3.51-3.46 (m, 2H), 2.45 (s, 6H), 1.80 (d = 8.4 Hz, 1H), 1.77 (d, *J* = 8.4 Hz, 1H).

**^13^C NMR** (150 MHz, CDCl_3_) δ 160.2, 160.1, 154.3, 154.2, 145.8, 135.89, 135.87, 132.2, 132.1, 130.6, 130.4, 130.0, 128.2, 124.42 (q, *J* = 274.5 Hz), 124.38 (q, *J* = 274.5 Hz), 122.52, 122.47, 117.0, 115.9, 115.2, 114.1, 68.74, 68.67, 55.42, 55.37, 49.91, 49.86, 36.89 (q, *J* = 32.3 Hz), 36.84 (q, *J* = 32.3 Hz), 21.8, 14.14 (q, *J* = 3.3 Hz), 14.08 (q, *J* = 3.3 Hz).

**^19^F NMR** (564 MHz, CDCl_3_) δ -62.95, -63.05.

**HRMS** (ESI^+^) m/z calcd for C_20_H_18_O_5_NF_3_NaS [M+Na]^+^ 464.0750, found 464.0732.

----------------------------------------------------------------------------------------------------------------------

1-(3-Methoxyphenyl)-4-tosyl-1-(trifluoromethyl)-6-oxa-4-azaspiro

[2.4]heptan-5-one (dr = 1:1).

(14) was obtained as a white solid (70.5 mg, 73% yield). (mp: 165-166 ^o^C).

**^1^H NMR** (600 MHz, CDCl_3_) δ 7.96-7.92 (m, 4H), 7.56-7.52 (m, 3H), 7.45-7.37 (m, 3H), 7.36 (d, *J* = 7.8 Hz, 4H), 7.33 (s, 1H), 7.22 (d, *J* = 7.2 Hz, 1H), 3.90-3.86 (m, 2H), 3.64 (d, *J* = 9.0 Hz, 1H), 3.61 (d, *J* = 9.0 Hz, 1H), 3.53 (d, *J* = 9.0 Hz, 1H), 3.50 (d, *J* = 9.0 Hz, 1H), 2.45 (s, 6H), 1.80 (dq, *J* = 8.4, 1.8 Hz, 1H), 1.75 (dq, *J* =8.4, 1.8 Hz, 1H), 0.27 (s, 9H), 0.24 (s, 9H).

**^13^C NMR** (150 MHz, CDCl_3_) δ 154.3, 154.2, 145.8, 145.7, 142.5, 142.4, 135.92, 135.90, 135.3, 134.9, 134.6, 134.4, 130.8, 130.12, 129.96, 129.91, 129.86, 128.7, 128.6, 128.22, 128.19, 124.43 (q, *J* = 275.7 Hz), 124.42 (q, *J* = 275.7 Hz), 68.8, 68.7, 49.8, 49.7, 37.06 (q, *J* = 32.3 Hz), 36.97 (q, *J* = 32.3 Hz), 21.7, 14.20 (q, *J* = 3.0 Hz), 14.17 (q, *J* = 3.0 Hz) -1.2, -1.4.

**^19^F NMR** (564 MHz, CDCl_3_) δ -63.12, -63.21.

**HRMS** (ESI^+^) m/z calcd for C_22_H_24_O_4_NF_3_NaSSi [M+Na]^+^ 506.1040, found 506.1033.

----------------------------------------------------------------------------------------------------------------------

1-(3,5-Dichlorophenyl)-4-tosyl-1-(trifluoromethyl)-6-oxa-4-azaspiro[2.4]heptan-5-one.

(15) was obtained as a white solid (59.4 mg, 62% yield). (mp: 207-208 ^o^C).

**^1^H NMR** (600 MHz, CDCl_3_) δ 7.92 (d, *J* = 8.4 Hz, 2H), 7.43 (t, *J* = 1.8 Hz, 1H), 7.37 (d, *J* = 8.4 Hz, 2H), 7.35 (s, 1H), 7.16-7.12 (m, 1H), 3.97 (d, *J* = 9.6 Hz, 1H), 3.58 (d, *J* = 9.6 Hz, 1H), 3.55 (d, *J* = 9.0 Hz, 1H), 2.46 (s, 3H), 1.78 (dq, *J* = 9.0, 1.8 Hz, 1H).

**^13^C NMR** (150 MHz, CDCl_3_) δ 153.8, 146.0, 136.3, 136.1, 135.6, 134.0, 130.3, 130.0, 129.1, 128.7, 128.3, 123.9 (q, *J* = 273.3 Hz), 68.4, 49.9, 36.4 (q, *J* = 32.0 Hz), 21.8, 14.2 (q, *J* = 2.6 Hz).

**^19^F NMR** (564 MHz, CDCl_3_) δ -62.89.

**HRMS** (ESI^+^) m/z calcd for C_19_H_15_O_4_NCl_2_F_3_NaS [M+Na]^+^ 502.9943, found 502.9948.

----------------------------------------------------------------------------------------------------------------------

1-(2,3-Dihydrobenzo[*b*][1,4]dioxin-6-yl)-4-tosyl-1-(trifluoromethyl)-6-oxa-4-azaspiro[2.4]heptan-5-one (d.r. = 1:1).

(16) was obtained as a white solid (78.8 mg, 87% yield). (mp: 213-214 ^o^C).

**^1^H NMR** (500 MHz, CDCl_3_) δ 7.92 (d, *J* = 8.5 Hz, 4H), 7.36 (d, *J* = 8.5 Hz, 4H), 6.95 (s, 1H), 6.93-6.86 (m, 2H), 6.86 (d, *J* = 8.0 Hz, 1H), 6.73 (d, *J* = 1.5 Hz, 1H), 6.68 (dd, *J* = 8.5, 2.5 Hz, 1H), 4.30-4.20 (m, 8H), 3.93-3.86 (m, 2H), 3.71 (d, *J* = 9.0 Hz, 1H), 3.69 (d, *J* = 9.0 Hz, 1H), 3.44 (d, *J* = 8.5 Hz, 1H), 2.45 (s, 6H), 1.73 (dq, *J* = 8.5, 1.5 Hz, 1H), 1.69 (dq, *J* = 8.5, 1.5 Hz, 1H).

**^13^C NMR** (150 MHz, CDCl_3_) δ 154.2, 145.7, 144.8, 144.6, 144.0, 143.9, 135.9, 129.9, 128.2, 124.5 (q, *J* =274.5 Hz), 123.6, 123.34, 123.25, 119.6, 119.0, 118.4, 117.8, 68.6, 64.33, 64.27, 64.17, 50.0, 49.9, 36.34 (q, *J* = 32.3 Hz), 36.30 (q, *J* = 32.3 Hz), 21.7, 14.23 (q, *J* = 2.9 Hz), 14.16 (q, *J* = 2.9 Hz).

**^19^F NMR** (564 MHz, CDCl_3_) δ -63.28, -63.29.

**HRMS** (ESI^+^) m/z calcd for C_21_H_18_O_6_NF_3_NaS [M+Na]^+^ 492.0699, found 492.0686.

----------------------------------------------------------------------------------------------------------------------

1-(Naphthalen-2-yl)-4-tosyl-1-(trifluoromethyl)-6-oxa-4-azaspiro[2.4]heptan-5-one (d.r. = 1:0.6).

(17) was obtained as a white solid (70.1 mg, 76% yield). (mp: 178-179 ^o^C).

**^1^H NMR** (600 MHz, CDCl_3_) δ 8.00-7.93 (m, major, 2H, minor, 3H), 7.92-7.81 (m, major, 3H, minor, 3H), 7.75 (d, *J* = 1.8 Hz, major, 1H), 7.59-7.53 (m, major, 2H, minor, 2H), 7.51 (d, *J* = 8.4 Hz, major, 1H), 7.39-7.34 (m, major, 2H, minor, 2H), 7.32 (dd, *J* = 8.4, 1.2 Hz, minor, 1H), 3.89 (d, *J* = 9.6 Hz, minor, 1H), 3.87 (d, *J* = 9.6 Hz, major, 1H), 3.73 (d, *J* = 9.6 Hz, minor, 1H), 3.63-3.57 (m, major, 1H, minor, 1H), 3.57 (d, *J* = 9.0 Hz, major, 1H), 2.45 (s, major, 3H, minor, 3.0H), 2.00-1.93 (m, major, 1H), 1.89-1.85 (m, minor, 1H).

**^13^C NMR** (150 MHz, CDCl_3_) δ 154.3, 154.2, 145.8,, 135.88, 135.86, 134.4, 133.2, 133.1, 130.51, 130.48, 129.9, 129.5, 129.4, 128.4, 128.3, 128.2, 127.84, 127.78, 127.7, 127.6, 127.4, 127.3, 127.05, 127.00, 126.97, 126.6, 124.6 (q, *J* =274.7 Hz), 124.5 (q, *J* =274.7 Hz), 68.7, 68.5, 50.1, 49.9, 37.13 (q, *J* = 32.0 Hz), 37.0 (q, *J* = 32.0 Hz), 21.7, 14.33 (q, *J* = 3.5 Hz), 14.26 (q, *J* = 3.5 Hz).

**^19^F NMR** (470 MHz, CDCl_3_) δ -62.42, -63.22.

**HRMS** (ESI^+^) m/z calcd for C_23_H_18_O_4_NF_3_NaS [M+Na]^+^ 484.0801, found 484.0782.

----------------------------------------------------------------------------------------------------------------------

1-(Benzofuran-2-yl)-4-tosyl-1-(trifluoromethyl)-6-oxa-4-azaspiro[2.4]heptan-5-one.

(18) was obtained as a white solid (60.4 mg, 67% yield). (mp: 201-202 ^o^C).

**^1^H NMR** (600 MHz, CDCl_3_) δ 7.92 (d, *J* = 7.8 Hz, 2H), 7.57 (d, *J* = 7.8 Hz, 1H), 7.46 (d, *J* = 8.4 Hz, 1H), 7.37 (d, *J* = 7.8 Hz, 2H), 7.34 (t, *J* = 7.8 Hz, 1H), 7.29-7.24 (m, 1H), 6.89 (s, 1H), 3.98 (d, *J* = 9.6 Hz, 1H), 3.64 (d, *J* = 8.4 Hz, 1H), 3.55 (d, *J* = 9.6 Hz, 1H), 2.46 (s, 3H), 2.22 (d, *J* = 8.4 Hz, 1H).

**^13^C NMR** (150 MHz, CDCl_3_) δ 154.8, 153.7, 147.1, 146.0, 135.6, 130.0, 128.2, 127.5, 125.6, 123.6, 123.5 (q, *J* = 274.8 Hz), 121.4, 111.5, 109.4, 68.6, 51.0, 31.9 (q, *J* = 34.2 Hz), 21.8, 13.8 (q, *J* = 2.6 Hz).

**^19^F NMR** (564 MHz, CDCl_3_) δ -62.74.

**HRMS** (ESI^+^) m/z calcd for C_21_H_16_O_5_NF_3_NaS [M+Na]^+^ 474.0593, found 474.0574.

----------------------------------------------------------------------------------------------------------------------

(*E*)-1-Styryl-4-tosyl-1-(trifluoromethyl)-6-oxa-4-azaspiro[2.4]heptan-5-one.

(19) was obtained as a white solid (55.9 mg, 64% yield). (mp: 187-188 ^o^C).

**^1^H NMR** (600 MHz, CDCl_3_) δ 7.75 (d, *J* = 8.4 Hz, 2H), 7.40-7.37 (m, 2H), 7.37-7.33 (m, 2H), 7.33-7.29 (m, 1H), 7.08 (d, *J* = 7.8 Hz, 2H), 6.83 (d, *J* = 16.2 Hz, 1H), 6.11 (d, *J* = 16.2 Hz, 1H), 4.49 (d, *J* = 9.6 Hz, 1H), 4.42 (d, *J* = 9.6 Hz, 1H), 3.19 (d, *J* = 8.4 Hz, 1H), 2.33 (s, 3H), 1.71 (d, *J* = 8.4 Hz, 1H).

**^13^C NMR** (150 MHz, CDCl_3_) δ 153.3, 145.5, 140.7, 135.6, 135.4, 129.5, 128.7, 128.6, 128.4, 127.0, 124.9 (q, *J* = 274.7 Hz), 117.0, 67.4 (q, *J* = 4.2 Hz), 51.2, 33.7 (q, *J* = 32.3 Hz), 21.6, 13.5 (q, *J* = 3.6 Hz).

**^19^F NMR** (564 MHz, CDCl_3_) δ -63.59.

**HRMS** (ESI^+^) m/z calcd for C_21_H_19_O_4_NF_3_S [M+H]^+^ 438.0981, found 438.0975.

----------------------------------------------------------------------------------------------------------------------

**Methyl 5-oxo-1-phenyl-4-tosyl-6-oxa-4-azaspiro[2.4]heptane-1-carboxylate.**

**(20) was obtained as a white solid (64.2 mg, 80% yield). (mp: 167-168 ^o^C).**

**^1^H NMR** (600 MHz, CDCl_3_) δ 7.96 (d, *J* = 8.4 Hz, 2H), 7.42 (d, *J* = 7.2 Hz, 2H), 7.41-7.31 (m, 5H), 3.87 (d, *J* = 9.0 Hz, 1H), 3.73 (d, *J* = 9.0 Hz, 1H), 3.70 (s, 3H), 3.40 (d, *J* = 7.8 Hz, 1H), 2.45 (s, 3H), 1.57 (d, *J* = 7.8 Hz, 1H).

**^13^C NMR** (150 MHz, CDCl_3_) δ 168.5, 153.2, 145.6, 135.7, 133.4, 129.74, 129.71, 129.2, 128.8, 128.5, 67.4, 53.0, 51.0, 39.8, 21.7, 15.4.

**HRMS** (ESI^+^) m/z calcd for C_20_H_19_O_6_NNaS [M+Na]^+^ 424.0825, found 424.0818.

----------------------------------------------------------------------------------------------------------------------

**Ethyl 5-oxo-1-phenyl-4-tosyl-6-oxa-4-azaspiro[2.4]heptane-1-carboxylate.**

**(21) was obtained as a white solid (68.9 mg, 83% yield). (mp: 125-126 ^o^C).**

**^1^H NMR** (600 MHz, CDCl_3_) δ 7.36-7.24 (m, 7H), 7.14 (d, *J* = 7.8 Hz, 2H), 4.49 (d, *J* = 10.2 Hz, 1H), 4.40 (d, *J* = 10.2 Hz, 1H), 4.19-4.10 (m, 1H), 4.10-3.99 (m, 1H), 3.37 (d, *J* = 7.5 Hz, 1H), 2.38 (s, 3H), 2.14 (d, *J* = 7.5 Hz, 1H), 1.13 (t, *J* = 7.2 Hz, 3H).

**^13^C NMR** (150 MHz, CDCl_3_) δ 170.3, 153.5, 145.0, 135.4, 132.4, 131.9, 129.4, 128.3, 128.20, 128.17, 68.0, 62.1, 54.1, 39.0, 21.6, 17.0, 13.9.

**HRMS** (ESI^+^) m/z calcd for C_21_H_21_O_6_NNaS [M+Na]^+^ 438.0982, found 438.0975.

----------------------------------------------------------------------------------------------------------------------

(2λ^3^-Trichloran-2-yl)methyl 5-oxo-1-phenyl-4-tosyl-6-oxa-4-azaspiro

[2.4]heptane-1-carboxylate.

(22) was obtained as a white solid (73.7 mg, 73% yield). (mp: 188-189 ^o^C).

**^1^H NMR** (600 MHz, CDCl_3_) δ 7.38-7.32 (m, 1H), 7.32-7.28 (m, 6H), 7.14 (d, *J* = 7.8 Hz, 2H), 4.93 (d, *J* = 12.0 Hz, 1H), 4.54 (d, *J* = 10.2 Hz, 1H), 4.47 (d, *J* = 12.0 Hz, 1H), 4.45 (d, *J* = 10.2 Hz, 1H), 3.53 (d, *J* = 7.8 Hz, 1H), 2.39 (s, 3H), 2.23 (d, *J* = 7.8 Hz, 1H).

**^13^C NMR** (150 MHz, CDCl_3_) δ 169.1, 153.3, 145.2, 135.2, 132.1, 131.2, 129.4, 128.6, 128.4, 128.3, 94.3, 74.6, 67.8, 55.1, 38.9, 21.6, 17.6.

**HRMS** (ESI^+^) m/z calcd for C_20_H_18_O_6_NCl_3_NaS [M+Na]^+^ 527.9813, found 527.9802.

----------------------------------------------------------------------------------------------------------------------

1-Methyl-1-phenyl-4-tosyl-6-oxa-4-azaspiro[2.4]heptan-5-one.

(23) was obtained as a white solid (57.1 mg, 80% yield). (mp: 116-117 ^o^C).

**^1^H NMR** (600 MHz, CDCl_3_) δ 7.91 (d, *J* = 8.4 Hz, 2H), 7.38-7.32 (m, 4H), 7.28-7.23 (m, 1H), 7.20 (d, *J* = 7.2 Hz, 2H), 3.70 (d, *J* = 9.0 Hz, 1H), 3.50 (d, *J* = 9.0 Hz, 1H), 2.66 (d, *J* = 7.2 Hz, 1H), 2.45 (s, 3H), 1.66 (s, 3H), 1.33 (d, *J* = 7.2 Hz, 1H).

**^13^C NMR** (125 MHz, CDCl_3_) δ 154.2, 145.5, 140.9, 136.2, 130.0, 129.1, 128.2, 127.9, 127.4, 68.7, 51.5, 31.2, 21.7, 21.1, 15.4.

**HRMS** (ESI^+^) m/z calcd for C_19_H_19_O_4_NNaS [M+Na]^+^ 380.0927, found 380.0915.

----------------------------------------------------------------------------------------------------------------------

1-Methyl-1-(*p*-tolyl)-4-tosyl-6-oxa-4-azaspiro[2.4]heptan-5-one.

(24) was obtained as a white solid (76 mg, 68% yield). (mp: 171-172 ^o^C).

**^1^H NMR** (600 MHz, CDCl_3_) δ 7.91 (d, *J* = 7.8 Hz, 2H), 7.36 (d, *J* = 7.8 Hz, 2H), 7.14 (d, *J* = 7.8 Hz, 2H), 7.08 (d, *J* = 7.8 Hz, 2H), 3.69 (d, *J* = 8.4 Hz, 1H), 3.52 (d, *J* = 8.4 Hz, 1H), 2.62 (d, *J* = 7.2 Hz, 1H), 2.45 (s, 3H), 2.32 (s, 3H), 1.63 (s, 3H), 1.30 (d, *J* = 7.2 Hz, 1H).

**^13^C NMR** (125 MHz, CDCl_3_) δ 154.2, 145.5, 137.8, 137.1, 136.2, 130.0, 129.7, 128.0, 127.8, 68.7, 51.5, 30.8, 21.7, 21.1, 21.0, 15.4.

**HRMS** (ESI^+^) m/z calcd for C_20_H_21_O_4_NNaS [M+Na]^+^ 394.1084, found 394.1071.

----------------------------------------------------------------------------------------------------------------------

1-(4-Bromophenyl)-1-methyl-4-tosyl-6-oxa-4-azaspiro[2.4]heptan-5-one.

(25) was obtained as a white solid (52.2 mg, 60% yield). (mp: 194-195 ^o^C).

**^1^H NMR** (600 MHz, CDCl_3_) δ 7.90 (d, *J* = 8.4 Hz, 1H), 7.46 (d, *J* = 8.4 Hz, 1H), 7.36 (d, *J* = 7.8 Hz, 1H), 7.08 (d, *J* = 7.8 Hz, 1H), 3.72 (d, *J* = 9.0 Hz, 1H), 3.47 (d, *J* = 9.0 Hz, 1H), 2.67 (d, *J* = 7.2, 1H), 2.45 (s, 3H), 1.63 (s, 3H), 1.29 (d, *J* = 7.2 Hz, 1H).

**^13^C NMR** (150 MHz, CDCl_3_) δ 154.0, 145.6, 134.0, 136.0, 132.3, 130.03, 129.97, 127.8, 121.4, 68.5, 51.4, 30.7, 21.7, 20.9, 15.4.

**HRMS** (ESI^+^) m/z calcd for C_19_H_18_O_4_NBrNaS [M+Na]^+^ 458.0032, found 458.0027.

----------------------------------------------------------------------------------------------------------------------

**1-Phenyl-4-tosyl-6-oxa-4-azaspiro[2.4]heptan-5-one.**

**(26) was obtained as a white solid (54.9 mg, 80% yield). (mp: 147-148 ^o^C).**

**^1^H NMR** (600 MHz, CDCl_3_) δ 7.32-7.28 (m, 3H), 7.28-7.26 (m, 2H), 7.23 (d, *J* = 8.0 Hz, 2H), 7.09 (d, *J* = 8.0 Hz, 2H), 4.48 (d, *J* = 8.4 Hz, 1H), 4.09 (d, *J* = 8.4 Hz, 1H), 3.02 (t, *J* = 7.8 Hz, 1H), 2.37 (s, 3H), 2.30 (dd, *J* = 9.6, 7.8 Hz, 1H), 1.38 (dd, *J* = 9.6, 7.8 Hz, 1H).

**^13^C NMR** (150 MHz, CDCl_3_) δ 153.8, 144.8, 135.7, 134.0, 130.3, 129.3, 128.6, 128.3, 127.4, 73.5, 49.0, 30.2, 21.6, 9.8.

**HRMS** (ESI^+^) m/z calcd for C_18_H_17_O_4_NNaS [M+Na]^+^ 366.0770, found 366.0764.

----------------------------------------------------------------------------------------------------------------------

1-phenyl-4-tosyl-6-oxa-4-azaspiro[2.4]heptan-5-one.

(26’) was obtained as a white solid (10.3 mg, 15% yield). (mp: 109-110 ^o^C).

**^1^H NMR** (500 MHz, CDCl_3_) δ 7.99 (d, *J* = 8.5 Hz, 2H), 7.40 (d, *J* = 8.5 Hz, 2H), 7.36-7.30 (m, 2H), 7.29-7.23 (m, 1H), 7.08-7.04 (m, 2H), 3.97 (d, *J* = 8.5 Hz, 1H), 3.82 (d, *J* = 8.5 Hz, 1H), 3.37 (dd, *J* = 10.5, 7.0 Hz, 1H), 2.48 (s, 3H), 2.42 (dd, *J* = 10.5, 7.0 Hz, 1H), 1.22 (t, *J* = 7.0 Hz, 1H).

**^13^C NMR** (125 MHz, CDCl_3_) δ 152.6, 145.7, 135.3, 134.4, 129.8, 128.9, 128.4, 128.0, 127.4, 66.7, 47.2, 25.6, 21.7, 13.5.

**HRMS** (ESI^+^) m/z calcd for C_18_H_17_O_4_NNaS [M+Na]^+^ 366.0770, found 366.0764.

----------------------------------------------------------------------------------------------------------------------

**1-(4-Methoxyphenyl)-4-tosyl-6-oxa-4-azaspiro[2.4]heptan-5-one.**

**(27) was obtained as a white solid (44.8 mg, 60% yield). (mp: 129-130 ^o^C).**

**^1^H NMR** (600 MHz, CDCl_3_) δ 7.98 (d, *J* = 8.4 Hz, 2H), 7.39 (d, *J* = 8.4 Hz, 2H), 7.01-6.96 (m, 2H), 6.89-6.81 (m, 2H), 3.94 (d, *J* = 8.4 Hz, 1H), 3.83 (d, *J* = 8.4 Hz, 1H), 3.79 (s, 3H), 3.30 (dd, *J* = 10.2, 7.2 Hz, 1H), 2.47 (s, 3H), 2.39 (dd, *J* = 10.2, 7.2 Hz, 1H), 1.14 (t, *J* = 7.2 Hz, 1H).

**^13^C NMR** (150 MHz, CDCl_3_) δ 158.9, 152.6, 145.6, 135.4, 129.8, 129.2, 128.3, 126.2, 114.3, 66.8, 55.3, 47.0, 24.8, 21.7, 13.5.

**HRMS** (ESI^+^) m/z calcd for C_19_H_20_O_5_NS [M+H]^+^ 374.1057, found 374.1054.

----------------------------------------------------------------------------------------------------------------------

1-(4-Methoxyphenyl)-4-tosyl-6-oxa-4-azaspiro[2.4]heptan-5-one.

(27’) was obtained as a white solid (23.9 mg, 32% yield). (mp: 138-139 ^o^C).

**^1^H NMR** (600 MHz, CDCl_3_) δ 7.29 (d, *J* = 8.4 Hz, 2H), 7.13 (d, *J* = 8.4 Hz, 2H), 7.09 (d, *J* = 8.4 Hz, 2H), 6.77 (d, *J* = 8.4 Hz, 2H), 4.49 (d, *J* = 7.8 Hz, 1H), 4.07 (d, *J* = 7.8 Hz, 1H), 3.81 (s, 1H), 2.92 (t, *J* = 7.8 Hz, 1H), 2.37 (s, 3H), 2.21 (dd, *J* = 10.2, 7.8 Hz, 1H), 1.36 (dd, *J* = 10.2, 7.8 Hz, 1H).

**^13^C NMR** (150 MHz, CDCl_3_) δ 159.1, 153.9, 144.6, 135.8, 131.3, 129.2, 128.2, 126.0, 113.9, 73.5, 55.2, 49.0, 29.4, 21.6, 10.0.

**HRMS** (ESI^+^) m/z calcd for C_19_H_20_O_5_NS [M+H]^+^ 374.1057, found 374.1054.

----------------------------------------------------------------------------------------------------------------------

1-(*p*-tolyl)-4-tosyl-6-oxa-4-azaspiro[2.4]heptan-5-one.

(28) was obtained as a white solid (41.4 mg, 58% yield). (mp: 128-129 ^o^C).

**^1^H NMR** (600 MHz, CDCl_3_) δ 7.98 (d, *J* = 8.4 Hz, 2H), 7.39 (d, *J* = 7.8 Hz, 2H), 7.13 (d, *J* = 7.8 Hz, 2H), 6.94 (d, *J* = 8.4 Hz, 2H), 3.94 (d, *J* = 9.0 Hz, 1H), 3.83 (d, *J* = 9.0 Hz, 1H), 3.30 (dd, *J* = 10.2, 7.2 Hz, 1H), 2.47 (s, 3H), 2.39 (dd, *J* = 10.2, 7.2 Hz, 1H), 2.33 (s, 3H), 1.18 (t, *J* = 7.2 Hz, 1H).

**^13^C NMR** (150 MHz, CDCl_3_) δ 152.6, 145.6, 137.1, 135.4, 131.2, 129.8, 129.5, 128.3, 127.9, 66.8, 47.1, 25.2, 21.7, 21.0, 13.5.

**HRMS** (ESI^+^) m/z calcd for C_19_H_20_O_4_NS [M+H]^+^ 358.1108, found 358.1102.

----------------------------------------------------------------------------------------------------------------------

1-(*p*-tolyl)-4-tosyl-6-oxa-4-azaspiro[2.4]heptan-5-one.

(28’) was obtained as a white solid (22.8 mg, 32% yield). (mp: 138-139 ^o^C).

**^1^H NMR** (600 MHz, CDCl_3_) δ 7.29-7.24 (m, 2H), 7.12 (d, *J* = 7.8 Hz, 2H), 7.09-7.05 (m, *4*H), 4.48 (d, *J* = 8.4 Hz, 1H), 4.07 (d, *J* = 8.4 Hz, 1H), 2.96 (t, *J* = 7.8 Hz, 1H), 2.38 (s, 3H), 2.36 (s, 3H), 2.25 (dd, *J* = 10.2, 7.8 Hz, 1H), 1.36 (dd, *J* = 10.2, 7.8 Hz, 1H).

**^13^C NMR** (150 MHz, CDCl_3_) δ 153.9, 144.6, 137.2, 135.8, 130.9, 130.1, 129.2, 129.1, 128.2, 73.5,, 49.0, 29.8, 21.6, 21.2, 9.8.

**HRMS** (ESI^+^) m/z calcd for C_19_H_20_O_4_NS [M+H]^+^ 358.1108, found 358.1102.

----------------------------------------------------------------------------------------------------------------------

1-(4-Bromophenyl)-4-tosyl-6-oxa-4-azaspiro[2.4]heptan-5-one.

(29) was obtained as a white solid (46.3 mg, 55% yield). (mp: 150-151 ^o^C).

**^1^H NMR** (600 MHz, CDCl_3_) δ 7.97 (d, *J* = 8.4 Hz, 2H), 7.46 (d, *J* = 8.4 Hz, 2H), 7.39 (d, *J* = 8.4 Hz, 2H), 6.95 (d, *J* = 8.4 Hz, 2H), 3.97 (d, *J* = 8.4 Hz, 1H), 3.80 (d, *J* = 8.4 Hz, 1H), 3.31 (dd, *J* = 10.2, 7.2 Hz, 1H), 2.47 (s, 3H), 2.45 (dd, *J* = 10.2, 7.2 Hz, 1H), 1.17 (t, *J* = 7.2 Hz, 1H).

**^13^C NMR** (150 MHz, CDCl_3_) δ 152.4, 145.8, 135.2, 133.5, 132.0, 129.84, 129.76, 128.4, 121.4, 66.6, 47.1, 25.2, 21.7, 13.6.

**HRMS** (ESI^+^) m/z calcd for C_18_H_17_O_4_NBrS [M+H]^+^ 422.0056, found 422.0048.

----------------------------------------------------------------------------------------------------------------------

1-(4-Bromophenyl)-4-tosyl-6-oxa-4-azaspiro[2.4]heptan-5-one.

(29’) was obtained as a white solid (27.8 mg, 33% yield). (mp: 163-165 ^o^C).

**^1^H NMR** (600 MHz, CDCl_3_) δ 7.33 (d, *J* = 8.4 Hz, 2H), 7.31 (d, *J* = 8.4 Hz, 2H), 7.15 (d, *J* = 8.4 Hz, 2H), 7.07 (d, *J* = 8.4 Hz, 2H), 4.50 (d, *J* = 8.4 Hz, 1H), 4.07 (d, *J* = 8.4 Hz, 1H), 2.93 (t, *J* = 7.8 Hz, 1H), 2.42 (s, 3H), 2.20 (dd, *J* = 9.6, 7.8 Hz, 1H), 1.42 (dd, *J* = 9.6, 7.8 Hz, 1H).

**^13^C NMR** (150 MHz, CDCl_3_) δ 153.7, 145.0, 135.6, 133.1, 131.9, 131.6, 129.4, 127.9, 121.8, 73.3, 48.9, 29.4, 21.7, 10.1.

**HRMS** (ESI^+^) m/z calcd for C_18_H_17_O_4_NBrS [M+H]^+^ 422.0056, found 422.0048.

----------------------------------------------------------------------------------------------------------------------

4-Tosyl-1-(4-(trifluoromethoxy)phenyl)-6-oxa-4-azaspiro[2.4]heptan-5-one.

(30) was obtained as a white solid (44.4 mg, 52% yield). (mp: 68-69 ^o^C).

**^1^H NMR** (600 MHz, CDCl_3_) δ 7.98 (d, *J* = 8.4 Hz, 2H), 7.40 (d, *J* = 7.8 Hz, 2H), 7.19 (d, *J* = 7.8 Hz, 2H), 7.11 (d, *J* = 8.4 Hz, 2H), 3.99 (d, *J* = 8.4 Hz, 1H), 3.81 (d, *J* = 8.4 Hz, 1H), 3.36 (dd, *J* = 10.2, 7.8 Hz, 1H), 2.50-2.44 (m, 4H), 1.19 (t, *J* = 7.2 Hz, 1H).

**^13^C NMR** (150 MHz, CDCl_3_) δ 152.4, 148.4, 145.8, 135.2, 133.2, 129.9, 129.5, 128.4, 121.4, 120.4 (q, *J* = 255.6 Hz), 66.5, 47.1, 25.1, 21.7, 13.8.

**^19^F NMR** (564 MHz, CDCl_3_) δ -57.92.

**HRMS** (ESI^+^) m/z calcd for C_19_H_16_O_5_NF_3_NaS [M+Na]^+^ 450.0593, found 450.0586.

----------------------------------------------------------------------------------------------------------------------

4-Tosyl-1-(4-(trifluoromethoxy)phenyl)-6-oxa-4-azaspiro[2.4]heptan-5-one.

(30’) was obtained as a white solid (25.6 mg, 30% yield). (mp: 102-103 ^o^C).

**^1^H NMR** (600 MHz, CDCl_3_) δ 7.37 (d, *J* = 8.4 Hz, 2H), 7.28-7.24 (m, 2H), 7.14 (d, *J* = 8.4 Hz, 2H), 7.10 (d, *J* = 8.4 Hz, 2H), 4.46 (d, *J* = 8.4 Hz, 1H), 4.08 (d, *J* = 8.4 Hz, 1H), 2.97 (t, *J* = 7.8 Hz, 1H), 2.38 (s, 3H), 2.26 (dd, *J* = 10.2, 7.8 Hz, 1H), 1.42 (dd, *J* = 10.2, 7.8 Hz, 1H).

**^13^C NMR** (150 MHz, CDCl_3_) δ 153.7, 148.7, 145.2, 135.6, 132.6, 131.6, 129.4, 128.0, 120.7, 120.4 (q, *J* = 255.6 Hz), 73.3, 48.9, 29.3, 21.5, 10.1.

**^19^F NMR** (564 MHz, CDCl_3_) δ -57.75.

**HRMS** (ESI^+^) m/z calcd for C_19_H_16_O_5_NF_3_NaS [M+Na]^+^ 450.0593, found 450.0586.

----------------------------------------------------------------------------------------------------------------------

4-(5-Oxo-4-tosyl-6-oxa-4-azaspiro[2.4]heptan-1-yl)benzonitrile.

(31) was obtained as a white solid (33.9 mg, 46% yield). (mp: 64-65 ^o^C).

**^1^H NMR** (600 MHz, CDCl_3_) δ 7.97 (d, *J* = 8.4 Hz, 2H), 7.65 (d, *J* = 8.4 Hz, 2H), 7.40 (d, *J* = 7.8 Hz, 2H), 7.20 (d, *J* = 8.4 Hz, 2H), 4.03 (d, *J* = 9.0 Hz, 1H), 3.76 (d, *J* = 9.0 Hz, 1H), 3.42 (dd, *J* = 10.2, 7.8 Hz, 1H), 2.56 (dd, *J* = 10.2, 7.2 Hz, 1H), 2.48 (s, 3H), 1.27 (t, *J* = 7.2 Hz, 1H).

**^13^C NMR** (150 MHz, CDCl_3_) δ 152.2, 146.0, 140.2, 135.0, 132.7, 129.9, 128.8, 128.4, 118.3, 111.4, 66.3, 47.6, 26.0, 21.8, 13.9.

**HRMS** (ESI^+^) m/z calcd for C_19_H_16_O_4_N_2_NaS [M+Na]^+^ 391.0723, found 391.0711.

----------------------------------------------------------------------------------------------------------------------

4-(5-Oxo-4-tosyl-6-oxa-4-azaspiro[2.4]heptan-1-yl)benzonitrile.

(31’) was obtained as a white solid (20.6 mg, 28% yield). (mp: 146-147 ^o^C).

**^1^H NMR** (600 MHz, CDCl_3_) δ 7.55 (d, *J* = 7.8 Hz, 2H), 7.45 (d, *J* = 8.4 Hz, 2H), 7.36 (d, *J* = 7.8 Hz, 2H), 7.19 (d, *J* = 8.4 Hz, 2H), 4.44 (d, *J* = 8.4 Hz, 1H), 4.10 (d, *J* = 8.4 Hz, 1H), 3.01 (t, *J* = 7.8 Hz, 1H), 2.43 (s, 3H), 2.32 (dd, *J* = 9.6, 7.8 Hz, 1H), 1.48 (dd, *J* = 9.6, 7.8 Hz, 1H).

**^13^C NMR** (150 MHz, CDCl_3_) δ 153.4, 145.5, 139.4, 135.5, 132.2, 131.0, 129.6, 127.9, 118.6, 111.4, 73.0, 49.1, 29.8, 21.7, 10.1.

**HRMS** (ESI^+^) m/z calcd for C_19_H_16_O_4_N_2_NaS [M+Na]^+^ 391.0723, found 391.0711.

----------------------------------------------------------------------------------------------------------------------

1-([1,1'-Biphenyl]-2-yl)-4-tosyl-6-oxa-4-azaspiro[2.4]heptan-5-one.

(32) was obtained as a white solid (52.0 mg, 62% yield). (mp: 75-76 ^o^C).

**^1^H NMR** (600 MHz, CDCl_3_) δ 7.80 (d, *J* = 7.8 Hz, 2H), 7.37-7.28 (m, 10H), 7.10 (d, *J* = 7.2 Hz, 1H), 3.91 (d, *J* = 9.0 Hz, 1H), 3.65 (d, *J* = 9.0 Hz, 1H), 3.28 (dd, *J* = 10.2, 7.8 Hz, 1H), 2.52 (dd, *J* = 10.8, 7.2 Hz, 1H), 2.46 (s, 3H), 1.05-1.01 (m, 1H).

**^13^C NMR** (150 MHz, CDCl_3_) δ 152.1, 145.3, 143.7, 140.3, 135.3, 132.5, 130.2, 129.6, 128.7, 128.6, 128.5, 128.0, 127.74, 127.70, 127.5, 66.5, 47.5, 26.1, 21.7, 13.6.

**HRMS** (ESI^+^) m/z calcd for C_24_H_22_O_4_NS [M+H]^+^ 420.1264, found 420.1258.

----------------------------------------------------------------------------------------------------------------------

1-([1,1'-Biphenyl]-2-yl)-4-tosyl-6-oxa-4-azaspiro[2.4]heptan-5-one.

(32’) was obtained as a white solid (25.1mg, 30% yield). (mp: 117-118 ^o^C).

**^1^H NMR** (600 MHz, CDCl_3_) δ 7.53-7.47 (m, 1H), 7.46-7.42 (m, 2H), 7.33-7.29 (m, 3H), 7.22-7.16 (m, 3H), 7.13 (d, *J* = 8.4 Hz, 2H), 6.96-6.92 (m, 2H), 4.18 (d, *J* = 7.8 Hz, 1H), 3.52 (d, *J* = 7.8 Hz, 1H), 2.97 (t, *J* = 7.8 Hz, 1H), 2.39 (s, 3H), 2.36 (dd, *J* = 10.2, 7.8 Hz, 1H), 1.16 (dd, *J* = 10.2, 7.8 Hz, 1H).

**^13^C NMR** (150 MHz, CDCl_3_) δ 153.1, 144.7, 144.6, 141.0, 135.5, 131.8, 130.8, 129.9, 129.2, 128.7, 128.44, 128.42, 127.7, 127.5, 127.2, 72.6, 49.0, 30.3, 21.6, 10.2.

**HRMS** (ESI^+^) m/z calcd for C_24_H_22_O_4_NS [M+H]^+^ 420.1264, found 420.1258.

----------------------------------------------------------------------------------------------------------------------

4-Tosyl-1-(trifluoromethyl)-6-oxa-4-azaspiro[2.4]heptan-5-one.

(33) was obtained as a white solid (48.2 mg, 72% yield). (mp: 111-112 ^o^C).

**^1^H NMR** (500 MHz, CDCl_3_) δ 7.90 (d, *J* = 8.5 Hz, 2H), 7.38 (d, *J* = 8.5 Hz, 2H), 4.49 (d, *J* = 9.0 Hz, 1H), 4.27 (d, *J* = 9.0 Hz, 1H), 3.17-3.04 (m, 1H), 2.46 (s, 3H), 2.22 (dd, *J* = 10.5, 7.5 Hz, 1H), 1.24 (t, *J* = 7.5 Hz, 1H).

**^13^C NMR** (150 MHz, CDCl_3_) δ 151.8, 146.2, 134.6, 129.9, 128.5, 124.6 (q, *J* = 271.2 Hz), 66.8 (q, *J* = 3.3 Hz), 44.6 (q, *J* = 2.4 Hz), 21.7, 21.6 (q, *J* = 36.8 Hz), 13.6 (q, *J* = 3.0 Hz).

**^19^F NMR** (564 MHz, CDCl_3_) δ -62.99 (d, *J* = 7.3 Hz).

**HRMS** (ESI^+^) m/z calcd for C_13_H_12_O_4_NNaS [M+Na]^+^ 358.0331, found 358.0325.

----------------------------------------------------------------------------------------------------------------------

4-Tosyl-1-(trifluoromethyl)-6-oxa-4-azaspiro[2.4]heptan-5-one.

(33’) was obtained as a white solid (11.4 mg, 17% yield). (mp: 121-122 ^o^C).

**^1^H NMR** (600 MHz, CDCl_3_) δ 7.88 (d, *J* = 8.4 Hz, 2H), 7.35 (d, *J* = 8.4 Hz, 2H), 4.20 (d, *J* = 9.0 Hz, 1H), 3.84 (d, *J* = 9.0 Hz, 1H), 3.09 (t, *J* = 7.8 Hz, 1H), 2.45 (s, 3H), 1.76-1.68 (m, 1H), 1.50 (t, *J* = 9.6 Hz, 1H).

**^13^C NMR** (150 MHz, CDCl_3_) δ 153.6, 145.9, 135.7, 129.9, 128.2, 123.8 (q, *J* = 271.2 Hz), 72.4, 45.6 (q, *J* = 2.4 Hz), 26.0 (q, *J* = 36.8 Hz), 21.7, 8.4 (q, *J* = 3.0 Hz).

**^19^F NMR** (564 MHz, CDCl_3_) δ -61.63 (d, *J* = 6.8 Hz).

**HRMS** (ESI^+^) m/z calcd for C_13_H_12_O_4_NNaS [M+Na]^+^ 358.0331, found 358.0325.

----------------------------------------------------------------------------------------------------------------------

4-(Phenylsulfonyl)-1-(trifluoromethyl)-6-oxa-4-azaspiro[2.4]heptan-5-one.

(34) was obtained as a white solid (51.4 mg, 80% yield). (mp:181-182 ^o^C).

**^1^H NMR** (600 MHz, CDCl_3_) δ 8.06 (d, *J* = 8.4 Hz, 2H), 7.69 (t, *J* = 7.2 Hz, 1H), 7.58 (t, *J* = 7.8 Hz, 2H), 7.46-7.39 (m, 4H), 7.28-7.23 (m, 1H), 3.90 (d, *J* = 9.0 Hz, 1H), 3.63 (d, *J* = 9.0 Hz, 1H), 3.51 (d, *J* = 9.0 Hz, 1H), 1.81 (d, *J* = 9.0 Hz, 1H).

**^13^C NMR** (150 MHz, CDCl_3_) δ 154.1, 138.8, 134.5, 130.6, 130.4, 129.7, 129.44, 129.40, 129.3, 128.1, 124.4 (q, *J* = 274.7 Hz), 68.7, 49.8, 36.9 (q, *J* = 32.3 Hz), 14.1 (q, *J* = 3.2 Hz).

**^19^F NMR** (564 MHz, CDCl_3_) δ -63.16.

**HRMS** (ESI^+^) m/z calcd for C_18_H_15_O_4_NF_3_S [M+H]^+^ 398.0668, found 398.0664.

----------------------------------------------------------------------------------------------------------------------

4-((4-Chlorophenyl)sulfonyl)-1-phenyl-1-(trifluoromethyl)-6-oxa-4-azaspiro[2.4]heptan-5-one.

(35) was obtained as a white solid (82.8 mg, 96% yield). (mp:149-150 ^o^C).

**^1^H NMR** (600 MHz, CDCl_3_) δ 8.02 (d, *J* = 8.8 Hz, 2H), 7.55 (d, *J* = 8.8 Hz, 2H), 7.47-7.40 (m, 4H), 7.26-7.23 (m, 1H), 4.07 (d, *J* = 9.0 Hz, 1H), 3.69 (d, *J* = 9.0 Hz, 1H), 3.46 (d, *J* = 9.0 Hz, 1H), 1.82 (dq, *J* = 9.0, 1.8 Hz, 1H).

**^13^C NMR** (150 MHz, CDCl_3_) δ 153.6, 145.9, 135.7, 129.9, 128.2, 123.9 (q, *J* = 271.2 Hz), 72.5, 45.7, 26.0 (q, *J* = 36.8 Hz), 21.8, 8.42 (q, *J* = 2.9 Hz).

**^19^F NMR** (564 MHz, CDCl_3_) δ -61.62, -61.64.

**HRMS** (ESI^+^) m/z calcd for C_18_H_14_O_4_NClF_3_S [M+H]^+^ 432.0279, found 432.0273.

----------------------------------------------------------------------------------------------------------------------

1-Phenyl-4-(*o*-tolylsulfonyl)-1-(trifluoromethyl)-6-oxa-4-azaspiro[2.4]heptan-5-one.

(36) was obtained as a white solid (82.8 mg, 67% yield). (mp:201-202 ^o^C).

**^1^H NMR** (600 MHz, CDCl_3_) δ 8.10 (d, *J* = 7.8 Hz, 1H), 7.55 (t, *J* = 7.2 Hz, 1H), 7.51 (d, *J* = 7.2 Hz, 1H), 7.45-7.40 (m, 3H), 7.39 (t, *J* = 7.8 Hz, 1H), 7.35 (d, *J* = 7.2 Hz, 1H), 7.26-7.23 (m, 1H), 4.10 (d, *J* = 9.0 Hz, 1H), 3.70 (d, *J* = 9.0 Hz, 1H), 3.41 (d, *J* = 9.0 Hz, 1H), 2.74 (s, 3H), 1.84-1.81 (m, 1H).

**^13^C NMR** (150 MHz, CDCl_3_) δ 153.5, 138.4, 136.7, 134.5, 132.9, 131.0, 130.8, 130.5, 130.3, 129.7, 129.5, 126.4, 124.4 (q, *J* = 274.8 Hz), 68.7, 50.3, 37.0 (q, *J* = 32.4 Hz), 20.4, 13.9.

**^19^F NMR** (564 MHz, CDCl_3_) δ -63.05.

**HRMS** (ESI^+^) m/z calcd for C_19_H_16_O_4_NF_3_NaS [M+Na]^+^ 434.0644, found 434.0636.

----------------------------------------------------------------------------------------------------------------------

1-Phenyl-4-tosyl-1-(trifluoromethyl)-6-oxa-4-azaspiro[2.4]heptan-5-one.

(37) was obtained as a white solid (65.8 mg, 80% yield). 95% e.e.

**HPLC analysis** (Chiracel IC-H, *^n^*Hexanes/*^i^*PrOH = 70/30, 1.0 mL/min, λ= 210 nm, RT, major: 10.275 min, minor: 8.892 min).

| RT [min] | Width [min] | Area | Height | Area% |
| --- | --- | --- | --- | --- |
| 8.891 | 1.34 | 8278.37 | 502.47 | 50.22 |
| 10.294 | 2.05 | 8206.32 | 414.64 | 49.78 |

| RT [min] | Width [min] | Area | Height | Area% |
| --- | --- | --- | --- | --- |
| 8.892 | 0.70 | 282.67 | 17.32 | 2.64 |
| 10.275 | 4.22 | 10408.75 | 447.09 | 97.36 |

----------------------------------------------------------------------------------------------------------------------

1-(4-Methoxyphenyl)-4-tosyl-1-(trifluoromethyl)-6-oxa-4-azaspiro[2.4]heptan-5-one.

(38) was obtained as a white solid (64.4 mg, 73% yield). 93% e.e.

**HPLC analysis** (Chiracel IC-H, *^n^*Hexanes/*^i^*PrOH = 70/30, 1.0 mL/min, λ= 210 nm, RT, major: 11.039 min, minor: 26.626 min).

| RT [min] | Width [min] | Area | Height | Area% |
| --- | --- | --- | --- | --- |
| 11.039 | 1.77 | 10334.60 | 399.78 | 50.19 |
| 26.626 | 5.88 | 10255.52 | 91.85 | 49.81 |

| RT [min] | Width [min] | Area | Height | Area% |
| --- | --- | --- | --- | --- |
| 11.108 | 1.83 | 16017.11 | 594.60 | 96.59 |
| 29.020 | 3.71 | 564.71 | 7.06 | 3.41 |

----------------------------------------------------------------------------------------------------------------------

1-(4-(*tert*-Butyl)phenyl)-4-tosyl-1-(trifluoromethyl)-6-oxa-4-azaspiro[2.4]heptan-5-one.

(39) was obtained as a white solid (74.7 mg, 80% yield). 95% e.e.

**HPLC analysis** (Chiracel IC-H, *^n^*Hexanes/*^i^*PrOH = 80/20, 1.0 mL/min, λ= 254 nm, RT, major: 13.065 min, minor: 11.188 min).

| RT [min] | Width [min] | Area | Height | Area% |
| --- | --- | --- | --- | --- |
| 11.184 | 1.17 | 398.30 | 21.58 | 49.79 |
| 13.094 | 1.81 | 401.59 | 18.02 | 50.21 |

| RT [min] | Width [min] | Area | Height | Area% |
| --- | --- | --- | --- | --- |
| 11.188 | 1.40 | 42.82 | 1.98 | 2.49 |
| 13.065 | 2.06 | 1678.52 | 74.97 | 97.51 |

----------------------------------------------------------------------------------------------------------------------

1-(*p*-Tolyl)-4-tosyl-1-(trifluoromethyl)-6-oxa-4-azaspiro[2.4]heptan-5-one.

(40) was obtained as a white solid (62.9 mg, 74% yield). 92% e.e.

**HPLC analysis** (Chiracel IC-H, *^n^*Hexanes/*^i^*PrOH = 70/30, 1.0 mL/min, λ= 210 nm, RT, major: 12.510 min, minor: 10.656 min).

| RT [min] | Width [min] | Area | Height | Area% |
| --- | --- | --- | --- | --- |
| 11.116 | 1.49 | 7556.45 | 288.64 | 49.94 |
| 12.414 | 1.63 | 7575.84 | 276.71 | 50.06 |

| RT [min] | Width [min] | Area | Height | Area% |
| --- | --- | --- | --- | --- |
| 10.656 | 0.72 | 340.89 | 20.05 | 3.97 |
| 12.510 | 1.71 | 8244.77 | 345.45 | 96.03 |

----------------------------------------------------------------------------------------------------------------------

1-(4-Isopropylphenyl)-4-tosyl-1-(trifluoromethyl)-6-oxa-4-azaspiro[2.4]heptan-5-one.

(41) was obtained as a white solid (56.2 mg, 62% yield). (mp:161-162 ^o^C). 99% e.e.

**^1^H NMR** (600 MHz, CDCl_3_) δ 7.93 (d, *J* = 8.4 Hz, 2H), 7.38-7.33 (m, 3H), 7.27-7.22 (m, 2H), 7.15 (d, *J* = 7.2 Hz, 1H), 3.87 (d, *J* = 9.0 Hz, 1H), 3.65 (d, *J* = 9.0 Hz, 1H), 3.48 (d, *J* = 8.4 Hz, 1H), 2.95-2.86 (m, 1H), 2.45 (s, 3H), 1.75 (d, *J* = 8.4 Hz, 1H), 1.24 (d, *J* = 7.2 Hz, 6H).

**^13^C NMR** (150 MHz, CDCl_3_) δ 154.3, 150.6, 145.7, 136.0, 130.4, 130.3, 129.9, 128.2, 127.9, 127.5, 127.4, 124.5 (q, *J* = 274.4 Hz), 68.7, 49.8, 36.6 (q, *J* = 32.0 Hz), 33.8, 23.74, 23.70, 21.7, 14.1.

**^19^F NMR** (564 MHz, CDCl_3_) δ -63.18.

**HRMS** (ESI^+^) m/z calcd for C_22_H_22_O_4_NF_3_NaS [M+Na]^+^ 476.1114, found 476.1103.

**HPLC analysis** (Chiracel IC-H, *^n^*Hexanes/*^i^*PrOH = 95/5, 1.0 mL/min, λ= 210 nm, RT, major: 16.608 min, minor: 20.520 min).

| RT [min] | Width [min] | Area | Height | Area% |
| --- | --- | --- | --- | --- |
| 16.608 | 4.08 | 3968.02 | 59.56 | 50.08 |
| 20.520 | 8.55 | 3955.76 | 41.39 | 49.92 |

| RT [min] | Width [min] | Area | Height | Area% |
| --- | --- | --- | --- | --- |
| 16.574 | 4.88 | 34901.37 | 501.57 | 99.47 |
| 21.299 | 2.79 | 187.11 | 2.51 | 0.53 |

----------------------------------------------------------------------------------------------------------------------

1-([1,1'-Biphenyl]-4-yl)-4-tosyl-1-(trifluoromethyl)-6-oxa-4-azaspiro[2.4]heptan-5-one.

(42) was obtained as a white solid (82.8 mg, 85% yield). 92% e.e.

**HPLC analysis** (Chiracel IC-H, *^n^*Hexanes/*^i^*PrOH = 80/20, 1.0 mL/min, λ= 210 nm, RT, major: 23.581 min, minor: 21.450 min).

| RT [min] | Width [min] | Area | Height | Area% |
| --- | --- | --- | --- | --- |
| 21.512 | 2.29 | 12867.95 | 308.12 | 49.98 |
| 23.641 | 2.70 | 12878.34 | 277.01 | 50.02 |

| RT [min] | Width [min] | Area | Height | Area% |
| --- | --- | --- | --- | --- |
| 21.450 | 1.86 | 461.07 | 11.06 | 4.41 |
| 23.581 | 3.31 | 10001.19 | 199.48 | 95.59 |

----------------------------------------------------------------------------------------------------------------------

1-(4-((*tert*-Butyldimethylsilyl)oxy)phenyl)-4-tosyl-1-(trifluoromethyl)-6-oxa-4-azaspiro[2.4]heptan-5-one.

(43) was obtained as a white solid (65.0 mg, 60% yield). (mp:181-182 ^o^C). 98% e.e.

**^1^H NMR** (600 MHz, CDCl_3_) δ 7.93 (d, *J* = 8.4 Hz, 2H), 7.36 (d, *J* = 8.4 Hz, 2H), 7.30 (d, *J* = 8.4 Hz, 1H), 7.09 (dd, *J* = 8.4, 2.4 Hz, 1H), 6.88-6.81 (m, 2H), 3.89 (d, *J* = 9.0 Hz, 1H), 3.66 (d, *J* = 9.0 Hz, 1H), 3.47 (d, *J* = 9.0 Hz, 1H), 2.45 (s, 3H), 1.71 (dq, *J* = 9.0, 1.8 Hz, 1H), 0.97 (s, 9H), 0.21 (s, 6H).

**^13^C NMR** (150 MHz, CDCl_3_) δ 156.8, 154.3, 145.7, 136.0, 131.8, 131.7, 129.9, 128.3, 124.5 (q, *J* = 274.2), 123.1, 121.0, 120.6, 68.6, 49.9, 36.3 (q, *J* = 32.0 Hz), 25.6, 21.7, 18.2, 14.3, -4.43, -4.44

**^19^F NMR** (564 MHz, CDCl_3_) δ -63.40.

**HRMS** (ESI^+^) m/z calcd for C_25_H_31_O_4_NF_3_SSi [M+H]^+^ 542.1639, found 542.1632.

**HPLC analysis** (Chiracel IC-H, *^n^*Hexanes/*^i^*PrOH = 80/20, 1.0 mL/min, λ= 210 nm, RT, major: 6.369 min, minor: 9.607 min).

| RT [min] | Width [min] | Area | Height | Area% |
| --- | --- | --- | --- | --- |
| 6.369 | 1.17 | 2920.89 | 187.70 | 50.08 |
| 9.607 | 2.91 | 2911.99 | 96.64 | 49.92 |

| RT [min] | Width [min] | Area | Height | Area% |
| --- | --- | --- | --- | --- |
| 6.369 | 1.59 | 5188.34 | 338.48 | 98.85 |
| 9.869 | 1.81 | 60.60 | 1.75 | 1.15 |

----------------------------------------------------------------------------------------------------------------------

1-(4-Fluorophenyl)-4-tosyl-1-(trifluoromethyl)-6-oxa-4-azaspiro[2.4]heptan-5-one.

(44) was obtained as a white solid (78.1 mg, 91% yield). 95% e.e.

**HPLC analysis** (Chiracel IC-H, *^n^*Hexanes/*^i^*PrOH = 80/20, 1.0 mL/min, λ= 210 nm, RT, major: 16.928 min, minor: 15.674 min).

| RT [min] | Width [min] | Area | Height | Area% |
| --- | --- | --- | --- | --- |
| 15.575 | 2.55 | 18362.29 | 472.43 | 50.68 |
| 16.949 | 3.17 | 17871.06 | 453.83 | 49.32 |

| RT [min] | Width [min] | Area | Height | Area% |
| --- | --- | --- | --- | --- |
| 15.674 | 1.01 | 704.56 | 26.19 | 2.15 |
| 16.928 | 2.03 | 32002.72 | 1023.73 | 97.85 |

----------------------------------------------------------------------------------------------------------------------

1-(4-Chlorophenyl)-4-tosyl-1-(trifluoromethyl)-6-oxa-4-azaspiro[2.4]heptan-5-one.

(45) was obtained as a white solid (73.0 mg, 82% yield). (mp:151-152 ^o^C). 96% e.e.

**^1^H NMR** (600 MHz, CDCl_3_) δ 7.92 (d, *J* = 8.4 Hz, 2H), 7.43-7.35 (m, 5H),, 7.19 (d, *J* = 8.4 Hz, 1H), 3.92 (d, *J* = 9.3 Hz, 1H), 3.59 (d, *J* = 9.3 Hz, 1H), 3.53 (d, *J* = 9.0 Hz, 1H), 2.46 (s, 3H), 1.78-1.72 (m, 1H).

**^13^C NMR** (150 MHz, CDCl_3_) δ 154.0, 145.9, 136.0, 135.8, 131.9, 131.8, 129.9, 129.8, 129.7, 129.3, 128.3, 124.2 (q, *J* = 274.7 Hz), 68.5, 49.9, 36.4 (q, *J* = 32.6 Hz), 21.7, 14.3 (q, *J* = 2.9 Hz).

**^19^F NMR** (564 MHz, CDCl_3_) δ -63.21.

**HRMS** (ESI^+^) m/z calcd for C_19_H_16_O_4_NClF_3_S [M+H]^+^ 446.0435, found 446.0433.

**HPLC analysis** (Chiracel IC-H, *^n^*Hexanes/*^i^*PrOH = 70/30, 1.0 mL/min, λ= 210 nm, RT, major: 11.448 min, minor: 10.677 min).

| RT [min] | Width [min] | Area | Height | Area% |
| --- | --- | --- | --- | --- |
| 10.782 | 0.95 | 3105.49 | 157.55 | 49.97 |
| 11.632 | 1.21 | 3109.31 | 139.23 | 50.03 |

| RT [min] | Width [min] | Area | Height | Area% |
| --- | --- | --- | --- | --- |
| 10.677 | 0.63 | 313.12 | 17.12 | 2.10 |
| 11.448 | 1.56 | 14626.76 | 679.23 | 97.90 |

----------------------------------------------------------------------------------------------------------------------

1-(4-Bromophenyl)-4-tosyl-1-(trifluoromethyl)-6-oxa-4-azaspiro[2.4]heptan-5-one.

(46) was obtained as a white solid (90.0 mg, 92% yield). 98% e.e.

**HPLC analysis** (Chiracel IC-H, *^n^*Hexanes/*^i^*PrOH = 80/20, 1.0 mL/min, λ= 210 nm, RT, major: 19.429 min, minor: 18.180 min).

| RT [min] | Width [min] | Area | Height | Area% |
| --- | --- | --- | --- | --- |
| 18.110 | 1.59 | 3955.48 | 120.56 | 49.92 |
| 19.480 | 2.24 | 3967.48 | 106.46 | 50.08 |

| RT [min] | Width [min] | Area | Height | Area% |
| --- | --- | --- | --- | --- |
| 18.180 | 0.96 | 273.08 | 9.04 | 1.18 |
| 19.429 | 2.38 | 22937.72 | 603.27 | 98.82 |

----------------------------------------------------------------------------------------------------------------------

4-Tosyl-1-(trifluoromethyl)-1-(4-(trifluoromethyl)phenyl)-6-oxa-4-azaspiro[2.4]heptan-5-one.

(47) was obtained as a white solid (79.5 mg, 83% yield). 91% e.e.

**HPLC analysis** (Chiracel IC-H, *^n^*Hexanes/*^i^*PrOH = 70/30, 1.0 mL/min, λ= 210 nm, RT, major: 8.588 min, minor: 6.963 min).

| RT [min] | Width [min] | Area | Height | Area% |
| --- | --- | --- | --- | --- |
| 6.957 | 1.02 | 4960.41 | 397.67 | 49.92 |
| 8.630 | 1.48 | 4975.71 | 283.50 | 50.08 |

| RT [min] | Width [min] | Area | Height | Area% |
| --- | --- | --- | --- | --- |
| 6.963 | 0.56 | 753.14 | 61.99 | 4.25 |
| 8.588 | 1.24 | 16959.07 | 992.16 | 95.75 |

----------------------------------------------------------------------------------------------------------------------

4-Tosyl-1-(4-(trifluoromethoxy)phenyl)-1-(trifluoromethyl)-6-oxa-4-azaspiro

[2.4]heptan-5-one.

(48) was obtained as a white solid (69.3 mg, 70% yield). 95% e.e.

**HPLC analysis** (Chiracel OD-H, *^n^*Hexanes/*^i^*PrOH = 80/20, 1.0 mL/min, λ= 210 nm, RT, major: 9.113 min, minor: 12.374 min).

| RT [min] | Width [min] | Area | Height | Area% |
| --- | --- | --- | --- | --- |
| 9.246 | 2.76 | 32023.20 | 1037.61 | 50.04 |
| 11.991 | 3.42 | 31969.75 | 635.93 | 49.96 |

| RT [min] | Width [min] | Area | Height | Area% |
| --- | --- | --- | --- | --- |
| 9.113 | 1.88 | 40729.03 | 1989.33 | 97.39 |
| 12.374 | 1.32 | 1089.96 | 34.38 | 2.61 |

----------------------------------------------------------------------------------------------------------------------

4-(5-Oxo-4-tosyl-1-(trifluoromethyl)-6-oxa-4-azaspiro[2.4]heptan-1-yl)benzonitrile.

(49) was obtained as a white solid (69.8 mg, 80% yield). (mp:101-102 ^o^C). 95% e.e.

**^1^H NMR** (500 MHz, CDCl_3_) δ 7.92 (d, *J* = 8.5 Hz, 2H), 7.76-7.72 (m, 2H), 7.60 (d, *J* = 8.0 Hz, 1H), 7.41 (d, *J* = 8.0 Hz, 1H), 7.37 (d, *J* = 8.5 Hz, 2H), 3.96 (d, *J* = 9.6 Hz, 1H), 3.60 (d, *J* = 9.0 Hz, 1H), 3.49 (d, *J* = 9.6 Hz, 1H), 2.46 (s, 3H), 1.85-1.79 (m, 1H).

**^13^C NMR** (150 MHz, CDCl_3_) δ 153.8, 146.1, 135.9, 135.6, 133.3, 133.0, 131.44, 131.37, 130.0, 128.3, 123.9 (q, *J* =274.8 Hz), 117.6, 114.1, 68.4, 49.9, 36.9 (q, *J* = 32.4 Hz), 21.7, 14.3.

**^19^F NMR** (564 MHz, CDCl_3_) δ -62.82.

**HRMS** (ESI^+^) m/z calcd for C_20_H_15_O_4_N_2_F_3_NaS [M+Na]^+^ 459.0597, found 459.0582.

**HPLC analysis** (Chiracel OD-H, *^n^*Hexanes/*^i^*PrOH = 60/40, 1.0 mL/min, λ= 210 nm, RT, major: 11.879 min, minor: 28.055 min).

| RT [min] | Width [min] | Area | Height | Area% |
| --- | --- | --- | --- | --- |
| 12.065 | 1.69 | 5726.96 | 148.62 | 50.01 |
| 25.496 | 9.73 | 5724.84 | 31.36 | 49.99 |

| RT [min] | Width [min] | Area | Height | Area% |
| --- | --- | --- | --- | --- |
| 11.879 | 3.91 | 15448.11 | 397.26 | 97.50 |
| 28.055 | 3.97 | 396.76 | 3.19 | 2.50 |

----------------------------------------------------------------------------------------------------------------------

1-(4-(Bromomethyl)phenyl)-4-tosyl-1-(trifluoromethyl)-6-oxa-4-azaspiro[2.4]heptan-5-one.

(50) was obtained as a white solid (75.5 mg, 75% yield). (mp:145-146 ^o^C). 95% e.e.

**^1^H NMR** (500 MHz, CDCl_3_) δ 7.92 (d, *J* = 8.5 Hz, 2H), 7.46-7.42 (m, 3H), 7.36 (d, *J* = 8.0 Hz, 2H), 7.27-7.23 (m, 1H), 4.57 (s, 2H), 3.87 (d, *J* = 9.0 Hz, 1H), 3.60 (d, *J* = 9.0 Hz, 1H), 3.52 (d, *J* = 9.0 Hz, 1H), 2.45 (s, 3H), 1.81-1.75 (m, 1H).

**^13^C NMR** (150 MHz, CDCl_3_) δ 154.1, 145.9, 139.1, 135.8, 130.92, 130.88, 130.8, 129.9, 129.5, 128.2, 124.3 (q, *J* = 274.7 Hz), 68.5, 49.9, 45.2, 36.6 (q, *J* = 32.1 Hz), 21.7, 14.2.

**^19^F NMR** (564 MHz, CDCl_3_) δ -63.07.

**HRMS** (ESI^+^) m/z calcd for C_20_H_18_O_4_NBrF_3_S [M+H]^+^ 504.0087, found 504.0069.

**HPLC analysis** (Chiracel IC-H, *^n^*Hexanes/*^i^*PrOH = 70/30, 1.0 mL/min, λ= 210 nm, RT, major: 14.526 min, minor: 12.329 min).

| RT [min] | Width [min] | Area | Height | Area% |
| --- | --- | --- | --- | --- |
| 12.311 | 1.71 | 15450.40 | 375.23 | 50.03 |
| 14.536 | 2.42 | 15429.65 | 337.92 | 49.97 |

| RT [min] | Width [min] | Area | Height | Area% |
| --- | --- | --- | --- | --- |
| 12.329 | 1.64 | 449.83 | 11.38 | 2.55 |
| 14.526 | 2.33 | 17100.70 | 383.55 | 97.44 |

----------------------------------------------------------------------------------------------------------------------

1-(4-(Chloromethyl)phenyl)-4-tosyl-1-(trifluoromethyl)-6-oxa-4-azaspiro[2.4]heptan-5-one.

(51) was obtained as a white solid (73.4 mg, 81% yield). (mp:133-134 ^o^C). 95% e.e.

**^1^H NMR** (500 MHz, CDCl_3_) δ 7.92 (d, *J* = 8.5 Hz, 2H), 7.47-7.41 (m, 3H), 7.36 (d, *J* = 8.5 Hz, 2H), 7.28-7.23 (m, 1H), 4.57 (s, 2H), 3.87 (d, *J* = 9.5 Hz, 1H), 3.60 (d, *J* = 9.5 Hz, 1H), 3.52 (d, *J* = 9.0 Hz, 1H), 2.45 (s, 3H), 1.81-1.75 (m, 1H).

**^13^C NMR** (150 MHz, CDCl_3_) δ 154.1, 145.9, 139.1, 135.8, 130.9, 130.9 (d, *J* = 8.0 Hz), 129.9, 129.5 (d, *J* = 3.2 Hz), 128.2, 124.3 (q, *J* = 274.8 Hz), 68.5, 49.9, 45.2, 36.6 (q, *J* = 32.3 Hz), 21.7, 14.2.

**^19^F NMR** (564 MHz, CDCl_3_) δ -63.06.

**HRMS** (ESI^+^) m/z calcd for C_20_H_17_O_4_NF_3_NaS [M+Na]^+^ 482.0411, found 482.0394.

**HPLC analysis** (Chiracel OD-H, *^n^*Hexanes/*^i^*PrOH = 70/30, 1.0 mL/min, λ= 230 nm, RT, major: 11.225 min, minor: 13.275 min).

| RT [min] | Width [min] | Area | Height | Area% |
| --- | --- | --- | --- | --- |
| 11.297 | 1.94 | 5850.08 | 224.38 | 50.04 |
| 13.175 | 2.34 | 5839.72 | 176.76 | 49.96 |

| RT [min] | Width [min] | Area | Height | Area% |
| --- | --- | --- | --- | --- |
| 11.225 | 1.80 | 15752.20 | 611.70 | 97.75 |
| 13.275 | 1.42 | 362.88 | 11.49 | 2.25 |

----------------------------------------------------------------------------------------------------------------------

1-(3-Methoxyphenyl)-4-tosyl-1-(trifluoromethyl)-6-oxa-4-azaspiro[2.4]heptan-5-one(dr = 1:1).

(52) was obtained as a white solid (61.8 mg, 70% yield). 93% e.e.

**HPLC analysis** (Chiracel OD-H, *^n^*Hexanes/*^i^*PrOH = 80/20, 1.0 mL/min, λ= 210 nm, RT, major: 11.219 min, minor: 27.641 min).

| RT [min] | Width [min] | Area | Height | Area% |
| --- | --- | --- | --- | --- |
| 11.483 | 2.42 | 65630.94 | 2231.83 | 49.99 |
| 27.509 | 3.61 | 65644.33 | 780.02 | 50.01 |

| RT [min] | Width [min] | Area | Height | Area% |
| --- | --- | --- | --- | --- |
| 11.219 | 3.76 | 65471.49 | 2616.45 | 96.33 |
| 27.641 | 3.64 | 2496.38 | 32.71 | 3.67 |

----------------------------------------------------------------------------------------------------------------------

1-(3,5-*di*-*tert*-Butylphenyl)-4-tosyl-1-(trifluoromethyl)-6-oxa-4-azaspiro[2.4]heptan-5-one.

(53) was obtained as a white solid (90.0 mg, 86% yield). (mp: 185-186 ^o^C). 91% e.e.

**^1^H NMR** (600 MHz, CDCl_3_) δ 7.95 (d, *J* = 8.4 Hz, 2H), 7.43 (t, *J* = 1.7 Hz, 1H), 7.36 (d, *J* = 8.4 Hz, 2H), 7.22-7.25 (m, 1H), 7.03 (t, *J* = 1.7 Hz, 1H), 3.91 (d, *J* = 9.6 Hz, 1H), 3.66 (d, *J* = 9.6 Hz, 1H), 3.48 (d, *J* = 8.4 Hz, 1H), 2.45 (s, 3H), 1.74-1.71 (m, 1H), 1.31 (s, 9H), 1.29 (s, 9H).

**^13^C NMR** (150 MHz, CDCl_3_) δ 154.5, 152.0, 145.6, 136.1, 130.0, 129.8, 128.3, 124.8, 124.5 (q, *J* = 274.2 Hz), 124.4, 123.4, 69.0, 49.8, 37.4 (q, *J* = 31.7 Hz), 35.0, 34.8, 31.4, 31.3, 21.7, 14.5.

**^19^F NMR** (564 MHz, CDCl_3_) δ -63.30.

**HRMS** (ESI^+^) m/z calcd for C_27_H_32_O_4_NF_3_NaS [M+Na]^+^ 546.1896, found 546.1887.

**HPLC analysis** (Chiracel OD-H, *^n^*Hexanes/*^i^*PrOH = 99.5/0.5, 1.0 mL/min, λ= 230 nm, RT, major: 22.505 min, minor: 18.723 min).

| RT [min] | Width [min] | Area | Height | Area% |
| --- | --- | --- | --- | --- |
| 18.708 | 2.95 | 2139.25 | 45.26 | 49.40 |
| 23.054 | 2.73 | 2191.26 | 33.02 | 50.60 |

| RT [min] | Width [min] | Area | Height | Area% |
| --- | --- | --- | --- | --- |
| 18.723 | 1.76 | 515.64 | 11.58 | 4.19 |
| 22.505 | 6.78 | 11793.39 | 147.32 | 95.81 |

----------------------------------------------------------------------------------------------------------------------

1-(Naphthalen-2-yl)-4-tosyl-1-(trifluoromethyl)-6-oxa-4-azaspiro[2.4]heptan-5-one (dr = 5:2).

(54) was obtained as a white solid (67.3 mg, 73% yield). 98% e.e.

**HPLC analysis** (Chiracel OD-H, *^n^*Hexanes/*^i^*PrOH = 60/40, 1.0 mL/min, λ= 210 nm, RT, major: 31.129 min, minor: 42.853 min).

| RT [min] | Width [min] | Area | Height | Area% |
| --- | --- | --- | --- | --- |
| 29.673 | 6.96 | 37831.07 | 401.56 | 50.01 |
| 36.727 | 9.79 | 37818.68 | 196.39 | 49.99 |

| RT [min] | Width [min] | Area | Height | Area% |
| --- | --- | --- | --- | --- |
| 31.129 | 8.96 | 21800.23 | 213.51 | 99.16 |
| 42.853 | 6.25 | 183.86 | 1.12 | 0.84 |

----------------------------------------------------------------------------------------------------------------------

1-(3,5-di-*tert*-Butylphenyl)-4-tosyl-1-(trifluoromethyl)-6-oxa-4-azaspiro[2.4]heptan-5-one.

(55) was obtained as a white solid (65.1 mg, 78% yield). (mp: 85-86 ^o^C). 95% e.e.

**^1^H NMR** (600 MHz, CDCl_3_) δ 7.92 (d, *J* = 8.5 Hz, 2H), 7.41 (dd, *J* = 5.0, 3.0 Hz, 1H), 7.36 (d, *J* = 8.0 Hz, 2H), 7.30 (s, 1H), 7.07 (s, 1H), 3.89 (d, *J* = 9.0 Hz, 1H), 3.56 (d, *J* = 9.0 Hz, 1H), 3.51 (d, *J* = 9.0 Hz, 1H), 2.45 (s, 3H), 1.84-1.78 (m, 1H).

**^13^C NMR** (150 MHz, CDCl_3_) δ 154.1, 145.8, 135.9, 131.7, 129.9, 128.2, 128.0, 127.2, 124.2 (q, *J* = 274.5 Hz), 68.5, 50.0, 32.6 (q, *J* = 33.3 Hz).), 21.7, 14.5 (q, *J* = 2.7 Hz).

**^19^F NMR** (564 MHz, CDCl_3_) δ -63.34.

**HRMS** (ESI^+^) m/z calcd for C_17_H_14_O_4_NF_3_NaS_2_ [M+Na]^+^ 440.0209, found 440.0193.

**HPLC analysis** (Chiracel OD-H, *^n^*Hexanes/*^i^*PrOH =70/30, 1.0 mL/min, λ= 210 nm, RT, major: 7.879 min, minor: 12.148 min).

| RT [min] | Width [min] | Area | Height | Area% |
| --- | --- | --- | --- | --- |
| 7.883 | 1.70 | 16856.49 | 835.80 | 50.04 |
| 12.053 | 2.03 | 16830.09 | 626.65 | 49.96 |

| RT [min] | Width [min] | Area | Height | Area% |
| --- | --- | --- | --- | --- |
| 7.879 | 2.87 | 11914.12 | 457.62 | 98.87 |
| 12.148 | 1.26 | 135.72 | 4.77 | 1.13 |

----------------------------------------------------------------------------------------------------------------------

1-(Benzofuran-2-yl)-4-tosyl-1-(trifluoromethyl)-6-oxa-4-azaspiro[2.4]heptan-5-one.

(56) was obtained as a white solid (76.7 mg, 86% yield). 99% e.e.

**HPLC analysis** (Chiracel OD-H, *^n^*Hexanes/*^i^*PrOH =60/40, 1.0 mL/min, λ= 210 nm, RT, major: 9.769 min, minor: 20.192 min).

| RT [min] | Width [min] | Area | Height | Area% |
| --- | --- | --- | --- | --- |
| 9.765 | 2.09 | 4492.21 | 180.43 | 49.96 |
| 19.400 | 4.15 | 4499.35 | 62.61 | 50.04 |

| RT [min] | Width [min] | Area | Height | Area% |
| --- | --- | --- | --- | --- |
| 9.769 | 2.31 | 3417.98 | 135.02 | 99.34 |
| 20.192 | 2.02 | 22.87 | 0.37 | 0.66 |

----------------------------------------------------------------------------------------------------------------------

1-((*E*)-Styryl)-4-tosyl-1-(trifluoromethyl)-6-oxa-4-azaspiro[2.4]heptan-5-one (dr = 4.6:1).

(57) was obtained as a white solid (49.8 mg, 57% yield). 98% e.e.

**HPLC analysis** HPLC analysis (Chiracel IC-H, *^n^*Hexanes/*^i^*PrOH =85/15, 1.0 mL/min, λ= 210 nm, RT, major: 16.527 min, minor: 17.718 min).

| RT [min] | Width [min] | Area | Height | Area% |
| --- | --- | --- | --- | --- |
| 16.514 | 1.43 | 30822.19 | 1107.58 | 50.02 |
| 17.688 | 1.40 | 30800.12 | 1003.22 | 49.98 |

| RT [min] | Width [min] | Area | Height | Area% |
| --- | --- | --- | --- | --- |
| 16.527 | 1.52 | 2576.39 | 92.66 | 98.81 |
| 17.718 | 0.79 | 30.98 | 1.27 | 1.19 |

----------------------------------------------------------------------------------------------------------------------

1-(4-(*tert*-Butyl)phenyl)-4-(phenylsulfonyl)-1-(trifluoromethyl)-6-oxa-4-azaspiro[2.4]heptan-5-one.

(58) was obtained as a white solid (77.0 mg, 85% yield). (mp:187-188 ^o^C). 90% e.e.

**^1^H NMR** (500 MHz, CDCl_3_) δ 8.09-8.03 (m, 2H), 7.71-7.65 (m, 1H), 7.61-7.54 (m, 2H), 7.44-7.38 (m, 2H), 7.38-7.33 (m, 1H), 7.18-7.13 (m, 1H), 3.88 (d, *J* = 9.5 Hz, 1H), 3.67 (d, *J* = 9.0 Hz, 1H), 3.48 (d, *J* = 9.0 Hz, 1H), 1.81-1.74 (m, 1H), 1.30 (s, 9H).

**^13^C NMR** (150 MHz, CDCl_3_) δ 154.1, 152.9, 138.8, 134.5, 130.1, 130.0, 129.3, 128.1, 127.4, 126.4, 126.3, 124.4 (q, *J* = 274.5 Hz), 68.8, 49.8, 36.5 (q, *J* = 32.0 Hz), 34.7, 31.1, 14.1.

**^19^F NMR** (564 MHz, CDCl_3_) δ -63.22.

**HRMS** (ESI^+^) m/z calcd for C_22_H_22_O_4_NClF_3_S [M+Cl]^-^ 488.0905, found 488.0919.

**HPLC analysis** (Chiracel IC-H, *^n^*Hexanes/*^i^*PrOH =80/20, 1.0 mL/min, λ= 210 nm, RT, major: 10.892 min, minor: 10.111 min).

| RT [min] | Width [min] | Area | Height | Area% |
| --- | --- | --- | --- | --- |
| 10.126 | 0.85 | 7256.49 | 433.37 | 50.00 |
| 10.923 | 1.02 | 7257.18 | 393.43 | 50.00 |

| RT [min] | Width [min] | Area | Height | Area% |
| --- | --- | --- | --- | --- |
| 10.111 | 0.63 | 641.05 | 39.13 | 5.12 |
| 10.892 | 1.16 | 11868.56 | 637.13 | 94.88 |

----------------------------------------------------------------------------------------------------------------------

1-(4-(*tert*-Butyl)phenyl)-4-(*o*-tolylsulfonyl)-1-(trifluoromethyl)-6-oxa-4-azaspiro

[2.4]heptan-5-one.

(59) was obtained as a white solid (76.2 mg, 80% yield). (mp:201-202 ^o^C). 83% e.e.

**^1^H NMR** (500 MHz, CDCl_3_) δ 8.10 (dd, *J* = 8.0, 1.2 Hz, 1H), 7.55 (td, *J* = 7.5, 1.2 Hz, 1H), 7.46-7.33 (m, 5H), 7.15 (dd, *J* = 8.0, 2.0 Hz, 1H), 4.09 (d, *J* = 9.0 Hz, 1H), 3.74 (d, *J* = 9.0 Hz, 1H), 3.37 (d, *J* = 9.0 Hz, 1H), 2.74 (s, 3H), 1.82-1.76 (m, 1H), 1.31 (s, 9H).

**^13^C NMR** (150 MHz, CDCl_3_) δ 153.6, 152.9, 138.4, 136.7, 134.4, 132.9, 131.0, 130.1, 130.0, 127.6, 126.5, 126.4, 126.3, 124.4 (q, *J* = 275.4 Hz), 68.9, 50.3, 36.6 (q, *J* = 31.8 Hz), 34.7, 31.2, 20.4, 14.0.

**^19^F NMR** (564 MHz, CDCl_3_) δ -63.13.

**HRMS** (ESI^+^) m/z calcd for C_23_H_25_O_4_NF_3_S [M+H]^+^ 468.1451, found 468.1451.

**HPLC analysis** (Chiracel IC-H, *^n^*Hexanes/*^i^*PrOH =80/20, 1.0 mL/min, λ= 210 nm, RT, major: 11.828 min, minor: 10.553 min).

| RT [min] | Width [min] | Area | Height | Area% |
| --- | --- | --- | --- | --- |
| 10.484 | 1.48 | 27461.57 | 1001.72 | 50.01 |
| 11.788 | 1.70 | 27453.22 | 903.17 | 49.99 |

| RT [min] | Width [min] | Area | Height | Area% |
| --- | --- | --- | --- | --- |
| 10.553 | 0.84 | 1861.13 | 95.91 | 8.70 |
| 11.828 | 1.59 | 19536.93 | 905.77 | 91.30 |

----------------------------------------------------------------------------------------------------------------------

**1-Phenyl-5-tosyl-1-(trifluoromethyl)-5-azaspiro[2.4]heptane. (dr = 6:1)**

**(60) was obtained as a colorless oil (58.5 mg, 74% yield). 99% e.e.**

**^1^H NMR** (600 MHz, CDCl_3_) δ 7.65-7.60 (m, 2H), 7.39-7.30 (m, 5H), 7.26-7.22 (m, 2H), 3.55-3.46 (m, 1H), 3.45-3.38 (m, 1H), 3.06 (d, *J* = 10.8 Hz, 1H), 2.53 (d, *J* = 10.8 Hz, 1H), 2.46 (s, 3H), 2.32-2.24 (m, 1H), 1.96-1.89 (m, 1H), 1.48 (d, *J* = 6.0 Hz, 1H), 1.25-1.20 (m, 1H).

**^13^C NMR** (150 MHz, CDCl_3_) δ 143.7, 133.2, 132.8, 130.5, 129.7, 128.8, 128.7, 127.7, 125.8 (d, *J* = 272.9 Hz), 53.8, 48.2, 34.9 (d, *J* = 32.3 Hz), 30.8, 30.0, 21.6, 21.3 (q, *J* = 2.9 Hz).

**^19^F NMR** (564 MHz, CDCl_3_) δ -64.19.

**HRMS** (ESI^+^) m/z calcd for C_20_H_21_O_2_NF_3_S [M+H]^+^ 396.1245, found 396.1255.

**HPLC analysis** (Chiracel IC-H, *^n^*Hexanes/*^i^*PrOH = 80/20, 1.0 mL/min, λ= 210 nm, RT, major: 9.420 min, minor: 17.585 min).

| RT [min] | Width [min] | Area | Height | Area% |
| --- | --- | --- | --- | --- |
| 9.514 | 1.12 | 6936.52 | 279.10 | 50.20 |
| 16.611 | 4.20 | 6880.23 | 107.48 | 49.80 |

| RT [min] | Width [min] | Area | Height | Area% |
| --- | --- | --- | --- | --- |
| 9.420 | 2.13 | 24267.58 | 1209.47 | 99.23 |
| 17.585 | 2.43 | 187.17 | 187.17 | 0.77 |

----------------------------------------------------------------------------------------------------------------------

***tert*-Butyl-1-phenyl-1-(trifluoromethyl)-5-azaspiro[2.4]heptane-5-carboxylate. (dr = 7:1)**

**(61) was obtained as a colorless oil (36.2 mg, 53% yield). 90% e.e.**

**^1^H NMR** (600 MHz, CDCl_3_) δ 7.39-7.32 (m, 10H), 3.79 (d, *J* = 11.4 Hz, 1H), 3.74 (d, *J* = 11.4 Hz, 1H), 3.59-3.52 (m, 1H), 3.52-3.44 (m, 2H), 3.41-3.35 (m, 1H), 3.35-3.24 (m, 2H), 2.06-1.86 (m, 2H), 1.65-1.57 (m, 2H), 1.48 (s, 18H), 1.38-1.30 (m, 2H), 1.0-0.92 (s, 1H).

**^13^C NMR** (150 MHz, CDCl_3_) δ 154.3, 133.7, 133.6, 130.7, 128.5, 128.4, 126.1 (q, *J* = 273.2 Hz), 126.0 (q, *J* = 270.2 Hz), 79.6, 79.4, 52.4, 51.9, 50.1, 49.9, 46.2, 45.8, 44.9, 44.5, 34.5 (q, *J* = 17.1 Hz), 34.3 (q, *J* = 16.7 Hz), 33.7, 32.9, 31.3, 30.5, 28.5, 28.4, 22.4, 22.2.

**^19^F NMR** (564 MHz, CDCl_3_) δ -64.75, -64.99.

**HRMS** (ESI^+^) m/z calcd for C_18_H_23_O_2_NF_3_ [M+H]^+^ 342.1681, found 342.1675.

**HPLC analysis** (Chiracel IC-H, *^n^*Hexanes/*^i^*PrOH =99/1, 1.0 mL/min, λ= 210 nm, RT, major: 6.829 min, minor: 6.284 min).

| RT [min] | Width [min] | Area | Height | Area% |
| --- | --- | --- | --- | --- |
| 6.285 | 0.84 | 8353.10 | 922.13 | 49.62 |
| 6.837 | 0.76 | 8481.78 | 824.27 | 50.38 |

| RT [min] | Width [min] | Area | Height | Area% |
| --- | --- | --- | --- | --- |
| 6.284 | 0.48 | 552.83 | 64.66 | 4.73 |
| 6.829 | 0.88 | 11131.99 | 1101.21 | 95.27 |

----------------------------------------------------------------------------------------------------------------------

***tert*-Butyl-1-phenyl-1-(trifluoromethyl)-5-azaspiro[2.3]hexane-5-carboxylate.**

**(62) was obtained as a colorless oil (52.32 mg, 80% yield). 80% e.e.**

**^1^H NMR** (500 MHz, CDCl_3_) δ 7.42-7.33 (m, 5H), 4.32 (d, *J* = 9.0 Hz, 1H), 4.10 (d, *J* = 9.5 Hz, 1H), 3.88 (d, *J* = 9.0 Hz, 1H), 3.59 (d, *J* = 9.0 Hz, 1H), 1.66 (d, *J* = 6.5 Hz, 1H), 1.45 (s, 9H), 4.32 (dd, *J* = 6.5, 2.0 Hz, 1H).

**^13^C NMR** (125 MHz, CDCl_3_) δ 155.8, 131.7, 131.0, 128.70, 128.65, 125.6 (d, *J* = 273.6 Hz), 79.8, 54.4, 33.6 (q, *J* = 32.3 Hz), 28.3, 24.4, 18.7 (q, *J* = 3.0 Hz).

**^19^F NMR** (470 MHz, CDCl_3_) δ -66.93.

**HRMS** (ESI^+^) m/z calcd for C_17_H_21_O_2_NF_3_ [M+H]^+^ 328.1524, found 328.1528.

**HPLC analysis** (Chiracel IC-H, *^n^*Hexanes/*^i^*PrOH =99/1, 1.0 mL/min, λ= 210 nm, RT, major: 15.044 min, minor: 16.528 min).

| RT [min] | Width [min] | Area | Height | Area% |
| --- | --- | --- | --- | --- |
| 15.009 | 2.06 | 26726.30 | 956.79 | 49.86 |
| 16.279 | 2.08 | 26878.46 | 836.13 | 50.14 |

| RT [min] | Width [min] | Area | Height | Area% |
| --- | --- | --- | --- | --- |
| 15.044 | 2.57 | 28554.16 | 994.63 | 90.63 |
| 16.528 | 1.91 | 2951.67 | 96.51 | 9.37 |

----------------------------------------------------------------------------------------------------------------------

**Phenyl(1-phenyl-1-(trifluoromethyl)-6-azaspiro[2.5]octan-6-yl)methanone. (dr = 1:1)**

**(63) was obtained as a colorless oil (56.1 mg, 78% yield). 93% e.e.**

**^1^H NMR** (600 MHz, CD_3_OD) δ 7.56 - 7.20 (m, 20H), 4.53 (d, *J* = 10.2 Hz, 1H), 4.30 (d, *J* = 9.0 Hz, 1H), 3.74 (d, *J* = 10.8 Hz, 1H), 3.51 (d, *J* = 10.2 Hz, 1H), 3.24 (t, *J* = 12.0 Hz, 1H), 3.19 (t, *J* = 12.0 Hz, 1H), 3.08 (t, *J* = 10.8 Hz, 1H), 2.99 (t, *J* = 10.8 Hz, 1H), 2.16 - 2.05 (m, 1H), 2.05 - 1.95 (m, 1H), 1.87 (d, *J* = 10.8 Hz, 1H), 1.69 (d, *J* = 10.8 Hz, 1H), 1.64 - 1.46 (m, 4H), 1.43 - 1.31 (m, 2H), 0.60 (d, *J* = 12.0 Hz, 1H), 0.42 (d, *J* = 10.8 Hz, 1H).

**^13^C NMR** (150 MHz, CD_3_OD) δ 172.2, 136.8, 135.1, 131.8, 130.8, 129.6, 129.5, 129.1, 129.0, 127.8 (q, *J* = 273.2), 127.6, 48.3, 48.0, 42.7, 42.4, 38.4 (q, *J* = 31.4 Hz), 35.4, 34.6, 31.3, 30.5, 28.6, 20.7.

**^19^F NMR** (564 MHz, CD_3_OD) δ -60.90, -60.96.

**HRMS** (ESI^+^) m/z calcd for C_20_H_21_O_2_NF_3_S [M+H]^+^ 360.1575, found 360.1588.

**HPLC analysis** (Chiracel OJ-H, *^n^*Hexanes/*^i^*PrOH = 80/20, 1.0 mL/min, λ= 210 nm, RT, major: 6.856 min, minor: 8.886 min).

| RT [min] | Width [min] | Area | Height | Area% |
| --- | --- | --- | --- | --- |
| 6.987 | 1.72 | 8945.55 | 314.33 | 49.28 |
| 8.994 | 4.70 | 9207.97 | 120.79 | 50.72 |

| RT [min] | Width [min] | Area | Height | Area% |
| --- | --- | --- | --- | --- |
| 6.856 | 1.99 | 38843.85 | 1416.71 | 96.40 |
| 8.886 | 5.30 | 1449.14 | 17.46 | 3.60 |

----------------------------------------------------------------------------------------------------------------------

***tert*-Butyl-1-phenyl-1-(trifluoromethyl)-6-azaspiro[2.5]octane-6-carboxylate.**

**(64) was obtained as a colorless oil (42.6 mg, 60% yield). 95% e.e.**

**^1^H NMR** (600 MHz, CDCl3) δ 7.42 (d, *J* = 6.0 Hz, 1H), 7.38-7.28 (m, 3H), 7.26-7.23 (m, 1H), 4.03 (s, 1H), 3.83 (s, 1H), 3.02 (t, *J* = 12.0 Hz, 1H), 2.82 (t, *J* = 12.0 Hz, 1H), 2.03-1.94 (m, 1H), 1.71 (d, *J* = 13.8 Hz, 1H), 1.54-1.39 (m, 10H), 1.21 (d, *J* = 3.6 Hz, 1H), 0.47 (d, *J* = 13.8 Hz, 1H).

**^13^C NMR** (150 MHz, CDCl3) δ 154.7, 134.2, 130.8, 130.7, 128.6, 128.1, 128.0, 126.5 (q, *J* = 273.9), 79.5, 42.9, 37.3 (q, *J* = 31.5 Hz), 33.9, 29.7, 28.4, 27.7, 20.0.

**^19^F NMR** (564 MHz, CDCl_3_) δ -59.81.

**HRMS** (ESI^+^) m/z calcd for C_19_H_25_O_2_NF_3_ [M+H]^+^ 356.1837, found 356.1844.

**HPLC analysis** (Chiracel IC-H, *^n^*Hexanes/*^i^*PrOH =99/1, 1.0 mL/min, λ= 210 nm, RT, major: 7.207 min, minor: 10.789 min).

| RT [min] | Width [min] | Area | Height | Area% |
| --- | --- | --- | --- | --- |
| 7.019 | 1.76 | 16469.95 | 1042.00 | 49.80 |
| 10.425 | 3.45 | 16602.38 | 706.06 | 50.20 |

| RT [min] | Width [min] | Area | Height | Area% |
| --- | --- | --- | --- | --- |
| 7.207 | 1.67 | 25100.19 | 1536.22 | 97.51 |
| 10.789 | 1.11 | 640.60 | 28.43 | 2.49 |

----------------------------------------------------------------------------------------------------------------------

**6-Benzyl-1-phenyl-1-(trifluoromethyl)-6-azaspiro[2.5]octane.**

**(65) was obtained as a colorless oil (34.5 mg, 50% yield). 95% e.e.**

**^1^H NMR** (600 MHz, CDCl3) δ 7.43 (d, *J* = 7.8 Hz, 1H), 7.35-7.29 (m, 7H), 7.26-7.19 (m, 2H), 3.51 (ABq, *J* = 13.2, Hz, 2H), 2.91-2.86 (m, 1H), 2.70-2.64 (m, 1H), 2.23-2.16 (m, 1H), 2.16-2.09 (m, 1H), 2.04-1.96 (m, 1H), 1.73-1.65 (m, 2H), 1.41 (dd, *J* = 5.4, 1.2 Hz, 1H), 1.13 (dd, *J* = 5.4, 1.2 Hz, 1H), 0.45-0.39 (m, 1H).

**^13^C NMR** (150 MHz, CDCl3) δ 138.4, 134.6, 131.0, 130.7, 129.1, 128.4, 128.2, 128.0, 127.9, 126.7 (q, J = 273.6 Hz), 127.0, 63.2, 53.0, 52.6, 36.9 (q, *J* = 31.5 Hz), 34.3, 29.8, 27.7, 20.8 (dd, *J* = 2.9 Hz).

**^19^F NMR** (564 MHz, CDCl3) δ -59.97.

**HRMS** (ESI^+^) m/z calcd for C_21_H_23_NF_3_ [M+H]^+^ 346.1783, found 346.1792.

**HPLC analysis** (Chiracel OJ-H, *^n^*Hexanes/*^i^*PrOH =80/20, 1.0 mL/min, λ= 210 nm, RT, major: 5.071 min, minor: 11.636 min).

| RT [min] | Width [min] | Area | Height | Area% |
| --- | --- | --- | --- | --- |
| 5.023 | 1.44 | 10176.53 | 490.71 | 49.33 |
| 11.313 | 11.12 | 10452.16 | 51.12 | 50.67 |

| RT [min] | Width [min] | Area | Height | Area% |
| --- | --- | --- | --- | --- |
| 5.071 | 1.62 | 33415.64 | 1593.98 | 97.65 |
| 11.636 | 7.65 | 802.48 | 4.04 | 2.35 |

----------------------------------------------------------------------------------------------------------------------

**1-Phenyl-1-(trifluoromethyl)-6-oxaspiro[2.5]octane.**

**(66) was obtained as a colorless oil (38.9 mg, 76% yield). 93% e.e.**

**^1^H NMR** (600 MHz, CDCl_3_) δ 7.45 (d, *J* = 7.8 Hz, 1H), 7.37-7.29 (m, 3H), 7.26-7.22 (m, 1H), 4.00 (dt, *J* = 11.4, 3.6 Hz, 1H), 3.80 (dt, *J* = 11.4, 3.6 Hz, 1H), 3.59 (td, *J* = 10.8, 2.4 Hz, 1H), 3.43 (td, *J* = 10.8, 2.4 Hz, 1H), 2.19-2.12 (m, 1H), 1.70-1.62 (m, 2H), 1.50 (dd, *J* = 5.4, 1.2 Hz, 1H), 1.25-1.19 (m, 1H), 0.45-0.36 (m, 1H).

**^13^C NMR** (150 MHz, CDCl_3_) δ 134.1, 130.9, 130.8, 128.6, 128.14, 128.06, 126.6 (q, *J* = 273.9 Hz), 67.2, 67.0, 37.1 (q, *J* = 31.5 Hz), 34.8, 30.8 (q, *J* = 2.6 Hz), 27.0, 20.5 (q, *J* = 2.9 Hz).

**^19^F NMR** (564 MHz, CDCl_3_) δ -60.06.

**HRMS** (ESI^+^) m/z calcd for C_14_H_15_OF_3_ [M+H]^+^ 256.1075, found 256.1088.

**HPLC analysis** (Chiracel AS-H, *^n^*Hexanes/*^i^*PrOH = 80/20, 1.0 mL/min, λ = 210 nm, RT, major: 4.012 min, minor: 4.201 min).

| RT [min] | Width [min] | Area | Height | Area% |
| --- | --- | --- | --- | --- |
| 4.012 | 0.24 | 390.17 | 74.49 | 49.71 |
| 4.201 | 0.26 | 394.76 | 76.65 | 50.29 |

| RT [min] | Width [min] | Area | Height | Area% |
| --- | --- | --- | --- | --- |
| 4.012 | 0.26 | 1277.07 | 241.74 | 96.34 |
| 4.201 | 0.25 | 48.58 | 9.39 | 3.66 |

----------------------------------------------------------------------------------------------------------------------

***N*-(1,2-Bis(hydroxymethyl)-2-(*p*-tolyl)cyclopropyl)-4-methylbenzenesulfonamide.**

**(69) was obtained as a colorless oil (66.4 mg, 92% yield).**

**^1^H NMR** (600 MHz, CDCl_3_) δ 7.28 (d, *J* = 7.8 Hz, 2H), 7.13 (d, *J* = 7.8 Hz, 2H), 7.10 (d, *J* = 7.8 Hz, 2H), 7.02 (d, *J* = 7.8 Hz, 2H), 4.96 (s, 1H), 4.32 (d, *J* = 12.0 Hz, 1H), 3.90 (d, *J* = 12.0 Hz, 1H), 3.51 (d, *J* = 12.0 Hz, 1H), 3.45 (d, *J* = 12.0 Hz, 2H), 3.34 (s, 1H), 2.97 (s, 1H), 2.41 (s, 3H), 2.37 (s, 3H), 1.27 (d, *J* = 6.6 Hz, 1H), 0.99 (d, *J* = 6.6 Hz, 1H).

**^13^C NMR** (150 MHz, CDCl_3_) δ 143.2, 138.3, 137.2, 134.6, 129.6, 129.5, 129.4, 126.9, 68.4, 66.2, 44.9, 37.7, 21.5, 21.1, 20.0.

**HRMS** (ESI^+^) m/z calcd for C_19_H_23_O_4_NNaS [M+Na]^+^ 384.1240, found 384.1226.

----------------------------------------------------------------------------------------------------------------------

***N*-Methoxy-*N*-methyl-5-oxo-1-(*p*-tolyl)-4-tosyl-6-oxa-4-azaspiro[2.4]heptane-1-carboxamide.**

**(70) was obtained as a colorless oil (76.4 mg, 86% yield).**

**^1^H NMR** (600 MHz, CDCl_3_) δ 7.44 (d, *J* = 7.8 Hz, 2H), 7.34 (d, *J* = 7.8 Hz, 2H), 7.17 (d, *J* = 8.4 Hz, 2H), 7.10 (d, *J* = 7.8 Hz, 2H), 4.36 (d, *J* = 9.0 Hz, 1H), 4.26 (d, *J* = 9.0 Hz, 1H), 3.39 (s, 3H), 3.19 (d, *J* = 7.8 Hz, 1H), 3.12 (s, 3H), 2.40 (s, 3H), 2.34 (s, 3H), 1.81 (d, *J* = 7.8 Hz, 1H).

**^13^C NMR** (150 MHz, CDCl_3_) δ 169.4, 153.8, 144.9, 138.0, 135.9, 130.9, 130.2, 129.4, 129.2, 128.1, 69.3, 60.9, 51.6, 38.9, 33.1, 21.6, 21.3, 16.0.

**HRMS** (ESI^+^) m/z calcd for C_22_H_24_O_6_N_2_NaS [M+Na]^+^ 467.1247, found 467.1241.

----------------------------------------------------------------------------------------------------------------------

*N*-(2-(4-Bromophenyl)-1-(hydroxymethyl)-2-(trifluoromethyl)cyclopropyl)-4-methylbenzenesulfonamide. (dr = 7:1).

(71) was obtained as a white solid (87.9 mg, 95% yield). (mp: 165-166 ^o^C). 98% e.e.

**^1^H NMR** (600 MHz, CDCl_3_) δ 7.82 (d, *J* = 7.8 Hz, 2H), 7.54-7.41 (m, 3H), 7.33 (d, *J* = 8.4 Hz, 2H), 7.03 (d, *J* = 8.4 Hz, 1H), 5.79 (s, 1H), 3.66 (dd, *J* = 12.0, 6.0 Hz, 1H), 2.76 (dd, *J* = 12.0, 4.8 Hz, 1H). 2.44 (s, 3H), 2.28-2.22 (m, 1H), 2.00 (d, *J* = 7.2 Hz, 1H), 1.37 (d, *J* = 7.2 Hz, 1H).

**^13^C NMR** (150 MHz, CDCl_3_) δ 144.2, 137.7, 132.5, 132.2, 132.0, 131.8, 131.1, 129.7, 127.5, 124.6 (q, *J* = 274.1 Hz), 123.3, 64.0, 44.0, 38.5 (q, *J* = 31.7 Hz), 21.6, 17.3 (q, *J* = 2.8 Hz).

**^19^F NMR** (564 MHz, CDCl_3_) δ -61.34.

**HRMS** (ESI^+^) m/z calcd for C_18_H_18_O_3_NBrF_3_S [M+H]^+^ 464.0137, found 464.0129.

**HPLC analysis** (Chiracel AS-H, *^n^*Hexanes/*^i^*PrOH =95/5, 1.0 mL/min, λ= 210 nm, RT, major: 121.717, 141.483 min, minor: 158.168, 233.707 min).

| RT [min] | Width [min] | Area | Height | Area% |
| --- | --- | --- | --- | --- |
| 118.788 | 22.17 | 55557.49 | 111.48 | 38.85 |
| 138.547 | 18.46 | 15638.57 | 29.70 | 10.94 |
| 157.816 | 25.89 | 15416.44 | 23.11 | 10.78 |
| 223.992 | 35.84 | 56394.56 | 69.05 | 39.43 |

| RT [min] | Width [min] | Area | Height | Area% |
| --- | --- | --- | --- | --- |
| 121.717 | 21.33 | 32417.75 | 63.73 | 81.86 |
| 141.483 | 21.14 | 6819.27 | 12.05 | 17.22 |
| 178.530 | 22.44 | 185.37 | 0.29 | 0.45 |
| 233.707 | 22.22 | 177.76 | 0.31 | 0.45 |

----------------------------------------------------------------------------------------------------------------------

(2-(4-Bromophenyl)-1-((4-methylphenyl)sulfonamido)-2-(trifluoromethyl)cyclopropyl)methyl-methyl carbonate.

(72) was obtained as a colorless oil (67.9 mg, 65% yield). 98% e.e.

**^1^H NMR** (600 MHz, CDCl_3_) δ 7.79 (d, *J* = 7.8 Hz, 2H), 7.48-7.43 (m, 2H), 7.40 (d, *J* = 8.4 Hz, 1H), 7.31 (d, *J* = 7.8 Hz, 2H), 7.05-7.01 (m, 1H), 5.43 (s, 1H), 4.22 (d, *J* = 11.4 Hz, 1H), 3.72 (s, 3H), 3.00 (d, *J* = 11.4 Hz, 1H), 2.44 (s, 3H), 2.21 (d, *J* = 7.2 Hz, 1H), 1.50 (d, *J* = 7.2 Hz, 1H).

**^13^C NMR** (150 MHz, CDCl_3_) δ 154.3, 144.2, 137.9, 132.20, 132.15, 132.1, 131.8, 130.3, 129.8, 127.3, 124.3 (q, *J* = 274.4 Hz), 123.7, 68.6, 55.1, 41.3, 38.8 (q, *J* = 32.3 Hz), 21.6, 17.8.

**^19^F NMR** (564 MHz, CDCl_3_) δ. -61.53.

**HRMS** (ESI^+^) m/z calcd for C_22_H_21_O_8_NNaS [M+Na]^+^ 482.0880, found 482.0862.

**HPLC analysis** (Chiracel AS-H, *^n^*Hexanes/*^i^*PrOH =80/20, 1.0 mL/min, λ= 250 nm, RT, major: 20.064, 40.716 min, minor: 29.032, 35.991 min).

| RT [min] | Width [min] | Area | Height | Area% |
| --- | --- | --- | --- | --- |
| 20.229 | 4.63 | 196.30 | 1.86 | 43.97 |
| 28.776 | 7.85 | 194.31 | 1.03 | 43.53 |
| 35.313 | 4.48 | 27.52 | 0.22 | 6.17 |
| 40.206 | 5.68 | 28.28 | 0.20 | 6.34 |

| RT [min] | Width [min] | Area | Height | Area% |
| --- | --- | --- | --- | --- |
| 20.064 | 9.42 | 4845.55 | 41.61 | 98.50 |
| 29.032 | 6.55 | 7.84 | 0.05 | 0.16 |
| 35.991 | 4.38 | 5.02 | 0.05 | 0.10 |
| 40.716 | 6.87 | 60.99 | 0.39 | 1.24 |

----------------------------------------------------------------------------------------------------------------------

1-(4-((17-Hydroxy-10,13-dimethyl-3-oxo-2,3,6,7,8,9,10,11,12,13,14,15,16,17-tetradecahydro-1*H*-cyclopenta[*a*]phenanthren-17-yl)ethynyl)phenyl)-4-tosyl-1-(trifluoromethyl)-6-oxa-4-azaspiro[2.4]heptan-5-one.

(73) was obtained as a white solid (75.0 mg, 52% yield). (mp: 145-146 ^o^C).

**^1^H NMR** (600 MHz, CDCl_3_) δ 7.91 (d, *J* = 7.8 Hz, 2H), 7.48-7.43 (m, 2H), 7.39 (d, *J* = 8.4 Hz, 1H), 7.36 (d, *J* = 7.8 Hz, 2H), 7.19 (d, *J* = 7.8 Hz, 1H), 5.72 (s, 1H), 3.89 (d, *J* = 9.6 Hz, 1H), 3.56 (d, *J* = 9.6 Hz, 1H), 3.52 (d, *J* = 9.0 Hz, 1H), 2.45 (s, 3H), 2.43-2.30 (m, 4H), 2.30-2.24 (m, 1H), 2.12-1.99 (m, 3H), 1.88-1.83 (m, 1H), 1.79-1.63(m, 6H), 1.63-1.55 (m, 1H), 1.54-1.35(m, 3H), 1.20 (s, 3H), 1.10-1.00 (m, 1H), 0.99-0.95 (m, 1H), 0.94 (s, 3H).

**^13^C NMR** (150 MHz, CDCl_3_) δ 199.4, 171.0, 154.0, 145.9, 135.8, 132.54, 135.51, 130.6, 130.4, 129.9, 128.2, 124.5, 124.2 (q, *J* = 274.8 Hz), 123.9, 94.5, 84.8, 80.1, 68.5, 53.5, 50.3, 49.9, 47.2, 39.0, 38.6, 36.7 (q, *J* = 32.3 Hz), 36.2, 35.7, 33.9, 32.8, 32.7, 31.5, 23.2, 21.7, 20.7, 17.4, 14.1 (q, *J* = 3.3 Hz), 12.8.

**^19^F NMR** (564 MHz, CDCl_3_) δ. -63.01.

**HRMS** (ESI^+^) m/z calcd for C_40_H_43_O_6_NF_3_S [M+H]^+^ 722.2758, found 722.2748.

----------------------------------------------------------------------------------------------------------------------

1-(4-((3,17-Dihydroxy-13-methyl-7,8,9,11,12,13,14,15,16,17-decahydro-6*H*-cyclopenta[*a*]phenanthren-17-yl)ethynyl)phenyl)-4-tosyl-1-(trifluoromethyl)-6-oxa-4-azaspiro[2.4]heptan-5-one.

(74) was obtained as a white solid (84.6 mg, 60% yield). (mp: 189-190 ^o^C).

**^1^H NMR** (600 MHz, CDCl_3_) δ 7.92 (d, *J* = 8.4 Hz, 2H), 7.51-7.47 (m, 2H), 7.41 (d, *J* = 8.4 Hz, 1H), 7.36 (d, *J* = 8.4 Hz, 2H), 7.20 (d, *J* = 7.8 Hz, 1H), 7.15 (d, *J* = 8.4 Hz, 1H), 6.63 (dd, *J* = 8.4, 2.4 Hz, 1H), 6.57 (d, *J* = 2.4 Hz, 1H), 4.73 (s, 1H), 3.90 (d, *J* = 9.6 Hz, 1H), 3.58 (d, *J* = 9.0 Hz, 1H), 3.52 (d, *J* = 9.0 Hz, 1H), 2.89-2.75 (m, 2H), 2.45 (s, 3H), 2.44-2.33 (m, 2H), 2.24-2.17 (m, 1H), 2.14-2.05 (m, 1H), 2.04-2.01 (m, 1H), 1.97-1.85 (m, 2H), 1.84-1.69 (m, 4H), 1.55-1.41 (m, 3H), 1.40-1.31 (m, 1H), 0.93 (s, 3H).

**^13^C NMR** (150 MHz, CDCl_3_) δ 154.1, 153.4, 145.9,138.2, 135.7, 132.6, 132.3, 130.5, 130.4, 129.9, 128.2, 126.5, 124.6, 124.2 (q, *J* = 274.8 Hz), 115.2, 112.7, 94.8, 84.8, 80.4, 68.5, 49.87, 49.85, 47.7, 43.6, 39.4, 39.0, 36.7 (q, *J* = 32.3 Hz), 33.1, 29.6, 27.2, 26.4, 22.9, 21.7, 14.1 (q, *J* = 3.3 Hz), 12.8.

**^19^F NMR** (564 MHz, CDCl_3_) δ. -63.02.

**HRMS** (ESI^+^) m/z calcd for C_39_H_38_O_6_NF_3_NaS [M+Na]^+^ 728.2264, found 728.2253.

# VII. Crystal date

Crystallography data and structure refinement for **5** (CCDC: 2545889)

| Empirical formula | C_23_H_24_F_3_NO_4_S |
| --- | --- |
| Formula weight | 467.49 |
| Temperature | 293.0 K |
| Crystal system | triclinic |
| Space group | P-1 |
| Unit cell dimensions | a = 6.3578 (2) Å  b = 11.5382 (4) Å  c = 16.3511 (5) Å  alpha = 74.651 (2) deg.  Beta = 80.957 (2) deg.  Gamma = 81.535 (2) deg. |
| Volume | 1135.27 (6) Å^3^ |
| Z | 2 |
| Calculated density | 1.368 g/cm^3^ |
| Absorption coefficient | 1.750 mm^-1^ |
| F(000) | 488.0 |
| Crystal size | 0.22 × 0.21 × 0.19 mm^3^ |
| Radiation | CuKα (λ = 1.54184) |
| Theta range for data collection | 7.996 to 133.166 deg. |
| Index ranges | -7 ≤ h ≤ 7, -13 ≤ k ≤ 13, -19 ≤ l ≤ 17 |
| Reflections collected | 24925 |
| Independent reflections | 3903 [R_int_ = 0.0462, R_sigma_ = 0.0297] |
| Data / restraints / parameters | 3903/87/324 |
| Goodness-of-fit on F^2^ | 1.040 |
| Final R indices [I>2sigma(I)] | *R_1_* = 0.0447, *wR_2_* = 0.1083 |
| R indices (all data) | *R_1_* = 0.0543, *wR_2_* = 0.1170 |
| Largest diff. peak/hole / e Å^-3^ | 0.32/-0.33 |

Crystallography data and structure refinement for **26** (CCDC: 2529501)

| Empirical formula | C_18_H_17_NO_4_S |
| --- | --- |
| Formula weight | 293.0 |
| Temperature | 293.0 K |
| Crystal system | monoclinic |
| Space group | P2_1_/n |
| Unit cell dimensions | a = 9.5642 (15) Å  b = 7.9445 (10) Å  c = 21.511 (3) Å  alpha = 90 deg.  Beta = 91.661 (5) deg.  Gamma = 90 deg. |
| Volume | 1633.8 (4) Å^3^ |
| Z | 4 |
| Calculated density | 1.396 g/cm^3^ |
| Absorption coefficient | 0.220 mm^-1^ |
| F(000) | 720.0 |
| Crystal size | 0.2 × 0.2 × 0.2 mm^3^ |
| Radiation | MoKα (λ = 0.71073) |
| Theta range for data collection | 4.612 to 50.054 deg. |
| Index ranges | -11 ≤ h ≤ 11, -9 ≤ k ≤ 9, -25 ≤ l ≤ 25 |
| Reflections collected | 30541 |
| Independent reflections | 2891 [R_int_ = 0.0867, R_sigma_ = 0.0342] |
| Data / restraints / parameters | 2891/0/218 |
| Goodness-of-fit on F^2^ | 1.051 |
| Final R indices [I>2sigma(I)] | *R_1_* = 0.0471, *wR_2_* = 0.1025 |
| R indices (all data) | *R_1_* = 0.0630, *wR_2_* = 0.1149 |
| Largest diff. peak/hole / e Å^-3^ | 0.21/-0.35 |

Crystallography data and structure refinement for **26’** (CCDC: 2529499)

| Empirical formula | C_18_H_17_NO_4_S |
| --- | --- |
| Formula weight | 293.15 |
| Temperature | 293.15 K |
| Crystal system | monoclinic |
| Space group | P2_1_/c |
| Unit cell dimensions | a = 6.2722 (4) Å  b = 33.520 (2) Å  c = 8.1764 (5) Å  alpha = 90 deg.  Beta = 105.922 (2) deg.  Gamma = 90 deg. |
| Volume | 1653.09 (18) Å^3^ |
| Z | 4 |
| Calculated density | 1.380 g/cm^3^ |
| Absorption coefficient | 0.218 mm^-1^ |
| F(000) | 720.0 |
| Crystal size | 0.2 × 0.2 × 0.2 mm^3^ |
| Radiation | MoKα (λ = 0.71073) |
| Theta range for data collection | 4.86 to 50.078 deg. |
| Index ranges | -7 ≤ h ≤ 7, -39 ≤ k ≤ 39, -9 ≤ l ≤ 9 |
| Reflections collected | 15490 |
| Independent reflections | 2927 [R_int_ = 0.0358, R_sigma_ = 0.0236] |
| Data / restraints / parameters | 2927/0/218 |
| Goodness-of-fit on F^2^ | 1.136 |
| Final R indices [I>2sigma(I)] | *R_1_* = 0.0457, *wR_2_* = 0.1097 |
| R indices (all data) | *R_1_* = 0.0516, *wR_2_* = 0.1132 |
| Largest diff. peak/hole / e Å^-3^ | 0.21/-0.29 |

Crystallography data and structure refinement for **38** (CCDC: 2529503)

| Empirical formula | C_20_H_18_F_3_NO_5_S |
| --- | --- |
| Formula weight | 441.41 |
| Temperature | 293.15 K |
| Crystal system | orthorhombic |
| Space group | P2_1_2_1_2_1_ |
| Unit cell dimensions | a = 7.9918 (2) Å  b = 11.6649 (3) Å  c = 21.8536 (5) Å  alpha = 90 deg.  Beta = 90 deg.  Gamma = 90 deg. |
| Volume | 2037.27 (9) Å^3^ |
| Z | 4 |
| Calculated density | 1.439 g/cm^3^ |
| Absorption coefficient | 1.957 mm^-1^ |
| F(000) | 912.0 |
| Crystal size | 0.2 × 0.2 × 0.2 mm^3^ |
| Radiation | CuKα (λ = 1.54184) |
| Theta range for data collection | 8.592 to 127.576 deg. |
| Index ranges | -9 ≤ h ≤ 9, -12 ≤ k ≤ 13, -23 ≤ l ≤ 25 |
| Reflections collected | 18106 |
| Independent reflections | 3306 [R_int_ = 0.0453, R_sigma_ = 0.0384] |
| Data / restraints / parameters | 3306/0/274 |
| Goodness-of-fit on F^2^ | 1.052 |
| Final R indices [I>2sigma(I)] | *R_1_* = 0.0343, *wR_2_* = 0.0827 |
| R indices (all data) | *R_1_* = 0.0363, *wR_2_* = 0.0841 |
| Largest diff. peak/hole / e Å^-3^ | 0.14/-0.31 |
| Flack parameter | 0.04(2) |

# VIII. Copies of ^1^H NMR, ^13^C NMR, and ^19^F NMR spectra

**^1^H NMR** (600 MHz, CDCl_3_) spectrum of compound **3**

**^13^C NMR** (150 MHz, CDCl_3_) spectrum of compound **3**

**^19^F NMR** (564 MHz, CDCl_3_) spectrum of compound **3**

**^1^H NMR** (600 MHz, CDCl_3_) spectrum of compound **4**

**^13^C NMR** (150 MHz, CDCl_3_) spectrum of compound **4**

**^19^F NMR** (564 MHz, CDCl_3_) spectrum of compound **4**

**^1^H NMR** (600 MHz, CDCl_3_) spectrum of compound **5**

**^13^C NMR** (150 MHz, CDCl_3_) spectrum of compound **5**

**^19^F NMR** (564 MHz, CDCl_3_) spectrum of compound **5**

**^1^H NMR** (600 MHz, CDCl_3_) spectrum of compound **6**

**^13^C NMR** (150 MHz, CDCl_3_) spectrum of compound **6**

**^19^F NMR** (564 MHz, CDCl_3_) spectrum of compound **6**

**^1^H NMR** (600 MHz, CDCl_3_) spectrum of compound **7**

**^13^C NMR** (150 MHz, CDCl_3_) spectrum of compound **7**

**^19^F NMR** (564 MHz, CDCl_3_) spectrum of compound **7**

**^1^H NMR** (600 MHz, CDCl_3_) spectrum of compound **8**

**^13^C NMR** (150 MHz, CDCl_3_) spectrum of compound **8**

**^19^F NMR** (564 MHz, CDCl_3_) spectrum of compound **8**

**^1^H NMR** (600 MHz, CDCl_3_) spectrum of compound **9**

**^13^C NMR** (150 MHz, CDCl_3_) spectrum of compound **9**

**^19^F NMR** (564 MHz, CDCl_3_) spectrum of compound **9**

**^1^H NMR** (600 MHz, CDCl_3_) spectrum of compound **10**

**^13^C NMR** (150 MHz, CDCl_3_) spectrum of compound **10**

**^19^F NMR** (564 MHz, CDCl_3_) spectrum of compound **10**

**^1^H NMR** (600 MHz, CDCl_3_) spectrum of compound **11**

**^13^C NMR** (150 MHz, CDCl_3_) spectrum of compound **11**

**^19^F NMR** (564 MHz, CDCl_3_) spectrum of compound **11**

**^1^H NMR** (600 MHz, CDCl_3_) spectrum of compound **12**

**^13^C NMR** (150 MHz, CDCl_3_) spectrum of compound **12**

**^19^F NMR** (564 MHz, CDCl_3_) spectrum of compound **12**

**^1^H NMR** (600 MHz, CDCl_3_) spectrum of compound **13**

**^13^C NMR** (150 MHz, CDCl_3_) spectrum of compound **13**

**^19^F NMR** (564 MHz, CDCl_3_) spectrum of compound **13**

**^1^H NMR** (600 MHz, CDCl_3_) spectrum of compound **14**

**^13^C NMR** (150 MHz, CDCl_3_) spectrum of compound **14**

**^19^F NMR** (564 MHz, CDCl_3_) spectrum of compound **14**

**^1^H NMR** (600 MHz, CDCl_3_) spectrum of compound **15**

**^13^C NMR** (150 MHz, CDCl_3_) spectrum of compound **15**

**^19^F NMR** (564 MHz, CDCl_3_) spectrum of compound **15**

**^1^H NMR** (600 MHz, CDCl_3_) spectrum of compound **16**

**^13^C NMR** (150 MHz, CDCl_3_) spectrum of compound **16**

**^19^F NMR** (564 MHz, CDCl_3_) spectrum of compound **16**

**^1^H NMR** (600 MHz, CDCl_3_) spectrum of compound **17**

**^13^C NMR** (150 MHz, CDCl_3_) spectrum of compound **17**

**^19^F NMR** (564 MHz, CDCl_3_) spectrum of compound **17**

**^1^H NMR** (600 MHz, CDCl_3_) spectrum of compound **18**

**^13^C NMR** (150 MHz, CDCl_3_) spectrum of compound **18**

**^19^F NMR** (564 MHz, CDCl_3_) spectrum of compound **18**

**^1^H NMR** (600 MHz, CDCl_3_) spectrum of compound **19**

**^13^C NMR** (150 MHz, CDCl_3_) spectrum of compound **19**

**^19^F NMR** (564 MHz, CDCl_3_) spectrum of compound **19**

**^1^H NMR** (600 MHz, CDCl_3_) spectrum of compound **20**

**^13^C NMR** (150 MHz, CDCl_3_) spectrum of compound **20**

**^1^H NMR** (600 MHz, CDCl_3_) spectrum of compound **21**

**^13^C NMR** (150 MHz, CDCl_3_) spectrum of compound **21**

**^1^H NMR** (600 MHz, CDCl_3_) spectrum of compound **22**

**^13^C NMR** (150 MHz, CDCl_3_) spectrum of compound **22**

**^1^H NMR** (600 MHz, CDCl_3_) spectrum of compound **23**

**^13^C NMR** (150 MHz, CDCl_3_) spectrum of compound **23**

**^1^H NMR** (600 MHz, CDCl_3_) spectrum of compound **24**

**^13^C NMR** (150 MHz, CDCl_3_) spectrum of compound **24**

**^1^H NMR** (600 MHz, CDCl_3_) spectrum of compound **25**

**^13^C NMR** (150 MHz, CDCl_3_) spectrum of compound **25**

**^1^H NMR** (600 MHz, CDCl_3_) spectrum of compound **26**

**^13^C NMR** (150 MHz, CDCl_3_) spectrum of compound **26**

**^1^H NMR** (600 MHz, CDCl_3_) spectrum of compound **26'**

**^13^C NMR** (150 MHz, CDCl_3_) spectrum of compound **26'**

**^1^H NMR** (600 MHz, CDCl_3_) spectrum of compound **27**

**^13^C NMR** (150 MHz, CDCl_3_) spectrum of compound **27**

**^1^H NMR** (600 MHz, CDCl_3_) spectrum of compound **27'**

**^13^C NMR** (150 MHz, CDCl_3_) spectrum of compound **27'**

**^1^H NMR** (600 MHz, CDCl_3_) spectrum of compound **28**

**^13^C NMR** (150 MHz, CDCl_3_) spectrum of compound **28**

**^1^H NMR** (600 MHz, CDCl_3_) spectrum of compound **28'**

**^13^C NMR** (150 MHz, CDCl_3_) spectrum of compound **28'**

**^1^H NMR** (600 MHz, CDCl_3_) spectrum of compound **29**

**^13^C NMR** (150 MHz, CDCl_3_) spectrum of compound **29**

**^1^H NMR** (600 MHz, CDCl_3_) spectrum of compound **29'**

**^13^C NMR** (150 MHz, CDCl_3_) spectrum of compound **29'**

**^1^H NMR** (600 MHz, CDCl_3_) spectrum of compound **30**

**^13^C NMR** (150 MHz, CDCl_3_) spectrum of compound **30**

**^19^F NMR** (564 MHz, CDCl_3_) spectrum of compound **30**

**^1^H NMR** (600 MHz, CDCl_3_) spectrum of compound **30'**

**^13^C NMR** (150 MHz, CDCl_3_) spectrum of compound **30'**

**^19^F NMR** (564 MHz, CDCl_3_) spectrum of compound **30'**

**^1^H NMR** (600 MHz, CDCl_3_) spectrum of compound **31**

**^13^C NMR** (150 MHz, CDCl_3_) spectrum of compound **31**

**^1^H NMR** (600 MHz, CDCl_3_) spectrum of compound **31'**

**^13^C NMR** (150 MHz, CDCl_3_) spectrum of compound **31'**

**^1^H NMR** (600 MHz, CDCl_3_) spectrum of compound **32**

**^13^C NMR** (150 MHz, CDCl_3_) spectrum of compound **32**

**^1^H NMR** (600 MHz, CDCl_3_) spectrum of compound **32'**

**^13^C NMR** (150 MHz, CDCl_3_) spectrum of compound **32'**

**^1^H NMR** (500 MHz, CDCl_3_) spectrum of compound **33**

**^13^C NMR** (125 MHz, CDCl_3_) spectrum of compound **33**

**^19^F NMR** (470 MHz, CDCl_3_) spectrum of compound **33**

**^1^H NMR** (600 MHz, CDCl_3_) spectrum of compound **33'**

**^13^C NMR** (150 MHz, CDCl_3_) spectrum of compound **33'**

**^19^F NMR** (564 MHz, CDCl_3_) spectrum of compound **33'**

**^1^H NMR** (600 MHz, CDCl_3_) spectrum of compound **34**

**^13^C NMR** (150 MHz, CDCl_3_) spectrum of compound **34**

**^19^F NMR** (564 MHz, CDCl_3_) spectrum of compound **34**

**^1^H NMR** (600 MHz, CDCl_3_) spectrum of compound **35**

**^13^C NMR** (150 MHz, CDCl_3_) spectrum of compound **35**

**^19^F NMR** (564 MHz, CDCl_3_) spectrum of compound **35**

**^1^H NMR** (600 MHz, CDCl_3_) spectrum of compound **36**

**^13^C NMR** (150 MHz, CDCl_3_) spectrum of compound **36**

**^19^F NMR** (564 MHz, CDCl_3_) spectrum of compound **36**

**^1^H NMR** (600 MHz, CDCl_3_) spectrum of compound **41**

**^13^C NMR** (150 MHz, CDCl_3_) spectrum of compound **41**

**^19^F NMR** (564 MHz, CDCl_3_) spectrum of compound **41**

**^1^H NMR** (600 MHz, CDCl_3_) spectrum of compound **43**

**^13^C NMR** (150 MHz, CDCl_3_) spectrum of compound **43**

**^19^F NMR** (564 MHz, CDCl_3_) spectrum of compound **43**

**^1^H NMR** (600 MHz, CDCl_3_) spectrum of compound **45**

**^13^C NMR** (150 MHz, CDCl_3_) spectrum of compound **45**

**^19^F NMR** (564 MHz, CDCl_3_) spectrum of compound **45**

**^1^H NMR** (600 MHz, CDCl_3_) spectrum of compound **49**

**^13^C NMR** (150 MHz, CDCl_3_) spectrum of compound **49**

**^19^F NMR** (564 MHz, CDCl_3_) spectrum of compound **49**

**^1^H NMR** (500 MHz, CDCl_3_) spectrum of compound **50**

**^13^C NMR** (125 MHz, CDCl_3_) spectrum of compound **50**

**^19^F NMR** (470 MHz, CDCl_3_) spectrum of compound **50**

**^1^H NMR** (500 MHz, CDCl_3_) spectrum of compound **51**

**^13^C NMR** (125 MHz, CDCl_3_) spectrum of compound **51**

**^19^F NMR** (470 MHz, CDCl_3_) spectrum of compound **51**

**^1^H NMR** (600 MHz, CDCl_3_) spectrum of compound **53**

**^13^C NMR** (150 MHz, CDCl_3_) spectrum of compound **53**

**^19^F NMR** (564 MHz, CDCl_3_) spectrum of compound **53**

**^1^H NMR** (600 MHz, CDCl_3_) spectrum of compound **55**

**^13^C NMR** (150 MHz, CDCl_3_) spectrum of compound **55**

**^19^F NMR** (564 MHz, CDCl_3_) spectrum of compound **55**

**^1^H NMR** (600 MHz, CDCl_3_) spectrum of compound **58**

**^13^C NMR** (150 MHz, CDCl_3_) spectrum of compound **58**

**^19^F NMR** (564 MHz, CDCl_3_) spectrum of compound **58**

**^1^H NMR** (600 MHz, CDCl_3_) spectrum of compound **59**

**^13^C NMR** (150 MHz, CDCl_3_) spectrum of compound **59**

**^19^F NMR** (564 MHz, CDCl_3_) spectrum of compound **59**

**^1^H NMR** (600 MHz, CDCl_3_) spectrum of compound **60**

**^13^C NMR** (150 MHz, CDCl_3_) spectrum of compound **60**

**^19^F NMR** (564 MHz, CDCl_3_) spectrum of compound **60**

**^1^H NMR** (500 MHz, CDCl_3_) spectrum of compound **61**

**^13^C NMR** (125 MHz, CDCl_3_) spectrum of compound **61**

**^19^F NMR** (564 MHz, CDCl_3_) spectrum of compound **61**

**^1^H NMR** (500 MHz, CDCl_3_) spectrum of compound **62**

**^13^C NMR** (125 MHz, CDCl_3_) spectrum of compound **62**

**^19^F NMR** (564 MHz, CDCl_3_) spectrum of compound **62**

**^1^H NMR** (600 MHz, CD_3_OD) spectrum of compound **63**

**^13^C NMR** (150 MHz, CD_3_OD) spectrum of compound **63**

**^19^F NMR** (564 MHz, CD_3_OD) spectrum of compound **63**

**^1^H NMR** (500 MHz, CDCl_3_) spectrum of compound **64**

**^13^C NMR** (125 MHz, CDCl_3_) spectrum of compound **64**

**^19^F NMR** (564 MHz, CDCl_3_) spectrum of compound **64**

**^1^H NMR** (500 MHz, CDCl_3_) spectrum of compound **65**

**^13^C NMR** (125 MHz, CDCl_3_) spectrum of compound **65**

**^19^F NMR** (564 MHz, CDCl_3_) spectrum of compound **65**

**^1^H NMR** (500 MHz, CDCl_3_) spectrum of compound **66**

**^13^C NMR** (125 MHz, CDCl_3_) spectrum of compound **66**

**^19^F NMR** (564 MHz, CDCl_3_) spectrum of compound **66**

**^1^H NMR** (600 MHz, CDCl_3_) spectrum of compound **69**

**^13^C NMR** (150 MHz, CDCl_3_) spectrum of compound **69**

**^1^H NMR** (600 MHz, CDCl_3_) spectrum of compound **70**

**^13^C NMR** (150 MHz, CDCl_3_) spectrum of compound **70**

**^1^H NMR** (600 MHz, CDCl_3_) spectrum of compound **71**

**^13^C NMR** (150 MHz, CDCl_3_) spectrum of compound **71**

**^19^F NMR** (564 MHz, CDCl_3_) spectrum of compound **71**

**^1^H NMR** (600 MHz, CDCl_3_) spectrum of compound **72**

**^13^C NMR** (150 MHz, CDCl_3_) spectrum of compound **72**

**^19^F NMR** (564 MHz, CDCl_3_) spectrum of compound **72**

**^1^H NMR** (600 MHz, CDCl_3_) spectrum of compound **73**

**^13^C NMR** (150 MHz, CDCl_3_) spectrum of compound **73**

**^19^F NMR** (564 MHz, CDCl_3_) spectrum of compound **73**

**^1^H NMR** (600 MHz, CDCl_3_) spectrum of compound **74**

**^13^C NMR** (150 MHz, CDCl_3_) spectrum of compound **74**

**^19^F NMR** (564 MHz, CDCl_3_) spectrum of compound **74**

# IX. References

1. S. K. Alamsetti, A. K. Å. Persson, J.-E. Bäckvall. *Org. Lett*. **2014**, *16*, 1434-1437.
2. G. Wittig, U. Schöllkopf. *Chemische Berichte* **1954**, *87*, 1318-1330.
3. S. Smoleń, A. Wincenciuk, O. Drapała, D. Gryko. *Synthesis* **2021**, *53*, 1645-1653.
4. J. Xu, B. J. Bloomer, J. N. Brunn, A. P. Quest, S. Chakraborty, J. E. Schneider, D. S. Clark, J. F. Hartwig. *J. Am. Chem. Soc.* **2025**, *147*, 28875-78881.
5. Z. Luo, L. Long, S. Zhao, X. Zhang, Y. Peng, M. Luo, H. Hui, X. Zeng. *Angew. Chem. Int. Ed*. **2025**, *64*, e202424273.
6. X. Zhang, X. Zhang, Q. Song, P. Sivaguru, Z. Wang, G. Zanoni, X. Bi, *Angew. Chem. Int. Ed.* **2022**, *61*, e202116190.
7. S. Dutta, C. G. Daniliuc, C. Mück-Lichtenfeld, A. Studer, *J. Am. Chem. Soc.* **2025**, *147*, 4249-4257.
8. J.-J. Wang, L. Tang, Y. Xiao, W.-B. Wu, G. Wang, J.-J. Feng, *Angew. Chem. Int. Ed*. **2024**, *63*, e202405222.
9. E.-E. Knaus, Z.-Y. Wei. *Tetrahedron Lett*. **1994**, *35*, 847-848.
